# The peptidoglycan-associated protein NapA plays an important role in the envelope integrity and in the pathogenesis of the lyme disease spirochete

**DOI:** 10.1371/journal.ppat.1009546

**Published:** 2021-05-13

**Authors:** Marisela M. Davis, Aaron M. Brock, Tanner G. DeHart, Brittany P. Boribong, Katherine Lee, Mecaila E. McClune, Yunjie Chang, Nicholas Cramer, Jun Liu, Caroline N. Jones, Brandon L. Jutras

**Affiliations:** 1 Department of Biochemistry, Virginia Tech, Blacksburg, Virginia, United States of America; 2 Fralin Life Sciences Institute, Virginia Tech, Blacksburg, Virginia, United States of America; 3 Molecular and Cellular Biology, Virginia Tech, Blacksburg, Virginia, United States of America; 4 Genetics, Bioinformatics, and Computational Biology, Virginia Tech, Blacksburg, Virginia, United States of America; 5 Department of Biological Sciences, Virginia Tech, Blacksburg, Virginia, United States of America; 6 Department of Microbial Pathogenesis, Yale School of Medicine, New Haven, Connecticut, United States of America; 7 Microbial Sciences Institute, Yale University, West Haven, Connecticut, United States of America; 8 Center for Emerging, Zoonotic and Arthropod-borne Pathogens, Virginia Tech, Blacksburg, Virginia, United States of America; 9 Translational Biology, Medicine, and Health, Virginia Tech, Blacksburg, Virginia, United States of America; University of Montana, UNITED STATES

## Abstract

The bacterial pathogen responsible for causing Lyme disease, *Borrelia burgdorferi*, is an atypical Gram-negative spirochete that is transmitted to humans via the bite of an infected *Ixodes* tick. In diderms, peptidoglycan (PG) is sandwiched between the inner and outer membrane of the cell envelope. In many other Gram-negative bacteria, PG is bound by protein(s), which provide both structural integrity and continuity between envelope layers. Here, we present evidence of a peptidoglycan-associated protein (PAP) in *B*. *burgdorferi*. Using an unbiased proteomics approach, we identified Neutrophil Attracting Protein A (NapA) as a PAP. Interestingly, NapA is a Dps homologue, which typically functions to bind and protect cellular DNA from damage during times of stress. While *B*. *burgdorferi* NapA is known to be involved in the oxidative stress response, it lacks the critical residues necessary for DNA binding. Biochemical and cellular studies demonstrate that NapA is localized to the *B*. *burgdorferi* periplasm and is indeed a PAP. Cryo-electron microscopy indicates that mutant bacteria, unable to produce NapA, have structural abnormalities. Defects in cell-wall integrity impact growth rate and cause the *napA* mutant to be more susceptible to osmotic and PG-specific stresses. NapA-linked PG is secreted in outer membrane vesicles and augments IL-17 production, relative to PG alone. Using microfluidics, we demonstrate that NapA acts as a molecular beacon—exacerbating the pathogenic properties of *B*. *burgdorferi* PG. These studies further our understanding of the *B*. *burgdorferi* cell envelope, provide critical information that underlies its pathogenesis, and highlight how a highly conserved bacterial protein can evolve mechanistically, while maintaining biological function.

## Author summary

Diderms typically produce peptidoglycan-associated proteins (PAPs) to enhance structural integrity and continuity within the cell envelope. We have identified a PAP in *B*. *burgdorferi* with structural and sequence homology to the near ubiquitous bacterial protein Dps (DNA binding protein from starved bacteria). The *B*. *burgdorferi* Dps paralogue is incapable of binding DNA. Instead, it bolsters the protective properties of the PG layer, while playing an important role in host immune modulation. Collectively, our findings highlight the plasticity of bacterial proteins in that they may change how they perform a task despite maintaining the same basic biological function.

## Introduction

The spirochetal bacterium *Borrelia burgdorferi* is the primary agent of Lyme disease, a debilitating infection that is transmitted to humans by the bite of an infected *Ixodes spp*. of tick. Over the past 20 years in the United States, the incidence of Lyme disease has increased more than 2000 percent with an estimate of close to 476,000 patients diagnosed annually [1,2]. Increases in disease prevalence can be attributed to 1) geographical expansion of vector ticks; 2) higher pathogen carriage rates; 3) deforestation; 4) increase in physician awareness of Lyme disease; and 5) social behavior [3–5]. Given the number of complex variables contributing to the ascendency of Lyme disease, this pervasive problem is likely to continue for the foreseeable future.

Upon transmission from an infected tick to a human host, *B*. *burgdorferi* causes a biphasic infection with a variety of clinical manifestations [6]. Acute, localized infection results in vague symptoms including fever, myalgias and headaches with the notable exception of an *erythema migrans* ‘Bullseye-like’ rash [6,7]. If not promptly and properly treated, patients may go on to experience late-stage disease complications that affect many tissues and organ systems [8]. Lyme arthritis (LA)—proliferative synovitis of one or more large joints—is the most common late-stage manifestation of Lyme disease in the United States [3,9]. LA progression and symptom persistence are multi-factorial [10]. For example, adaptive autoantibodies to bacterial products correlate with LA severity [11–14] while genetic polymorphisms in humoral receptors make some more susceptible to adverse outcomes [15]. In addition, a recent discovery has implicated remnants of the bacterial cell envelope as a likely contributor to disease pathology [16]. How these factors are connected, the consequences of their interplay, and other components that may contribute to the development and persistence of LA, are not known.

The typical Gram-negative cell envelope consists of an outer membrane (OM), an inner membrane (IM), and the periplasmic space in between. One essential component of the cell envelope—peptidoglycan—resides in the periplasm. Peptidoglycan (PG) is a gigadalton-sized biopolymer made up of rigid glycan strands composed of the repeating disaccharide *N-*acetylglucosamine (Glc*N*Ac) and *N-*acetylmuramic acid (Mur*N*Ac), that are cross-linked by short peptides [17,18]. The primary function of PG is to protect the cell from bursting due to the high osmotic pressure created by the crowded bacterial cytoplasm [19]. PG position within the periplasm is critical to its protective properties. It is perhaps not surprising then that most diderms produce highly conserved proteins that precisely position PG relative to the other envelope components. Bacteria unable to produce peptidoglycan-associated proteins (PAPs), have severe defects in cell 1) growth; 2) division; 3) morphology; 4) communication; and 5) ability to withstand exogenous stress [20,21]. Interestingly, many of these seemingly structural cell-wall components moonlight as virulence factors that contribute to bacterial pathogenicity [20,22,23].

Relative to classical diderms, the *B*. *burgdorferi* cell envelope is riddled with anomalies. For example, despite being a diderm, *B*. *burgdorferi* does not produce Lipopolysaccharide [24–26]. The outer membrane (OM) contains host-derived cholesterol [27,28] and more than 100 different lipoproteins [29]. Flagella are not extruded from the envelope, but rather are contained entirely in the periplasmic space [30,31]. Cross-linking peptides in the PG cell-wall contain the atypical diamine L-Ornithine [16,32]. Further, the typical proteins which are associated with PG that provide both structural integrity and spatial continuity within the cell envelope, appear to be lacking. Here, we describe the identification of a *B*. *burgdorferi* PAP, previously implicated as an immunomodulatory factor and determine its function in the cell envelope homeostasis. In addition, we provide evidence for a unique PG-PAP relationship that likely contributes to the pathogenic properties of *B*. *burgdorferi* PG.

## Results

### Identification of PAPS

**Fig 1:**
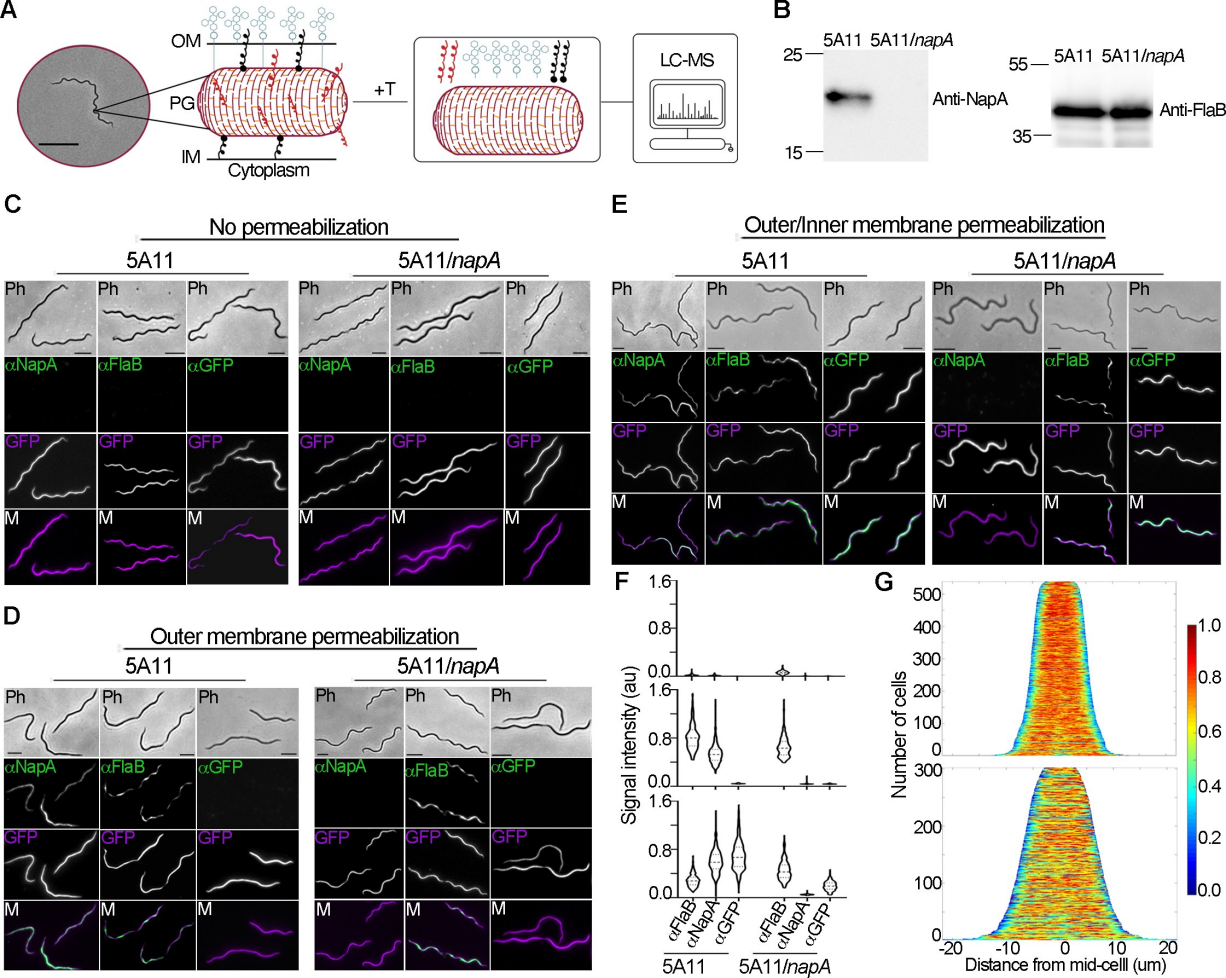
Identification of peptidoglycan-associated proteins (PAPs) in *B*. *burgdorferi*. (A) Peptidoglycan was isolated from live *B*. *burgdorferi* (phase-contrast micrograph, scale bar 5μm) and treated with trypsin (+T). Peptides from each preparation were identified by LC-MS. (B) Western blot analysis of whole cell lysates prepared from the parental, wild-type strain (5A11) and *napA* mutant (5A11/*napA)*. Each preparation was assayed by western blot for NapA (left) and the constitutive protein FlaB (right). The latter served as a loading control. Numbers and dashes correspond to the migration of molecular weight standards. (C-E) Localization of putative PAP NapA by sub-cellular fractionation coupled with immunofluorescence. Both 5A11 and 5A11/*napA* were transformed with a *B*. *burgdorferi* shuttle vector constitutively expressing GFP (GFP, purple panel). Each strain was fixed and treated with sodium phosphate buffer (no permeabilization, C); 50% methanol (outer membrane permeabilization, D); or methanol, followed by SDS and lysozyme (outer/inner membrane permeabilization, E). Both strains, treated with each permeabilization method, were probed for three targets, independently: Anti-FlaB (periplasmic control, green), anti-GFP (cytoplasmic control, green), and anti-NapA (green). Secondary antibodies anti-Rat IgG:Alexa 588 (anti-FlaB) and anti-Rabbit IgG:Alexa 647 (anti-GFP/anti-NapA) were used to detect primary antibodies. In all cases, images were acquired by phase-contrast microscopy (Ph), epifluorescence microscopy (middle two panels), and epifluorescence channels were merged (M). All scale bars = 5 μm. (F) Population level analysis of signal intensities from each treatment in C-E. Phase-contrast micrographs were used for automated cell detection, and total signal intensities, for each cell, were calculated and used to generate violin plots. No permeabilization (upper panel); outer membrane permeabilization (middle panel); outer/inner membrane permeabilization (lower panel) are shown and grouped by strain, and target. Each data set contained > 300 cells. All average signal intensities were statistically significant (unpaired *t-*test, *p* < 0.001) between upper panel and lower two panels, except for anti-NapA in 5A11/*napA* strain. (G) Demographs of NapA signal attained from outer membrane permeabilization (periplasmic signal, upper panel) and outer/inner membrane permeabilization (cytoplasmic signal, lower panel). Cells were organized by cell length, fluorescent intensity profiles were generated for each cell, and plotted as a heatmap (0–1).

Despite their apparent paucity [24,25], we hypothesized that *B*. *burgdorferi* does, indeed, produce PG-associated proteins (PAPs) that may be functionally akin to Braun’s lipoprotein (i.e., play a structural role in PG and cell-wall support), but are not easily identifiable using standard *in silico* homology searches. To test our hypothesis without any a priori assumptions, we purified *B*. *burgdorferi* PG using standard methods [16,33]. An initial purification step solubilizes most cellular components using 5% boiling sodium dodecyl sulfate (SDS). Following solubilization, PG as well as PG-associated material was collected. After washing to remove SDS, sacculi were treated with trypsin, which cleaves PAPs (Fig 1A). Intact PG was removed from liberated PAP peptides and fragments were identified by LC-MS (Fig 1A). Initial results yielded several candidate proteins but were contaminated with known cytoplasmic proteins (S1 Table). In follow up experiments we performed more rigorous sample processing, which included repeating the solubilization step with 5% boiling SDS. In these harsh sample preparation procedures only two hits met the following criteria: 1) were present in all five biological replicates; 2) consistently had MASCOT scores > 45; and 3) at least two unique peptides identified in each experiment. Using these exclusion criteria BB0690 (S1 and S2 Tables) was the top PAP candidate.

BB0690 has many names. Earlier studies identified BB0690 bioinformatically as a homologue of Dps (DNA binding protein from starved bacteria) and demonstrated that, like Dps, its production is further induced by stress [34–37]. Unlike Dps produced by most bacteria, BB0690 lacks the DNA-binding domain and does not bind DNA [34]. Curious, since the main function of Dps is to decorate DNA and protect heritable material from oxidative stress [37,38]. Subsequent studies demonstrated that BB0690 does play a role in oxidative stress by sequestering copper and iron metal ions, and thereby earned the name BicA (*B**orrelia* iron copper binding protein A) [39]. Perhaps the most well studied phenomena associated with BB0690 is its ability to attract neutrophils and modulate innate immune responses [38,40–42] which precipitated the alternative moniker NapA (Neutrophil attracting protein A). We contend that the basic function of BB0690 is not well understood. However, for simplicity, we refer to BB0690 as NapA for this communication.

### Sub-cellular localization of *B*. *burgdorferi* NapA

Despite the paradoxical role of NapA acting to protect *B*. *burgdorferi* against metal-stress [34,39,43], but yet does not bind DNA [34], the sub-cellular localization of NapA has never been determined. To begin, we first validated the specificity of polyclonal anti-NapA serum [35] raised in rabbits in two strains—a fully infectious derivative of the B31 type strain (5A11) and a mutant strain in which the *napA* locus has been replaced by a kanamycin resistance cassette *aphI1* (5A11/*napA*) [34]. Within the expected size range, anti-NapA yielded a single band in parent strain 5A11, which was absent in 5A11/*napA* (Fig 1B). Comparative, whole genome sequencing results of 5A11 and 5A11/*napA* indicated that the resistance cassette, *aphI1*, completely replaced the *napA* locus in the mutant, but no additional mutations were present (S3 and S4 Tables).

Upon strain and reagent validation, we probed different cellular compartments for NapA using a modified immunofluorescence technique. We reasoned that if NapA is directly associated with *B*. *burgdorferi* PG, then we would expect to detect NapA in the periplasm. To ensure that we were able to distinguish between possible periplasmic- from cytoplasmic-derived signal, we co-transformed both the wild type 5A11 and 5A11/*napA* strains with a plasmid that constitutively produces GFP [44]. We would expect that GFP would be exclusively localized to the cytoplasm. Our periplasmic control consisted of the abundant flagella filament protein FlaB [31,45]. Both 5A11 and 5A11/*napA* strains were cultured, fixed with paraformaldehyde, and treated with buffer (no permeabilization), 50% methanol (OM permeabilization) [46], or detergent and PG-degrading lysozyme (OM/IM permeabilization). Each permeabilization method was validated by immunofluorescence using anti-FlaB (periplasm) and anti-GFP (cytoplasm). Regardless of strain, untreated fixed cells yielded no detectable signal when probed with any antibody, indicating that our fixation method did not compromise either spirochete membrane (Fig 1C and 1F, upper panel). In contrast, methanol treatment permeabilized the OM, as previously reported [46], resulting in robust anti-FlaB signal (Fig 1D and 1F, middle panel). Importantly, OM permeabilization did not result in loss of IM integrity, as indicated by background levels of signal intensity when probed with anti-GFP (Fig 1D and 1F, middle panel). As expected for a PAP, NapA was readily detectable in the periplasm, but only in 5A11 wild-type cells (Fig 1D). Population level analysis of over 500 cells indicate that periplasmic NapA signal was intense, and comparable to the constitutively produced flagellar filament protein FlaB (Fig 1F, middle panel). Not until fixed cells were completely permeabilized, were we able to detect GFP with anti-GFP antibody (Fig 1E and 1F, lower panel). Under these conditions NapA can be readily detected as well, but demograph—single-cell, population-level signal intensity analysis, organized by cell-length—suggests cytoplasmic NapA signal is more sporadic than the uniform, periplasmic NapA signal (Fig 1G). We believe the latter could be attributed to the cytoplasmic permeabilization step, which also degrades some PG material with lysozyme treatment. Be that as it may, our limited permeabilization approach indicates that NapA originates in the cytoplasm but is readily detected in the *B*. *burgdorferi* periplasmic space.

### NapA is associated with the PG of *B*. *burgdorferi*

**Fig 2:**
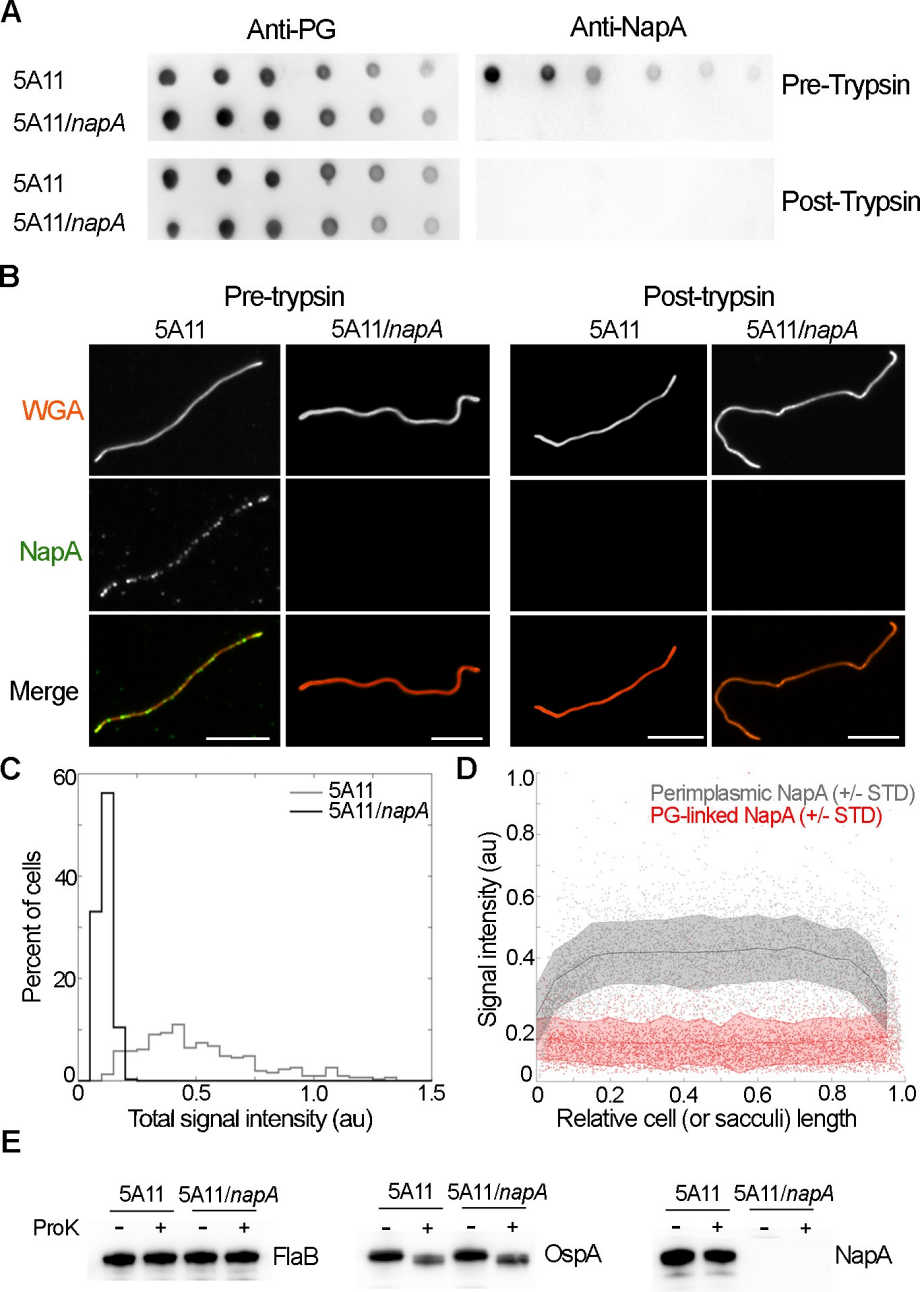
NapA is a PAP. (A) Dot blot analysis of PG. Wild-type (5A11) and *napA* mutant (5A11/*napA*) bacteria were cultured to mid-log exponential growth, cells were harvested, and PG was purified. Prior to trypsin treatment, one half of each sample was removed. Serial dilutions of each pre- and post-trypsin preparation were spotted on nitrocellulose and probed for PG (anti-PG, left) or NapA (anti-NapA, right). (B) The same sample preparation described in A were used for immunofluorescence studies. Whole PG sacculi were visualized by epifluorescence microscopy using wheat germ agglutinin (WGA, red) conjugated to Alexa Fluor 350. NapA (green) was detected using anti-NapA antibody and anti-rabbit IgG conjugated to Alexa Fluor 488. Scale bars = 5 μm. (C) Population-level analysis of integrated fluorescent signal intensities of NapA from sacculi isolated from 5A11 (n = 310) and 5A11/*napA* (n = 345). (D) Scatter-plot analysis of NapA signal in methanol treated, fixed cells (gray, n = 532 see Fig 1D) relative to NapA signal from purified PG (red, n = 310), pre-trypsin treatment. Scatter-plot shading corresponds to +/- 1 standard deviation (STD) while dark lines represent moving averages. (E) Proteinase K assay to determine protein surface exposure. Both 5A11 and 5A11/*napA* were cultured to identical densities, each split in half, gently harvested by centrifugation, and treated with (+) 5ug/mL of Proteinase K (ProK) or PBS diluent control (-). After 1 hour protease was inactivated and surface exposure of FlaB (left), OspA (middle), or NapA (right) was determined by western blot.

To determine if NapA is associated with PG, we developed a strategy based on the concept of our screen (Fig 1A). Parental and *napA* mutant strains were cultured to mid-log exponential growth, cellular components were solubilized with boiling SDS and insoluble material was collected. One half of the insoluble material was removed, and the remainder treated with trypsin, as is typical for PG purification. A dilution series of each sample was spotted on nitrocellulose and probed for NapA and PG. Dot blots demonstrated that PG was present in all samples tested (Fig 2A), indicating that each sample was processed similarly and contained relatively equal amounts of cell-wall material. Much like PG, NapA signal was clearly present, and reduced with each dilution, but only in material purified from parental cells, prior to trypsin treatment (Fig 2A). These data support the notion that, even after harsh treatment in boiling detergent, NapA is associated with *B*. *burgdorferi* PG. To further assess their relationship, we used immunofluorescence on separate biological replicate samples, prepared as described above. To circumvent anti-serum incompatibility issues (rabbit anti-PG/NapA), we used Wheat Germ Agglutinin (WGA) conjugated to Alexa-350 to detect *B*. *burgdorferi* PG. WGA is known to bind Glc*NA*c—a ubiquitous PG sugar. Consistent with dot blot results, PG sacculi pre- and post-trypsin treatment were clearly present in relatively equal abundance for both parental and *napA* mutant strains (Fig 2B). PG remained intact and WGA-PG derived signal was relatively uniform, with the possible exception of sacculi poles (Fig 2B), suggesting that neither sample preparation nor the presence of NapA impacted PG signal (Fig 2B). Prior to trypsin digestion, NapA appears to be scattered throughout the PG sacculus (Fig 2B). Population level analysis of NapA signal, normalized by total sacculi area, was greater than 5-fold above background (Fig 2C). While NapA signal appeared to display discrete patterning (Fig 2B), population level assessment of relative position indicated that NapA is approximately equally distributed throughout PG sacculi (Fig 2D). Overall NapA-PG signal, attained from purified sacculi, was about half that of periplasmic NapA signal (Fig 1D), with clear depletion at the poles (Fig 2D). The cause for the latter can be rationalized by the fact that intact cells have extended poles that are free of PG [33,45] and, by their association, NapA (Figs 1G and 2D). The former is likely due to harsh purification steps. Taken together, our immunoblotting and immunofluorescence studies confirm that NapA is a PAP in the Lyme disease spirochete.

Some PAPs traverse the OM and can be detected on the bacterial surface [20]. Such localization would be noteworthy for a protein thought to influence immune cell chemotaxis [40–42]. To determine if NapA was surface-exposed, we incubated live parental and *napA* mutant cells with Proteinase K, which would cleave surface-exposed proteins and alter their size by western blot. NapA was detected in wild type 5A11 cells, regardless of treatment, whereas surface-exposed OspA was readily cleaved and its mobility was clearly affected (Fig 2E). The same samples and treatments were probed with anti-FlaB to confirm OM integrity during protease incubation. All samples contains FlaB and migrated similarly, confirming the sample preparation quality. We conclude that NapA is located in the periplasm of *B*. *burgdorferi*, but is not surface-exposed.

### NapA provides structural and physiological integrity to the cell-wall

Since NapA is in the periplasm (Fig 1) and decorates the PG sacculus of *B*. *burgdorferi* (Fig 2), we speculated that NapA-mediated protection from exogenous stress may be at the level of cell envelope integrity. If cell envelope integrity is compromised in a NapA deficient bacterium, then any cell-wall stress should produce a phenotype, not just oxidative stress [35,39]. To evaluate this, we first incubated 5A11 and 5A11/*napA* cells with increasing amounts of NaCl and Lysozyme, which cause osmotic and PG specific stress, respectively. We monitored microtiter plates for changes in pH—an indirect measurement of growth [47,48]. Wild-type cells were more than 8 times more resistant to NaCl-induced stress (S1A Fig, right). Similarly, titrations of Lysozyme, which attacks the β 1–4 glycosidic linkage between PG glycan sugars Glc*NA*c and Mur*NA*c ablated 5A11/*napA* growth at 5–6 times less enzyme than the wild-type bacteria (S1A Fig, left).

**Fig 3:**
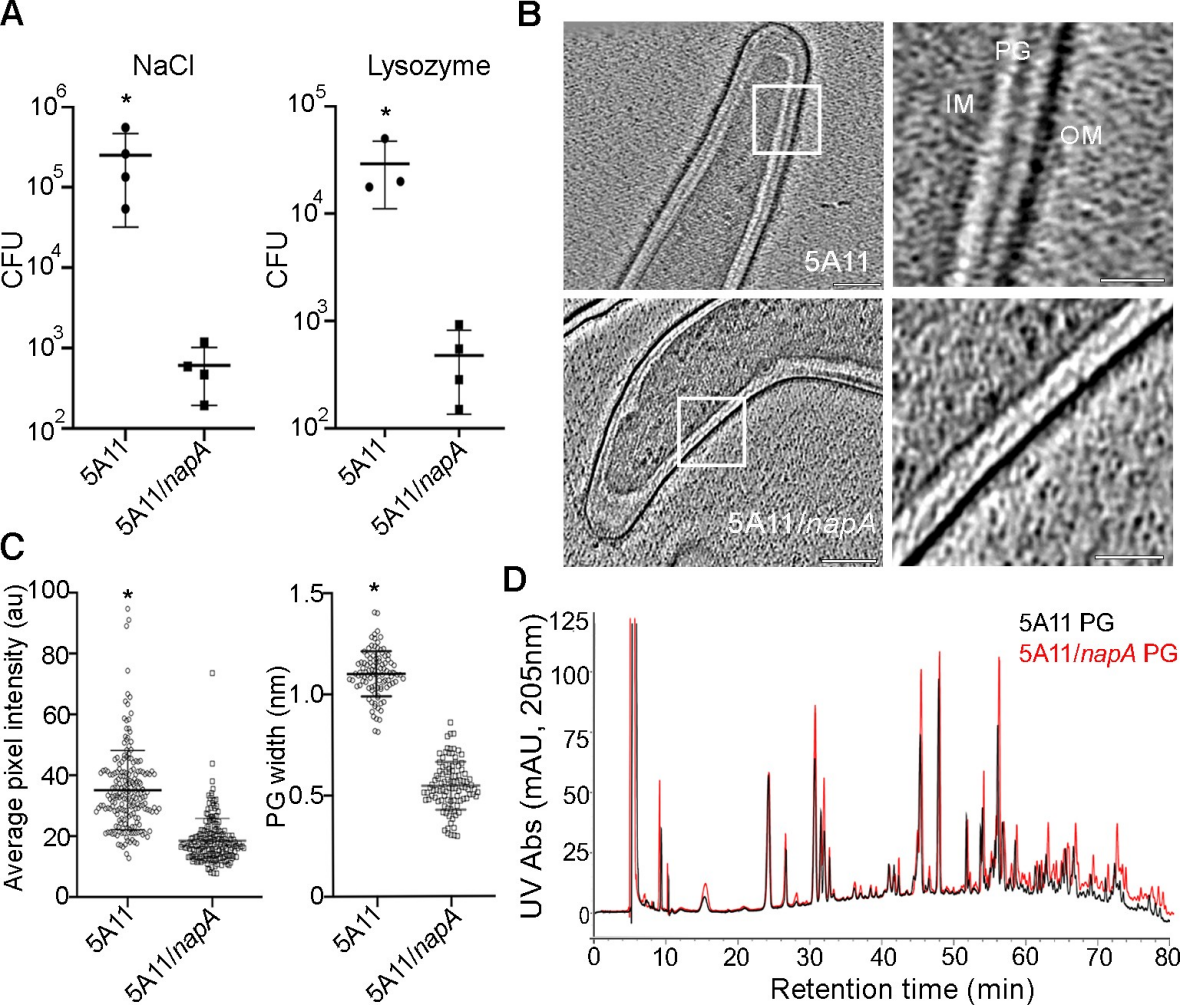
Cell envelope stress and defects of NapA deficient bacteria. (A) Osmotic and lysozyme susceptibility in wild-type (5A11) and *napA* mutant (5A11/*napA*) bacteria. Following exposure to 0.100 M NaCl (total osmolality 544 mOsm, left) or 0.375 mg/mL Lysozyme (right) for 18 hours, each strain was diluted in fresh media and plated. Three (wild-type) or six (mutant) weeks later, CFUs were determined. Bars shown are the mean +/- standard deviation from 4 experimental BSK II plates with either strain. P-value determined using unpaired t-test, * = *p* < 0.05. (B) Cryo-electron micrographs of the inner membrane (IM), peptidoglycan (PG) and outer membrane (OM) of the 5A11 (top) and 5A11/*napA* (bottom) strains. Scale bar 100 nm. (C) Population-level analysis of average PG width (right) and average PG pixel intensity (left) normalized by sampling area. Note that measurements excluded PG from 10% of each cell pole since these areas are thicker and more variable. (D) Liquid chromatography spectra attained from muropeptides, isolated from strain 5A11 (black) and 5A11/*napA* (red). Each strain was cultured, enumerated, and PG was purified for an equal number of bacteria. Following mutanolysin digestion, an equal amount of each sample was injected, and muropeptide abundance (UV absorbance) was plotted as a function of retention time.

Our data hint at a cell-wall defect in NapA deficient bacteria, however our microtiter plate assay also suggested a growth defect (S1A Fig). Indeed, direct culture enumeration indicated that NapA deficient bacteria replicate 1.9 times slower than the parental strain (S1B Fig). Since data presented in S1A Fig were end-point measurements after 6 days, we reasoned that the growth defect could account for the apparent differences in susceptibility to cell-wall stresses. To circumvent these issues, we performed stress tests on both strains at a single, previously optimized concentration of NaCl and Lysozyme (S1A Fig) for 18 hours in liquid broth. Each strain, and treatment, were then diluted in plating media lacking stress and colony forming units (CFUs) were determined. Wild-type CFUs were calculated after 3 weeks and, to compensate for growth defects (S1B Fig), compared to results obtained from the *napA* mutant bacteria after 6 weeks. Even after accounting for growth rate defects, mutant bacteria were 2–3 logs lower in CFUs (Fig 3A), indicating that NapA plays a basic role in cell envelope integrity and homeostasis. Our data are consistent with earlier studies that NapA provides protection from exogenous stress [34,35,39]. However, we surmise that NapA protects *B*. *burgdorferi* from all cell-wall stresses, potentially by reinforcing PG.

The distribution of NapA throughout the PG sacculi (Fig 2B and 2D) suggested that NapA may help bolster the PG, and that this association is essential to overall cell-wall integrity (Fig 3A). Mechanistic insights were provided by comparative Cryo-Electron Microscopy (Cryo-EM) analysis of *napA* mutant and parental strains. Bacteria unable to produce NapA possessed compromised PG—appearing discontinuous, thinner, and more ruffled—relative to the thicker, more electron dense PG layer of the parental strain (Fig 3B). Analysis of multiple Cryo-EM micrographs along the cell body from each strain demonstrated that both were true. The thickness of PG sacculi in mutant bacteria was, on average, roughly half (0.53) that of wild type cells (Fig 3C). Integrated average PG pixel intensity values, normalized by sampling area, were also significantly lower in the *napA* mutant strain (Fig 3C). It remained possible that the observed phenotype were the collective consequences of impairing PG biosynthesis. In other words, was NapA production in some way linked to PG synthesis, causing aberrations in cell-wall integrity? We addressed this possible scenario by purifying PG from the same number of cells, collected from both 5A11 and 5A11/*napA* strains. Pure PG was digested with mutanolysin, reduced, and separated by liquid chromatography. Comparative analysis of muropeptides profiles, attained from each strain, were nearly identical in terms of retention time and were negligibly different with respect to abundance (Fig 3D), indicating the NapA production does not influence PG synthesis. Instead, our data support the role of NapA in cell envelope integrity and provide insights into the mechanism(s) by which it protects the cell from stress.

### NapA and PG fragments are secreted in *B*. *burgdorferi* outer membrane vesicles

Bacterial elongation requires PG synthesis. Newly synthesized PG multimers are incorporated into the existing structure, resulting in expansion, but at a cost. Each incorporation event requires that incisions are made to provide substrates for transglycosylation reactions. Most diderms typically recycle excised PG monomers back into the cytoplasm for reuse. *B*. *burgdorferi* lacks the transporters and enzymes necessary for PG recycling. The result—approximately 45% of *B*. *burgdorferi* PG is shed per generation from the periplasm into the extracellular environment [16]. How these PG fragments cross the outer membrane boundaries of the cell envelope is not known.

**Fig 4:**
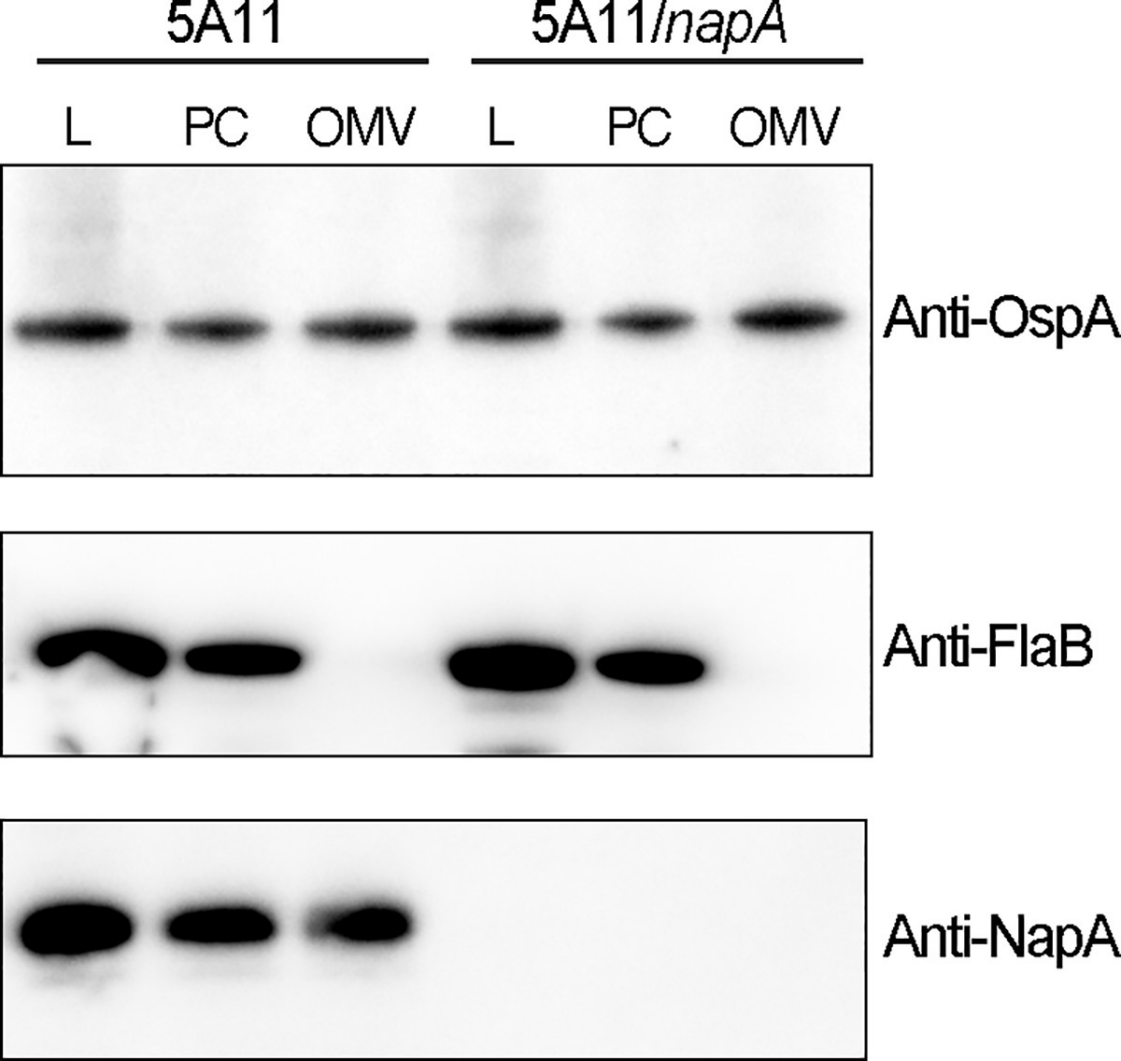
NapA is released in outer-membrane vesicles. (A) Wild-type (5A11) and *napA* mutant (5A11/*napA*) were cultured to mid-log (2.5 X 10^7^ cell/mL), cells were collected, washed, and processed into crude lysate (L), Outer membrane vesicles (OMVs), or protoplasmic cylinders (PC) as described in the methods. Each fraction was standardized by total amount of protein (Bradford assay) and assayed by immunoblot for OspA (anti-OspA, upper panel), FlaB (anti-FlaB, middle panel), or NapA (anti-NapA, lower panel).

We postulated that PG and, by extension, potentially NapA, could be released from the periplasm in Outer Membrane Vesicles (OMVs). *B*. *burgdorferi* produces OMVs, not only under stress, but also under regular homeostatic conditions [49], which support PG release [16]. To extend upon our findings and query OMVs for specific occupants, we prepared OMVs and protoplasmic cylinder (PC) fractions from wild-type and *napA* mutant bacteria as previously described [49] and compared our fractions (S2 Fig), by immunoblotting to a portion of our input (lysate, L). Fractions from each strain contained OspA (Fig 4), a well characterized OM protein known to be released in OMVs [49,50]. In contrast, FlaB—an abundant PC protein—was undetectable in our OMV preparations (Fig 4). Probing each fraction for NapA yielded similar results to OspA, indicating that the full-length protein was indeed in *B*. *burgdorferi* OMVs isolated from wild type cells (Fig 4).

**Fig 5:**
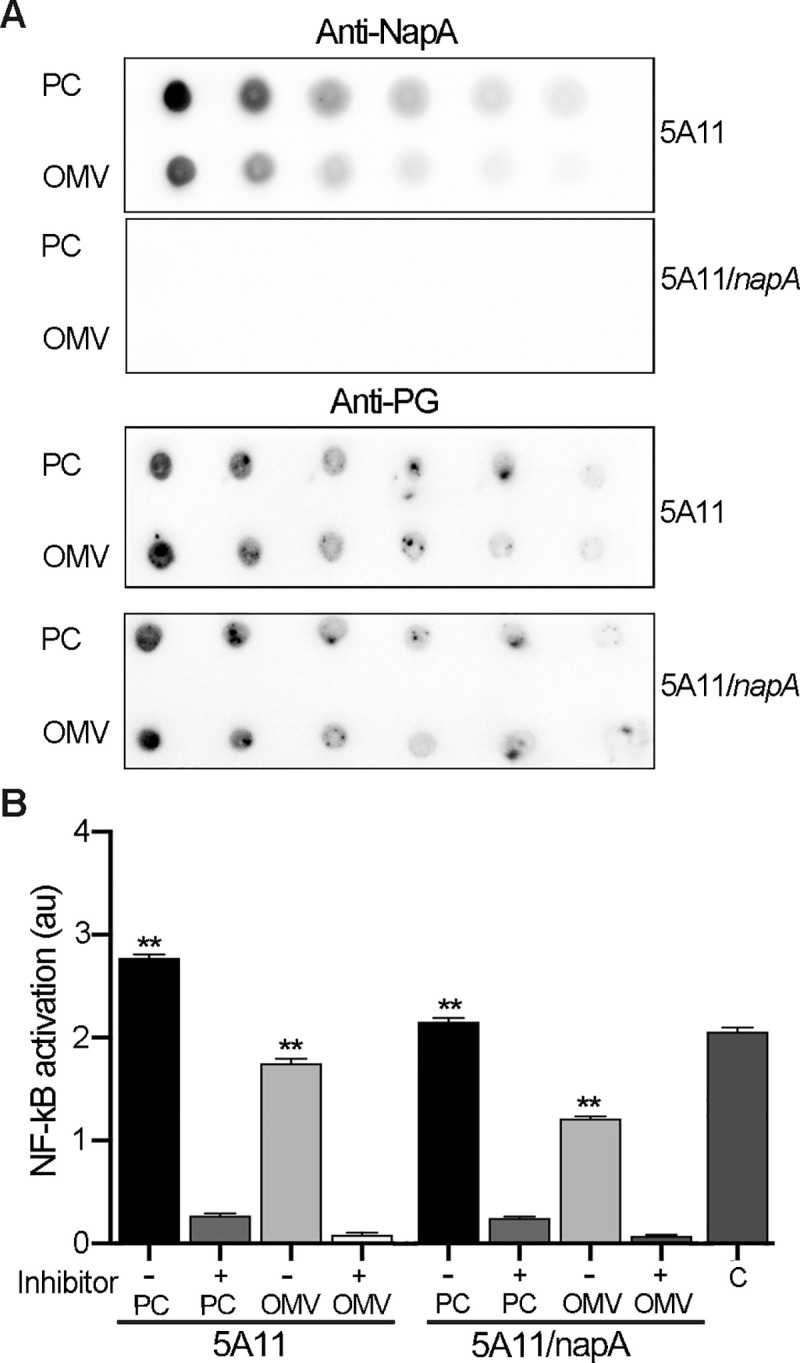
NapA-PG is released in outer-membrane vesicles. (A) The same OMV and PC fractions analyzed in Fig 4 were serially diluted and assayed for NapA and PG by dot blot. (B) Reporter assay to query PC and OMV fractions for PG containing Muramyl dipeptide (MDP). Human NOD2 reporter cell line (hNOD2, Invivogen) was used to estimate the relative amount of MDP in sample with and without 20ug/mL of gefitinib—an inhibitor of the effector downstream of hNOD2, RIP2. MDP (50 pg/mL) served as the positive control (C) reactions. Bars shown are the mean of samples tested in triplicate, +/- standard deviation. ** = *p* < 0.001, unpaired *t-*test, with and without inhibitor.

Given the NapA-PG association, we reasoned that released muropeptides and/or fragments of polymeric PG may also be included in OMVs. Given the large distribution of potential PG sizes, we opted for dot blot analysis of each fraction and co-immunoblotting with anti-PG and anti-NapA. PG could be detected in the OMV fraction in both parental and *napA* mutant preparations (Fig 5A), indicating that NapA is not required for PG to be present in OMVs. We further confirmed that *B*. *burgdorferi* OMVs contain PG by a ligand-receptor reporter assay. PC and OMV fractions were incubated with a hNOD2 receptor reporter cell line, which, when exposed to PG containing Muramyl-L-Alanine-D-Glutamine (MDP), activates the secretion of alkaline phosphatase. To control for non-specific activation, we included the inhibitor gefitinib, which acts on the adaptor protein RIP2, downstream of NOD2 signaling [16,51]. Both fractions from each strain resulted in significant hNOD2 activation (Fig 5B), which was reduced 5-6-fold when inhibitor was added (Fig 5B). We note that OMV contents were capable of activating a cytoplasmic receptor which indicates that 1) *B*. *burgdorferi* OMVs lysed during the experiment; 2) uptake occurs via phagocytosis; or 3) they are capable of fusing with eukaryotic membranes and expelling their contents, as reported for other bacteria [28,52].

### NapA-associated PG acts as a molecular beacon, augmenting the immunomodulatory properties of the *B*. *burgdorferi* cell wall

**Fig 6:**
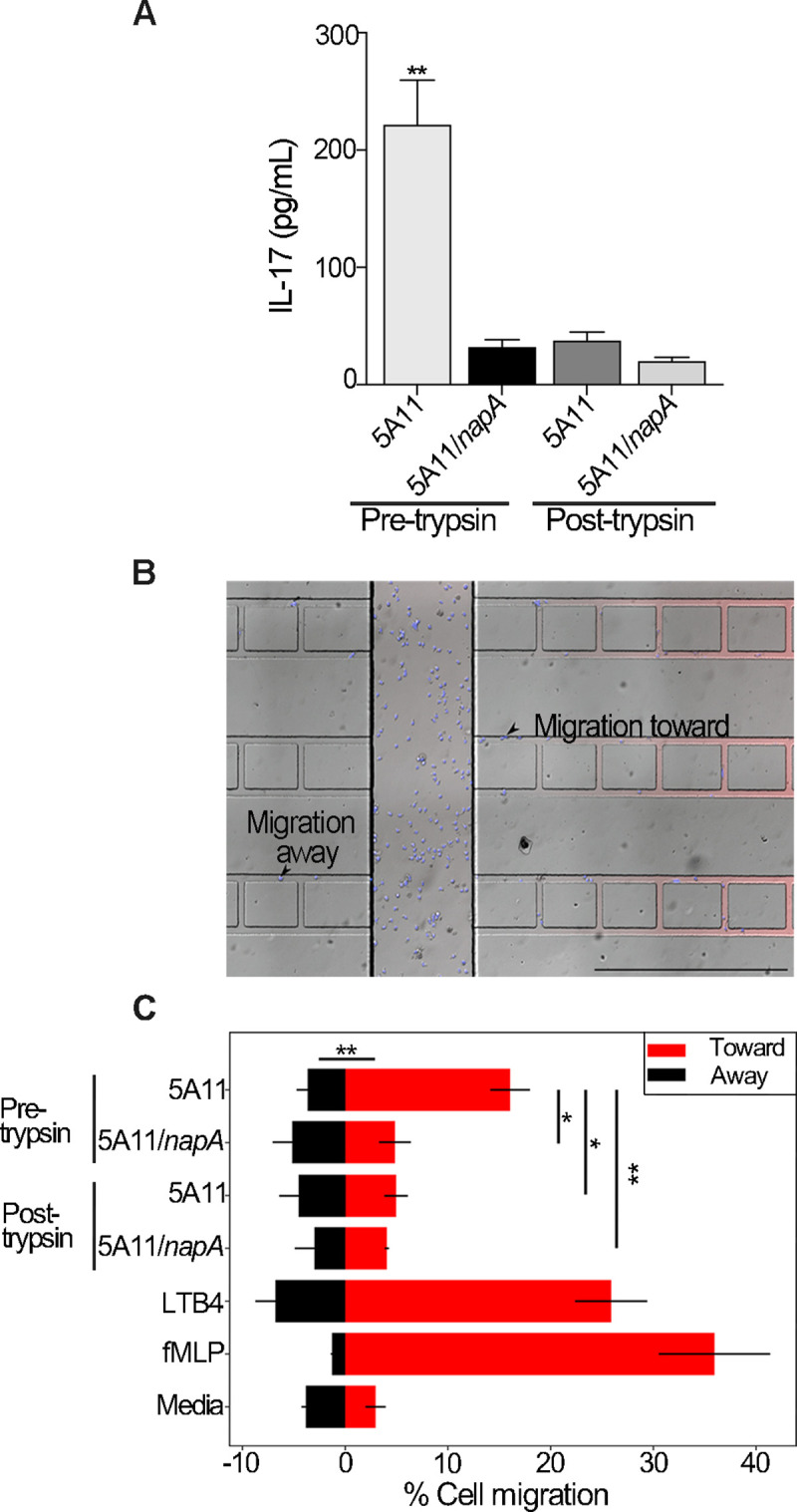
NapA stimulates IL-17 and induces neutrophil migration. (A) IL-17 production by human peripheral blood mononuclear cells (PBMCs). Three pools of eight mixed donor PBMCs samples were stimulated with 10 ug/mL of PG, before and after trypsin treatment, from wild-type and *napA* mutant bacteria. Culture supernatants, from each stimulation, were assayed for IL-17 by ELISA (Abcam). Values are the mean, +/- standard deviation, after normalizing for untreated, PBS diluent control are shown. Statistical analysis unpaired *t*-test, * = *p* < 0.005. (B) Merged Phase-contract/epifluorescence micrograph of microfluidic competitive chemotaxis-chip (μC^3^) (55) used to measure dHL-60 cell (blue) migration both toward (red) and away (black) from gradients of each stimulus. Scale bar = 500 μm. (C) dHL-60 cells show a higher percentage of cells migrating toward PG-linked NapA. Reservoirs that flank each maze were loaded with 125 μg/mL of each PG sample, diluted in dHL-60 cell culture media, and compared to opposite reservoir, which contained culture media alone. Controls included media, 10nM of Formylmethionine-leucyl-phenylalanine (fMLP), and 100nM of Leukotriene B4 (LTB4). Data were collected over 5 hours, images captured every 2 minutes, while cells were maintained at 37°C under 5% CO_2_. Results shown are mean +/- SD of three biological replicate experiments. To evaluate differences between responses ANOVA were performed with Turkey’s correction for multiple comparisons (* = *p* < 0.05, ** = *p* < 0.005).

*B*. *burgdorferi* PG was recently shown to be a persistent antigen in the synovium of LA patients and is capable of inducing both inflammation and arthritis [16]. These studies were performed using purified *B*. *burgdorferi* PG, which includes a trypsin digestion step to cleave any linked proteins. Since PG is associated with NapA in its natural biological state, we questioned whether the combination may augment the inflammatory response. Here, we focused on IL-17 since 1) *B*. *burgdorferi* PG only modestly increased IL-17 secretion [16]; 2) IL-17 is markedly over-represented in LA patients [16,53,54]; and 3) previous studies have found that recombinant NapA can stimulate an T_H_1/T_H_17 response [40,42]. Using OMVs containing NapA-PG is complicated by package contents and casing. Instead, we used the same pre- and post-trypsin treated PG samples as above, prepared from wild-type and mutant *napA* bacteria. Relative to *napA* mutant derived PG preparations, wild-type PG caused human PBMCs to secrete ~9-fold more IL-17 (Fig 6A), which highlights two important points: 1) The NapA-PG association has immunological consequences and 2) while it is possible that other proteins are associated with *B*. *burgdorferi* PG (S1 and S2 Tables), NapA alone seems sufficient to augment the PG-induced IL-17 response (Fig 6A).

Since NapA-PG produced higher levels of IL-17, it may also act as a molecular beacon for neutrophils, naturally. To determine the chemoattractant capabilities of NapA-PG, we performed a comparative study using real-time neutrophil tracking in a microfluidics chamber. In this system, neutrophils flow into a central chamber that is flanked by reservoirs on each side (Fig 6B). Migratory bait is added to one reservoir and compared to the adjacent media-containing reservoir. Migration towards a potential stimulus was monitored by phase-contrast and epifluorescent microscopy for 5 hours. Percent migration was determined by the number of cells that reached a flanking reservoir. The only PG bait that acted as a significant chemoattractant was NapA-associated PG (16.03 ± 1.93%) (Fig 6C and S1–S7 Movies); similar to that of known attractants LTB_4_ (25.89 ± 3.52%) and fMLP (35.94 ± 5.42%) [55]. None of the other preparations caused significant attraction or repulsion (Fig 6C). Cells migrating toward NapA-associated PG also showed more directional migration—less cells migrated within cell mazes and displayed oscillatory migration (S5 Table and S3 Fig). Moreover, cells migrated toward NapA-associated PG with higher velocity (10.94 ± 4.79 μm/min), relative to cells migrating toward other preparations (S5 Table and S4 Fig). Since data presented are the combined results of three biological replicates, and the only difference between pre-trypsin treated 5A11 PG and pre-trypsin treated *napA/*5A11 PG is the presence of NapA, our data suggest that NapA is both necessary and sufficient to cause neutrophil migration toward *B*. *burgdorferi* PG.

## Discussion

Our studies provide evidence for a PAP in *B*. *burgdorferi*. We report that NapA exists in the periplasm but is not surface exposed (Figs 1 and 2). Molecular and cellular studies demonstrate a NapA-PG interaction, and that this association is important in stabilizing the *B*. *burgdorferi* cell envelope (Figs 2 and 3). NapA-PG is not only important for physical and physiological homeostasis, but also the nature of the interaction has pathogenic consequences resulting in increased IL-17 production and neutrophil attraction (Fig 6). Here we discuss our findings in the context of spirochete biology, Lyme disease, pathogenesis, and bacterial evolution.

Classical PAPs, produced by most bacteria, play a basic physiological role in cell envelope homeostasis. These proteins are often abundant and function to situate the PG layer at an appropriate distance from the IM and/or OM, often through lipidation. In this sense, NapA is atypical in that structural [41] and in silico analysis of the N-terminal region lack evidence for a lipidation site [29,56,57]. Instead of acting as a structuring scaffold to maintain PG position within the periplasm, we favor an alternative mode of cell envelope protection whereby NapA decorates the PG (Fig 2), provides continuity during turnover (Fig 3), and both sequesters reactive species [34–36,39] and other exogeneous stress (Fig 3). Recent studies in pathogenic Leptospira discovered a novel PG binding lipoprotein LipL21, which functions to bolster the PG while also acting to protect the cell from NOD1 and NOD2 detection [58], highlighting the dual function of seemingly pure structural proteins [17,20,23].

The natural life cycle of *B*. *burgdorferi* is complex and involves establishing residency in very different hosts, including the tick vector and dozens of potential vertebrate hosts [26]. Earlier studies have shown that *napA* is dispensable for mouse infection but required for tick survival [34]. Bacteria unable to produce NapA are more susceptible to PG-specific stress (Fig 3). With the exception of host blood, it is not clear what stressors would be present in the tick mid-gut or how NapA ameliorates the osmoprotective properties of PG. *Ixodes scapularis*, however, does produce the *B*. *burgdorferi* PG-specific hydrolyzing enzyme Dae2 [59,60] in addition to lysozyme [61], which could function more effectively in the absence of NapA-linked PG (Fig 3).

Clearly, much remains to be determined in lieu of our findings. For instance, the nature of the NapA-PG association is not known. PAPs bind their PG substrate through covalent and non-covalent interactions [62]. While we cannot exclude either possibility, we observe less NapA signal in purified PG relative to periplasmic-derived NapA signal (Fig 2D); the latter is boiled for hours in 5% SDS. This suggests non-covalent interaction(s). Furthermore, *B*. *burgdorferi* NapA lacks the classical export signal sequence consistent with Sec-mediated secretion [29,57]. Others have found instances in which proteins are secreted from the cytoplasm through unknown mechanism(s), both in *B*. *burgdorferi* [63–66] and in many other bacterial phyla [67]. We speculate that this could occur in conjunction with flagellum assembly through the dedicated Type 3 Secretion System, a system that lacks a consensus signal sequence [68] and is capable of exporting non-flagellar components associated with cell envelope homeostasis and virulence [69–73]. Alternatively, a yet to be defined system that is functionally analogous to the twin arginine transporter [74], which secretes folded proteins [75], but recognizes a different signal sequence, could be present in the *B*. *burgdorferi* genome, although not easily identified using standard bioinformatics. Far too often bioinformatics have failed to correctly assign seemingly conserved biological function to hypothetical proteins in this unusual genus [76–78]. These anomalies notwithstanding, NapA is readily detected in the periplasm (Fig 1), associated with PG (Fig 2 and S1 and S2 Tables), and abundant in OMVs (Figs 4 and 5). We note that others have corroborated the latter (personal communication, Wolfram Zückert).

Cell elongation requires both PG anabolism and catabolism. Excised muropeptides accumulate outside the cell and are involved in the pathogenesis of LA [16]. Until now, there has been no mechanism to explain how released PG crosses the *B*. *burgdorferi* OM. Here, we show that one route of PG release is through OMVs (Fig 5). Based on our cellular reporter assay, OMV contents can end up inside eukaryotic cells (Fig 5B). Several mechanisms have been proposed, including endocytosis and membrane fusion [52]. Two-way lipid exchange has been shown to occur following the internalization of *B*. *burgdorferi* OMVs [28], which supports the notion that OMVs may also be used for exchange of periplasmic contents such as NapA and other potentially pathogenic material. Of course, it is also possible the OMVs lyse, spilling their contents into the extracellular space of host systems. Regardless of the possible mechanism, NapA-linked PG augments the helper T cell response caused by PG alone, inducing higher levels of IL-17 (Fig 6A). These findings are in line with studies using rNapA, which has been implicated in LA [40,42]. While other PAPs are likely (S2 Table), these effects can likely be attributed to NapA-PG.

Neutrophils are akin to a platoon on the front lines—controlling the environment, initiating a response, and recruiting backup. During the initial stages of infection, neutrophils are recruited to the site of the tick bite; phagocytize *B*. *burgdorferi*; utilize lethal enzymes; and destroy bacterial cells using neutrophil extracellular traps (NETs) [79–81]. This initial attraction may be due to NapA-linked PG released from *B*. *burgdorferi* during growth or death at the site of inoculation. The latter may be a diversionary tactic whereby healthy bacteria are able to disperse to other parts of the body and cause more severe symptomology. In these later stages several different organs systems are involved, including the joints, heart, and central nervous system [9]. *B*. *burgdorferi* PG lingers in humans suffering from Lyme arthritis and trypsin treated PG can induce arthritis in the mouse model [16]. Therefore, the coordinated effect of both NapA and PG within the synovial tissue during later stages could exacerbate arthritis severity through the chemotactic properties of NapA-PG (Fig 6C). Taken together, our findings implicate that a structural protein moonlights as a molecular beacon for immune cells and attracts them to an abundant inflammatory molecule.

Dps homologues are produced by virtually all bacteria [82]. NapA shares structural and amino acid sequence similarities to some of the well-studied Dps homologues [37,41] (S5 Fig). There are, however, notable differences which may extend to other proteins which may have evolved to perform altered functions. 1) Dps production is highly upregulated in stressed conditions, and 2) acts by shielding DNA for oxidative damage, a ubiquitous function that appears to be highly conserved across diverse taxa [36]. *B*. *burgdorferi* NapA, on the other hand, does not bind DNA [34] and its role in cellular homeostasis is in the periplasm (Figs 1–3). NapA production does appear to increase under oxidative stress [35], but others have argued that basal production is considerable in culture ([34] and Fig 1D and 1F) and increased NapA expression by metal stress is negligible [83]. Our findings are in line with the latter—we detect considerable NapA under exponential growth (Fig 1), which would make sense for a structural protein. At the amino acid level, *B*. *burgdorferi* NapA has two distinct features that separate it from other Dps homologues. First, much like *H*. *pylori*, *B*. *burgdorferi* NapA has a truncated N-terminus that lacks the Lysine-rich residues implicated in DNA binding [34,84,85]. Unlike *H*. *pylori* NapA, the *B*. *burgdorferi* homologue has an extended C-terminus, which appears to be a unique to *Borreliae* (S5B and S5C Fig). The biological consequences of these differences remains to be determined. Regardless of the differences between Dps homologues, our findings highlight the ingenuity of bacteria in which a protein can change from a mechanistic standpoint, while maintaining the same basic biological function of protecting the cell from exogenous stress.

## Materials and methods

### Bacterial strains, eukaryotic cells, and growth conditions

Both *B*. *burgdorferi* strains used in this study were generously provided by Frank Gheradini (NIH). A laboratory clone of the *B*. *burgdorferi* B31 type strain, termed 5A11 [86] served as the wild-type parental control. The *napA* mutant was produced in the same 5A11 background and was created by allelic replacement as described previously [34]. To produce low-level, constitutive GFP expressing *napA* and 5A11 strains, we first created a promoter fusion construct between *bb0826* and monomeric super-folder *gfp* fusion construct [44] using compatible SacI and BamHI. The resulting plasmid (pBLJ516) was transformed [87] into each strain, and clones were selected by micro-plate dilution with gentamicin (40ug/mL), followed by fluorescence microscopy screening (see below). All *B*. *burgdorferi* cultures were propagated and maintained in Barbour-Stoenner-Kelly II (BSK-II) medium containing 6% rabbit serum at 37°C under 5% CO_2_.

Fresh, mixed donor human peripheral blood mononuclear cells (PBMCs) (Zen-Bio) were re-suspended in PBMC media (Zen-Bio) overnight prior to stimulations. Human NOD2 reporter cells, which were used to detect PG in OMVs (see below), were purchased from Invivogen and cultured as recommended by the manufacturer. Human promyelocytic leukemia cells (HL-60 CCL-240, American Type Culture Collection ATCC, Manassas, VA) were cultured in complete media comprising of Iscove’s Modified Dulbecco’s Medium (IMDM, ATCC, Manassas, VA) supplemented with 10% fetal bovine serum (FBS, ATCC, Manassas, VA) at 37°C in 5% CO2, according to ATCC instructions. HL-60 cells were differentiated into a neutrophil-like state with 1.5% dimethyl sulfoxide (DMSO, Sigma-Aldrich, St. Louis, MO) to 1.5×10^5^ cells/mL for five days. Differentiated HL-60 cells (dHL-60 cells) were stained with Hoechst solution [20 mM] for 10 minutes (Thermo Fisher Scientific, Waltham, MA) at 37°C and 5% CO2 and spun down and re-suspended into a concentration of 5.0×10^7^ cells/mL immediately before use in the migration assay.

### *B*. *burgdorferi* DNA purification and genome sequencing

Low-passage, 40 mL cultures of each strain, were propagated to late-log (10^8^ cells/mL) and harvested. After washing bacterial pellets three times with PBS, cells were lysed by sonication and DNA was extracted by standard phenol:chloroform methods. Crude DNA extracts were then purified using Zymo Research (Irvine, California) genomic DNA purification kit.

Whole genomic sequencing was performed by Microbial Genome Sequencing Center (MiGS, Pittsburgh, Pennsylvania), who provided >450X coverage for each sample. Unicycler was used to process and assemble all sequence data. Results were compared to the published type strain [24] and parent of the wild type derivative (5A11) used in these studies.

### Peptidoglycan purification and analysis

*B*. *burgdorferi* PG was purified as described previously [16] but was typically from 1L of culture. Briefly, cells were harvested at 3,500 x g, washed three times with PBS, and resuspended in PBS. Cell suspensions were added dropwise to 10% of boiling SDS. The final concentration of SDS 5%. After boiling for 1-hour, insoluble material was collected by ultra-centrifugation at 275,000 x g for 1 hour and washed five times with 20mL of 35°C water. PG was resuspended in PBS and treated with 1000 U Benzonase Nuclease (Sigma-Aldrich) for 4 hours at 37°C, followed by overnight digestion with 300 μg/mL chymotrypsin (Sigma-Aldrich) at the same temperature. SDS (1%, final concentration) was added to each and boiled briefly. Insoluble material was once again harvested as described above and washed 3 times. For comparative studies that included PAPs, samples were processed the same as above, but split such that only half of the material was treated with 300 μg/mL chymotrypsin. In immunological and chemoattractant studies, after processing as described above, each sample was digested with Mutanolysin (10,000 U/mL) at 37°C overnight. After removing undigested material by centrifugation, supernatants containing muropeptides were passed through a YM-10 filter and flow through dried. The concentration of the purified *B*. *burgdorferi* PG was determined using dry weight. *B*. *burgdorferi* PG was re-suspended in phosphate buffered saline (Thermo-Fisher) prior to use. We note that for all experiments in which PG preparations were treated with trypsin for PAP validation (Figs 2, 5 and 6) chymotrypsin was used, but for simplicity we refer to digestions in figures a ‘trypsin + or trypsin treated’.

For muropeptide analysis bacterial density was determined from 500 mL cultures and standardized such that PG was purified from an equal number of cells (2.5 X 10^10^ cells). PG purification and mutanolysin digestion occurred as described above. After centrifugation (21,000 x g for 30 minutes) to remove undigested material, supernatants were lyophilized. The resulting muropeptides were reduced with borohydride and analyzed as described previously [16].

### Identification of PAPs

To identify protein(s) associated with *B*. *burgdorferi* PG we performed the same crude extraction procedure described above, with the exception that insoluble PG sacculi were treated with Mass Spectroscopy grade Trypsin (Sigma-Aldrich), instead of chymotrypsin. Peptides released by protease treatments were desalted utilizing 100 μl C18 Bond Elut OMIX (Agilent) SPE tips following the manufacturer’s recommended protocol. Eluents were concentrated to dryness using a centrifugal vacuum concentrator. Peptides were reconstituted in 40 μl solvent A (98:2 LC-MS grade water: LC-MS grade acetonitrile supplemented with 0.1% (v/v) formic acid) by sonication. LC-MS grade solvents were obtained from Fisher Scientific. Aliquots (10 μL) were analyzed by liquid chromatography tandem mass spectrometry in data-dependent, positive ion mode, using an Orbitrap Fusion Lumos coupled to an Easy nLC1200 UPLC/autosampler (Thermo Scientific). Sample was loaded onto an C18 EASY-Spray HPLC analytical column (50 μm ID x 15 cm, 2 μm particle size with 100 and 0.1 nm pore size, Thermo Scientific), and peptides were eluted from the system at a flow rate of 300 nl/min with a 110-minute gradient from 98% solvent A to 55% solvent A. Solvent B was 20:80 LC-MS grade water: LC-MS grade acetonitrile supplemented with 0.1% (v/v) formic acid. The analytical column was maintained at 55°C and the ion transfer tube at 275°C. Electrospray voltage was set to 3000 V and the RF lens set to 30%. The MS^1^ scan utilized the orbitrap set to 120,000 resolution (*m/z* 200) over the *m/z* range of 500 to 1000 with an AGC target of 4e5, a maximum injection time of 50 msec in profile positive ion mode. Peaks exhibiting an isotopic envelope resembling a peptide with a charge of +2 to +5 and an intensity of at least 2e4 were subjected to MS^2^. MS^2^ utilized quadrupole isolation of ±1.4 Da, the orbitrap detector set to 15,000 resolution (*m/z* 200) and stepped HCD of 29–31% with the first mass of the MS^2^ scan set to 150 and a default charge state of +3. The AGC target was 1e5 with a maximum injection time of 200 msec in centroid positive ion mode. Dynamic exclusion prevented MS^2^ on the same peak for 15 seconds. Peptides were identified using Proteome Discoverer 2.2 (Thermo Scientific) using both Sequest HT and Mascot search engines. The data was searched against the *B*. *burgdorferi* reference proteome downloaded from UniProt and concatenated with a database containing common lab contaminant proteins. All peptides for trypsin digests were expected to be fully-specific for trypsin digestion with the possibility of up to two missed cleavages. All peptides for chymotrypsin digests were expected to be fully-specific for trypsin digestion with the possibility of up to three missed cleavages. MS^1^ tolerance was set to ± 10 ppm and MS^2^ tolerance was set to ± 0.1 Da. Oxidation of methionine, deamidation of asparagine and glutamine, acetylation of the protein *N*-terminus and formation of pyroglutamate from glutamine when at the *N*-terminus of a peptide were set as variable modifications.

In secondary experiments, we performed an additional solubilization step, prior to LC-MS identification of released peptides. Briefly, after solubilizing cellular material with 5% boiling SDS for 1 hour, PG was harvested by centrifugation, as described above. After washing 3 times with 20mL of ~50°C ultra-pure water, PG was re-extracted with 5% boiling SDS for an additional hour and allowed to cool to room temperature overnight. The next day, material was re-heated to 80°C for 30 minutes, centrifugation and washed, as described above, prior to peptide identification.

### Cellular fractionation

Periplasmic fractions from *napA/*5A11 and 5A11 parental cultures were isolated essentially as previously described [49]. Briefly, one liter of each strain was cultured in BSKII supplemented with 6% rabbit serum to a final density of 2.5 x 10^7^ cells/mL. To create a crude cell lysate, 40mL of each culture was separated from the 1-liter bulk culture and processed separately. Bacteria were harvested at 3,500 x g for 20 minutes and washed three times with PBS containing 0.1% BSA. The resulting crude cell lysate pellets were stored at -80°C. The other pellets, collected from ~960mL of culture, were resuspended in 120 mL of cold, 25mM citrate buffer (pH 3.2) and incubated with gentle shaking for 2 h. Every 20–30 mins, each sample vigorously vortexed for ~30 s. Both OMVs and PCs were collected by centrifugation at 21,000 x g for 20 mins, resuspended in 25mM citrate buffer, each sample split into 3 equal volumes, and applied to a discontinuous sucrose gradient (56%; 42%; 25%). All 6 tubes were centrifuged at 102,500 x g for ~ 18 hours at 4°C. OMV and PC fractions, from each sample, were remove by needle aspiration, pooled and collected by centrifugation at 142,000 x g for 6 hours. Each fraction, from each sample, were resuspended in 25mM citrate buffer and applied to continuous sucrose (10–42%) and separated as described above. Both PC and OMV were collected, once again, by needle aspiration, diluted in PBS, and collected by centrifugation at 12,500 x g for 20 mins, and 142,000 x g for 6 hours, respectively. The resulting material was resuspended in PBS, aliquoted, and stored at -80°C.

### Immunoblots

All antibodies used in this study have been previously characterized. Anti-FlaB [88] loading control, and anti-NapA [35] were graciously provided by Melissa Caimano and Frank Gheradini, respectively. Rabbit anti-serum raised against rOspA was purchased from Rockland Inc. Polyclonal anti-PG rabbit serum was recently validated and provided by the Christine Jacobs-Wagner lab. Dilutions for western blots were as follows: Anti-FlaB (1:1000); anti-NapA (1:8000); anti-OspA (1:1000); anti-PG (1:90). A 1:8000 dilution of rabbit IgG:HRP (Jackson labs) was used to detect all primary antibodies, with the exception of FlaB, which was detected with rat IgG:HRP (Jackson labs), used at the same dilution. All secondary antibodies were detected by chemiluminescence using SuperSignal West Pico PLUS (Thermo Scientific) detection reagents and imaged with a Syngene G:box (Imgene Technologies).

### Immunofluorescence

5A11 and *napA/*5A11 were cultured (40 mL) to a final density of 5 X 10^7^ cells/mL. Cells were fixed by quickly adding a freshly opened ampule of paraformaldehyde to final concentration of 2% (vol/vol). Cells were fixed, with gentle agitation for 10 minutes at room temperature, and the reaction terminated on ice for 30 minutes. After harvesting and washing the fixed cells, they were stored at -20°C.

Fixed cells were spotted on poly-L-lysine-coated slides. After washing with PBS supplemented with 0.05% Tween 20 (PBS-T) to remove unbound material, we proceed with different methods to permeabilize the cells. *No permeabilization* consisted of three PBS washes, and three Seablock (Abcam) prior to block the cells with Seablock for 2 hours. *Inner membrane permeabilization* involved treating cells for 10 minutes, at room temperature, with 50% methanol. Methanol was removed by aspiration, cells washed three times with PBS, then washed three times with Seablock prior to blocking for 2 hours with Seablock. For *Inner/outer membrane permeabilization* cells were first treated with 0.03% SDS for 3 minutes at 37°C. SDS was removed with PBS washes, and fixed samples were subsequently treated with 1mg/mL of lysozyme (Sigma-Aldrich). Permeabilized samples washed three times with PBS, then washed three times with Seablock (Abcam) prior to blocking for 2 hours with Seablock. Immunolabeling each target were the same for each sample and treatment. All antibodies were diluted in Seablock. Primary: secondary antibody pairs and dilutions were as follows: 1) GFP: mouse α-GFP, [1:100] (Sigma Aldrich); Goat α-mouse IgG:Alexa Fluor 647 [1:250] (Jackson Laboratories). 2) FlaB: rat α-FlaB, [1:75]; Goat α-rat IgG:Alexa Fluor 555 [1:250] (ThermoFisher). 3) NapA: rabbit α-NapA [1:400]; Donkey α-Rabbit IgG:Alexa Fluor 647 [1:250] (Jackson Laboratories). Primary incubations occurred at room temperature for 1-hour, unbound material was washed 15 times with PBS-T, and probed with secondary antibodies for 1 hour. Unbound secondary antibodies were washed 15 times. Slides were treated with SlowFade (ThermoFisher) and imaged as described below.

The immunofluorescence procedure for purified PG and PG-linked NapA were identical to that previously described [33]. Briefly, pre- and post-trypsin treated PG preparations were spotted onto poly-L-lysine-coated slides. After washing with PBS supplemented with 0.05% Tween 20 (PBS-T) to remove unbound material, samples were blocked with Seablock (Abcam) for 2 hours. Anti-NapA (1:400) was co-incubated with 5 μg/mL WGA:Alexa 350 on each sample for 1 hour and washed 15 times with PBS-T. Anti-NapA was detected with the Goat α-rabbit IgG:Cy3 conjugated antibody (Jackson laboratories), diluted 1:250. Control reactions included secondary antibody, without primary. Samples were treated with SlowFade and imaged as described below.

### PBMC stimulations

Pooled cryo-preserved PBMCs were seeded in 12-well plates at 2 x 10^6^ cells/well in Lymphocyte culture media (Zen-Bio), pre-equilibrated to 37°C under 5% CO_2_. Cells rested for 18 h under these conditions prior to stimulation. Following stimulation for ~72 hours with 25 μg/mL of *B*. *burgdorferi* PG, the cells were harvested by centrifugation at 800 x g for 5 min at 15°C. The supernatants were collected, aliquoted, and kept at -80°C prior to cytokine analysis. Cytokine analyses were performed on stimulated PBMC supernatants, diluted 1:4, according to the manufacturers (Abcam ELISA Kit IL-17A/F). Results were normalized to controls stimulation with diluent (PBS control). Statistical significance was determined by an unpaired Student’s T Test. Unless stated in the text, statistical significance was set at *p* <0.05.

### Microscopy and image acquisition

Samples were immobilized by poly-L-lysine-coated slides. Epifluorescence microscopy was performed on a Zeiss Axio Observer equipped with a Hamamatsu Orca-Flash 4.0 V3 Digital CMOS camera, Colibri 7, and an oil-immersion phase-contrast objective Plan Apochromat 100x/1.45 N.A. (Nikon). Phase contrast and epifluorescence exposures were 100 ms and 500 ms, respectively.

### Microscopy analysis

Data and statistical tests were performed using Graph Pad Prism 6.0 Software Inc. Automated sacculi detection was achieved using Oufti [89] on inverted WGA signal. Sacculi were detected using a subpixel logarithmic algorithm that was optimized for our images. The localization of NapA was identified by a Gaussian fit to the NapA signal for each cell mesh in the population (spotDetection via Oufti). The NapA locations were then normalized and plotted relative to NapA signal intensity. NapA locations were then binned; shaded region represents the standard deviation for each bin. Data attained from cell meshes were graphed using MatLab 2019a. The codes used to generate Fig 2E are listed in the S1 Text.

### Cryo-electron tomography

Frozen-hydrated specimens were prepared as previously described [35]. Briefly, *B*. *burgdorferi* culture was mixed with 10 nm colloidal gold and was then deposited onto freshly glow-discharged, holey carbon EM grids for 1 min. Grids were blotted with filter paper and then rapidly frozen in liquid ethane, using a homemade gravity-driven plunger apparatus. Frozen-hydrated specimens were imaged at -170°C using a Titan Krios electron microscope (Thermo Fisher) equipped with a field emission gun and a K2 Summit direct detector device (Gatan). The microscope was operated at 300 kV with a magnification of 53,000 ×, resulting in an effective pixel size of 2.7 Å at the specimen level. SerialEM [90] was used to collect tilt series with a cumulative dose of ~60 e^−^/Å^2^. IMOD [91] was used for alignment and reconstruction.

### Stress tests and colony forming units

Initial studies geared towards understanding both permissive and restrictive growth conditions upon stress were performed in microplates with serial dilutions. Each strain was cultured to ~1 x 10^6^ cells/mL and subsequently back diluted to a final concentration of 1 x 10^4^ cells/mL in fresh BSKII containing cell-wall stress. Lysozyme and NaCl were serially diluted 1:1. The final concentration of Lysozyme ranged from 2 mg/mL to 0 mg/mL, while NaCl varied from 500nM to 7.8 mM. We also report the osmolality of each culture media condition that was spiked with NaCl. Osmolality was determined using Fiske Micro-Osmometer Model 210, following manufacturers recommended procedures. Both microtiter plates contained 4 wells of uninoculated media, which served as a negative control. These 96 well plates were allowed to incubate at 37°C under 5% CO_2_ for 6 days. Afterwards, they were placed at ambient conditions for 2 hours before being imaged.

### Colony forming units

Parental and NapA mutant bacteria were cultured to ~6.5 x 10^6^ cells/mL and back diluted to a final starting concentration of 10^6^ cells/mL. Each culture was stressed with Lysozyme (0.37 mg/mL) or NaCl (0.111 M) for 24 hours without additional antibiotic selection. Afterwards, each culture was plated using standard methods [92] and cultured at 37°C under 5% CO_2_. CFUs were determined for strain 5A11 after 3 weeks; *napA* mutant CFUs were counted after 6 weeks.

### Neutrophil migration

The microfluidic competitive chemotaxis-chip (μC^3^) [55] was used to perform each migration assay. This device allowed for the creation of a dual gradient through two opposing chemoattractant reservoirs. The central cell-loading chamber is connected to the two reservoirs by perpendicular cell migration ladders (measured 10 μm wide × 10 μm tall). Device fabrication was as previously [55]. Briefly, two layers of photoresist (SU8, MicroChem), the first one 10 μm thin (corresponding to the migration channels) and the second one 70 μm thick (corresponding to the neutrophil loading chamber) were patterned on one silicon wafer sequentially using two photolithographic masks and processing cycles according to the instructions from the manufacturer. The wafer with patterned photoresist was used as a mold to produce polydimethylsiloxane (PDMS) (Sylgard 184, Elsworth Adhesives, Wilmington, MA) devices, which were then bonded to the base of glass-bottom 6-well plates (MatTek Corp., Ashland, MA), using an oxygen plasma machine (Nordson March, Concord, CA). Prior to each migration assay, the device was primed with fibronectin (Sigma-Aldrich, St. Louis, MO) (11 μg/mL). After priming with fibronectin, each device was covered in 4 mL complete media. Samples were loaded into one chemoattractant reservoir of each corresponding device using a trimmed gel loading pipette tip. Formylmethionine-leucyl-phenylalanine (fMLP, Sigma-Aldrich, St. Louis, MO) (10 nM) and Leukotriene B4 (LTB4, Cayman Chemical, Ann Arbor, MI) (100 nM) served as positive controls in the chemotaxis assay and were loaded in the same manner. The second chemoattractant reservoir was filled with complete media to measure chemorepulsion from the sample. Complete media in both reservoirs served as a negative control in the chemotaxis assay. dHL-60 cells were loaded into the central cell-loading chamber in the ladder device with a gel loading pipette tip. The media was removed and replaced with new complete media after the dHL-60 cells were loaded.

### Chemotaxis imaging and measurements

Each assay was visualized on a fully automated Nikon TiE microscope using a Plan Fluor 10X Ph1 DLL (NA = 0.3) lens with a biochamber heated to 37°C with 5% CO_2_. Image capture was performed using NIS-elements (Nikon Inc., Melville, NY). Experiments were run under the microscope for 5 hours with brightfield and fluorescent images taken at 2-minute intervals. Image analysis of cell migration counts was analyzed automatically using ImageJ (NIH). Cell tracking was conducted using an automated tracker, TrackMate [93] (custom tracking and analysis codes are available for download at https://github.com/boribong/Single-Cell-Migration-Tracking) and ImageJ software (NIH). Cell migration parameters have been defined previously [55].

### Statistical analysis of dHL-60 cell chemotaxis towards NapA

All experiments were performed and replicated at least three times, unless otherwise stated. Statistical analysis was performed using Prism software (GraphPad Software, La Jolla, CA). Data expressed as means ± standard deviations. To compare the migration between the different 5A11 and 5A11/*napA* samples, we used a one-way ANOVA and Turkey’s Multiple Comparison test. To compare the migration toward or away within the different 5A11 and 5A11/*napA* samples, we used a Student’s *t*-test. Differences were considered statistically significant for *p* < 0.05.

### hNOD2 activation assay

To quantify the amount of Muramyl-dipeptide (MDP) present in OMVs and PCs we used the hNOD2 reporter assay (Invivogen) as described previously [16]. Control reactions included 20 ug/mL of the RIP2 inhibitor gefitinib (Sigma Aldrich). MDP (50 ng/mL, Invivogen) served as the positive control.

## Supporting information

S1 Table Summary LC-MS results from PAP preliminary screen. LC-MS results from three biological replicates of PG-associated protein analysis following trypsin cleavage. Data reported represent the mean of all three experiments +/- the standard deviation (SD) for the following categories: MASCOT score; number of unique peptides identified per experiment (# peptides); number of peptide-spectrum matches per experiment (# PSM). Note: Only reliable hits that were observed in two or more experiments were reported.
(DOCX)

S2 Table Summary LC-MS results from stringent PAP screen. LC-MS results from two biological replicates of PG-associated protein analysis following trypsin cleavage. Data reported represent the mean of both experiments +/- the standard deviation (SD) for the following categories: MASCOT score; number of unique peptides identified per experiment (# peptides); number of peptide-spectrum matches per experiment (# PSM). Note that S2 Table differs from S1 Table in the sample preparation. Whereas data presented in S1 Table were from a single SDS solubilization step, S2 Table represents results from a second SDS solubilization step.
(DOCX)

S3 Table Parental clone 5A11 mutations relative to B31 reference genome. All mutations that differ from the B31 type strain are shown with the exception of the hypervariable *vlsE* expression locus. (S) substitution; (A) addition; (D) deletion.
(DOCX)

S4 Table 5A11/*napA* mutations relative to B31 reference genome. All mutations that differ from the B31 type strain are shown with the exception of the hypervariable *vlsE* expression locus. (S) substitution; (A) addition; (D) deletion.
(DOCX)

S5 Table Definition of migratory parameters. (DOCX)

S1 Fig (A) Lysozyme and NaCl stress test. Both 5A11 and 5A11/*napA* strains were grown to 1 x 10^4^ cells/mL in BSK II at 37 ^o^C media prior to adding increasing amounts of Lysozyme (0 to 2 mg/mL) (left) or NaCl (7.8 to 500 mM) (right). Final osmolality of culture media is also shown (380 to 1410 mOsm). Cells were allowed to grow for one week in a 96 well plate prior to growth analysis using spectrophotometry. (B) Growth curves. 5A11 and 5A11/*napA* were grown at a starting concentration of 1 x 10^3^ cells/mL in BSK II media. Cells were enumerated roughly every 24 hours for 10 days with the exception of the first count which occurred 48 hours after inoculation. Note that for data presented in Fig 3 the concentrations of Lysozyme and NaCl tested were in between wells 5–6 and 4–5, respectively.
(TIF)

S2 Fig (A) SDS PAGE and immunoblot analysis of Outer Membrane Vesicle and Protoplasmic Cylinder preparations. Both 5A11 and 5A11/*napA* strains were cultured to late-log, cell were harvested, and fractionated into outer membrane vesicles (OMV) and protoplasmic cylinders (PC). Each preparation was separate by SDS PAGE and visualized by Sypro Ruby stain. Asterisk (*) indicate bands only present in OMVs. (B) NapA Immunoblot of samples prepared as described above.
(TIF)

S3 Fig dHL60 cells migrated toward NapA-associated PG shows less non-dysfunctional migratory patterns in comparison to other preparations. (A) Cells migrating toward NapA-associated PG shows lowest number of cells displaying of non-directional migration (*n* = 23). (B) Cells migrating toward NapA-associated PG shows lowest number of cells showing oscillatory migration (*n* = 13).
(TIFF)

S4 Fig dHL-60 cells migrating toward Nap-A associated PG show higher velocity in comparison to other preparations. Single cell velocity values are plotted over a box plot showing range of values. (A) Cells migrating toward PG bait samples and chemoattractants show Nap-A associated PG has a similar velocity (10.94 ± 4.79 μm/min) to known chemoattractants LTB_4_ (7.04 ± 4.90 μm/min) and fMLP (6.93 ± 4.40 μm/min). (B) Cells migrating away from PG bait samples and chemoattractants show similar velocities. To evaluate differences between responses ANOVA were performed with Turkey’s correction for multiple comparisons (* = *p* < 0.05, *** = *p* < 0.001).
(TIFF)

S5 Fig Phylogenetic analysis of Dps/NapA. (A) Phylogenic analysis of Dps/NapA homologues in *Borreliae*, *Helicobacter pylori*, *Treponema pallidum*, *Leptospira interrogans*, *Yersinia pestis*, *and Escherichia coli*. (B) Amino acid alignment of Dps/NapA homologues from bacteria in A. The Lysine-rich DNA binding domain is underlined (blue) (C) Zoomed in amino acid sequence of the C-terminus of Dps/NapA homologues.
(TIF)

S1 Movie dHL60 cells migrating towards fMLP [10 nM]. (M4V)

S2 Movie dHL60 cells migrating towards LTB4 [10 nM]. (M4V)

S3 Movie dHL60 cells migrating towards 5A11/*napA* PG [125 μg/mL]. (M4V)

S4 Movie dHL60 cells migrating towards trypsin treated 5A11/*napA* PG [125 μg/mL]. (M4V)

S5 Movie dHL60 cells migrating towards 5A11 PG [125 μg/mL]. (M4V)

S6 Movie dHL60 cells migrating towards trypsin treated 5A11 PG [125 μg/mL]. (M4V)

S7 Movie dHL60 cells migrating towards media (negative control). (M4V)

S1 Text Matlab scripts. (DOCX)

## References

[1] Prevention CfDCa. Recent Lyme disease surveillance data 11 7, 2019 [Available from: https://www.cdc.gov/lyme/datasurveillance/recent-surveillance-data.html.

[2] Kugeler KJ, Schwartz AM, Delorey MJ, Mead PS, Hinckley AF. Estimating the Frequency of Lyme Disease Diagnoses, United States, 2010–2018. Emerg Infect Dis. 2021;27(2):616–9. 10.3201/eid2702.202731 33496229 PMC7853543

[3] Mead PS. Epidemiology of Lyme disease. Infectious Disease Clinics. 2015;29(2):187–210. 10.1016/j.idc.2015.02.010 25999219

[4] Eisen RJ, Eisen L, Beard CB. County-scale distribution of I*xodes scapularis* and *Ixodes pacificus* (Acari: *Ixodidae*) in the continental United States. J Med Entomol. 2016;53(2):349–86. 10.1093/jme/tjv237 26783367 PMC4844559

[5] Bisanzio D, Fernández MP, Martello E, Reithinger R, Diuk-Wasser MA. Current and future spatiotemporal patterns of Lyme disease reporting in the northeastern United States. JAMA Netw Open. 2020;3(3):e200319. 10.1001/jamanetworkopen.2020.0319 32125426 PMC7054839

[6] Stanek G, Strle F. Lyme borreliosis-from tick bite to diagnosis and treatment. FEMS Microbiol Rev. 2018;42(3):233–58. 10.1093/femsre/fux047 29893904

[7] Hatchette TF, Davis I, Johnston BL. Lyme disease: clinical diagnosis and treatment. Can Commun Dis Rep. 2014;40(11):194–208. 10.14745/ccdr.v40i11a01 29769842 PMC5864449

[8] Wormser GP, Dattwyler RJ, Shapiro ED, Halperin JJ, Steere AC, Klempner MS, et al. The clinical assessment, treatment, and prevention of lyme disease, human granulocytic anaplasmosis, and babesiosis: clinical practice guidelines by the Infectious Diseases Society of America. Clin Infect Dis. 2006;43(9):1089–134. 10.1086/508667 17029130

[9] Steere AC, Strle F, Wormser GP, Hu LT, Branda JA, Hovius JW, et al. Lyme borreliosis. Nat Rev Dis Primers. 2016;2:16090. 10.1038/nrdp.2016.90 27976670 PMC5539539

[10] Steere AC. Posttreatment Lyme disease syndromes: distinct pathogenesis caused by maladaptive host responses. J Clin Invest. 2020;130(5):2148–51. 10.1172/JCI138062 32281948 PMC7190968

[11] Sulka KB, Strle K, Crowley JT, Lochhead RB, Anthony R, Steere AC. Correlation of Lyme disease-associated IgG4 autoantibodies with synovial pathology in antibiotic-refractory Lyme arthritis. Arthritis Rheumatol. 2018;70(11):1835–46. 10.1002/art.40566 29790305 PMC6203610

[12] Drouin EE, Seward RJ, Strle K, McHugh G, Katchar K, Londoño D, et al. A novel human autoantigen, endothelial cell growth factor, is a target of T and B cell responses in patients with Lyme disease. Arthritis Rheum. 2013;65(1):186–96. 10.1002/art.37732 23044924 PMC3535550

[13] Crowley JT, Strle K, Drouin EE, Pianta A, Arvikar SL, Wang Q, et al. Matrix metalloproteinase-10 is a target of T and B cell responses that correlate with synovial pathology in patients with antibiotic-refractory Lyme arthritis. J Autoimmun. 2016;69:24–37. 10.1016/j.jaut.2016.02.005 26922382 PMC4826816

[14] Pianta A, Drouin EE, Crowley JT, Arvikar S, Strle K, Costello CE, et al. Annexin A2 is a target of autoimmune T and B cell responses associated with synovial fibroblast proliferation in patients with antibiotic-refractory Lyme arthritis. Clin Immunol. 2015;160(2):336–41. 10.1016/j.clim.2015.07.005 26187145 PMC4582008

[15] Strle K, Shin JJ, Glickstein LJ, Steere AC. Association of a Toll-like receptor 1 polymorphism with heightened Th1 inflammatory responses and antibiotic-refractory Lyme arthritis. Arthritis Rheum. 2012;64(5):1497–507. 10.1002/art.34383 22246581 PMC3338893

[16] Jutras BL, Lochhead RB, Kloos ZA, Biboy J, Strle K, Booth CJ, et al. *Borrelia burgdorferi* peptidoglycan is a persistent antigen in patients with Lyme arthritis. Proc Natl Acad Sci U S A. 2019;116(27):13498–507. 10.1073/pnas.1904170116 31209025 PMC6613144

[17] Costerton JW, Ingram JM, Cheng KJ. Structure and function of the cell envelope of gram-negative bacteria. Bacteriol Rev. 1974;38(1):87–110. 4601163 10.1128/br.38.1.87-110.1974 PMC413842

[18] Gan L, Chen S, Jensen GJ. Molecular organization of Gram-negative peptidoglycan. Proc Natl Acad Sci U S A. 2008;105(48):18953–7. 10.1073/pnas.0808035105 19033194 PMC2596242

[19] Parry BR, Surovtsev IV, Cabeen MT, O’Hern CS, Dufresne ER, Jacobs-Wagner C. The bacterial cytoplasm has glass-like properties and is fluidized by metabolic activity. Cell. 2014;156(1–2):183–94. 10.1016/j.cell.2013.11.028 24361104 PMC3956598

[20] Kovacs-Simon A, Titball RW, Michell SL. Lipoproteins of bacterial pathogens. Infect Immun. 2011;79(2):548–61. 10.1128/IAI.00682-10 20974828 PMC3028857

[21] Cascales E, Bernadac A, Gavioli M, Lazzaroni JC, Lloubes R. Pal lipoprotein of *Escherichia coli* plays a major role in outer membrane integrity. J Bacteriol. 2002;184(3):754–9. 10.1128/jb.184.3.754-759.2002 11790745 PMC139529

[22] Haake DA. Spirochaetal lipoproteins and pathogenesis. Microbiol-Uk. 2000;146:1491–504. 10.1099/00221287-146-7-1491 10878114 PMC2664406

[23] Henderson B, Martin A. Bacterial virulence in the moonlight: multitasking bacterial moonlighting proteins are virulence determinants in infectious disease. Infect Immun. 2011;79(9):3476–91. 10.1128/IAI.00179-11 21646455 PMC3165470

[24] Fraser CM, Casjens S, Huang WM, Sutton GG, Clayton R, Lathigra R, et al. Genomic sequence of a Lyme disease spirochaete, *Borrelia burgdorferi*. Nature. 1997;390(6660):580–6. 10.1038/37551 9403685

[25] Casjens S, Palmer N, van Vugt R, Huang WM, Stevenson B, Rosa P, et al. A bacterial genome in flux: the twelve linear and nine circular extrachromosomal DNAs in an infectious isolate of the Lyme disease spirochete *Borrelia burgdorferi*. Mol Microbiol. 2000;35(3):490–516. 10.1046/j.1365-2958.2000.01698.x 10672174

[26] Radolf JD, Caimano MJ, Stevenson B, Hu LT. Of ticks, mice and men: understanding the dual-host lifestyle of Lyme disease spirochaetes. Nat Rev Microbiol. 2012;10(2):87–99. 10.1038/nrmicro2714 22230951 PMC3313462

[27] Pappas CJ, Iyer R, Petzke MM, Caimano MJ, Radolf JD, Schwartz I. *Borrelia burgdorferi* requires glycerol for maximum fitness during the tick phase of the enzootic cycle. PLoS Pathog. 2011;7(7):e1002102. 10.1371/journal.ppat.1002102 21750672 PMC3131272

[28] Crowley JT, Toledo AM, LaRocca TJ, Coleman JL, London E, Benach JL. Lipid exchange between *Borrelia burgdorferi* and host cells. PLoS Pathog. 2013;9(1):e1003109. 10.1371/journal.ppat.1003109 23326230 PMC3542181

[29] Zuckert WR. Secretion of bacterial lipoproteins: through the cytoplasmic membrane, the periplasm and beyond. Biochim Biophys Acta. 2014;1843(8):1509–16. 10.1016/j.bbamcr.2014.04.022 24780125 PMC4070597

[30] Wolgemuth CW, Charon NW, Goldstein SF, Goldstein RE. The flagellar cytoskeleton of the spirochetes. J Mol Microbiol Biotechnol. 2006;11(3–5):221–7. 10.1159/000094056 16983197

[31] Motaleb MA, Corum L, Bono JL, Elias AF, Rosa P, Samuels DS, et al. *Borrelia burgdorferi* periplasmic flagella have both skeletal and motility functions. Proc Natl Acad Sci U S A. 2000;97(20):10899–904. 10.1073/pnas.200221797 10995478 PMC27121

[32] Beck G, Benach JL, Habicht GS. Isolation, preliminary chemical characterization, and biological activity of *Borrelia burgdorferi* peptidoglycan. Biochem Biophys Res Commun. 1990;167(1):89–95. 10.1016/0006-291x(90)91734-a 2310405

[33] Jutras BL, Scott M, Parry B, Biboy J, Gray J, Vollmer W, et al. Lyme disease and relapsing fever *Borrelia* elongate through zones of peptidoglycan synthesis that mark division sites of daughter cells. Proc Natl Acad Sci U S A. 2016;113(33):9162–70. 10.1073/pnas.1610805113 27506799 PMC4995948

[34] Li X, Pal U, Ramamoorthi N, Liu X, Desrosiers DC, Eggers CH, et al. The Lyme disease agent *Borrelia burgdorferi* requires BB0690, a Dps homologue, to persist within ticks. Mol Microbiol. 2007;63(3):694–710. 10.1111/j.1365-2958.2006.05550.x 17181780

[35] Seshu J, Boylan JA, Gherardini FC, Skare JT. Dissolved oxygen levels alter gene expression and antigen profiles in *Borrelia burgdorferi*. Infect Immun. 2004;72(3):1580–6. 10.1128/iai.72.3.1580-1586.2004 14977964 PMC356058

[36] Calhoun LN, Kwon YM. Structure, function and regulation of the DNA-binding protein Dps and its role in acid and oxidative stress resistance in *Escherichia coli*: a review. J Appl Microbiol. 2011;110(2):375–86. 10.1111/j.1365-2672.2010.04890.x 21143355

[37] Haikarainen T, Papageorgiou AC. Dps-like proteins: structural and functional insights into a versatile protein family. Cell Mol Life Sci. 2010;67(3):341–51. 10.1007/s00018-009-0168-2 19826764 PMC11115558

[38] Cooksley C, Jenks PJ, Green A, Cockayne A, Logan RP, Hardie KR. NapA protects *Helicobacter pylori* from oxidative stress damage, and its production is influenced by the ferric uptake regulator. J Med Microbiol. 2003;52(Pt 6):461–9. 10.1099/jmm.0.05070-0 12748264

[39] Wang P, Lutton A, Olesik J, Vali H, Li X. A novel iron- and copper-binding protein in the Lyme disease spirochaete. Mol Microbiol. 2012;86(6):1441–51. 10.1111/mmi.12068 23061404

[40] Codolo G, Amedei A, Steere AC, Papinutto E, Cappon A, Polenghi A, et al. *Borrelia burgdorferi* NapA-driven Th17 cell inflammation in lyme arthritis. Arthritis Rheum. 2008;58(11):3609–17. 10.1002/art.23972 18975343

[41] Codolo G, Papinutto E, Polenghi A, D’Elios MM, Zanotti G, de Bernard M. Structure and immunomodulatory property relationship in NapA of *Borrelia burgdorferi*. Biochim Biophys Acta. 2010;1804(12):2191–7. 10.1016/j.bbapap.2010.09.004 20851780

[42] Codolo G, Bossi F, Durigutto P, Bella CD, Fischetti F, Amedei A, et al. Orchestration of inflammation and adaptive immunity in *Borrelia burgdorferi*-induced arthritis by neutrophil-activating protein A. Arthritis Rheum. 2013;65(5):1232–42. 10.1002/art.37875 23371320

[43] Troxell B, Ye M, Yang Y, Carrasco SE, Lou Y, Yang XF. Manganese and zinc regulate virulence determinants in *Borrelia burgdorferi*. Infect Immun. 2013;81(8):2743–52. 10.1128/IAI.00507-13 23690398 PMC3719580

[44] Takacs N C, Kloos A Z, Scott M, Rosa A P, Jacobs-Wagner C. Fluorescent proteins, promoters, and selectable markers for applications in the Lyme disease spirochete *Borrelia burgdorferi*. 2018;84(24):e01824–18. 10.1128/AEM.01824-18 PMC6275353 30315081

[45] Chang Y, Moon KH, Zhao X, Norris SJ, Motaleb MA, Liu J. Structural insights into flagellar stator-rotor interactions. Elife. 2019;8. 10.7554/eLife.48979 31313986 PMC6663468

[46] Mulay V, Caimano MJ, Liveris D, Desrosiers DC, Radolf JD, Schwartz I. *Borrelia burgdorferi* BBA74, a periplasmic protein associated with the outer membrane, lacks porin-like properties. J Bacteriol. 2007;189(5):2063–8. 10.1128/JB.01239-06 17189354 PMC1855751

[47] Koetsveld J, Draga ROP, Wagemakers A, Manger A, Oei A, Visser CE, et al. In Vitro Susceptibility of the Relapsing-Fever Spirochete *Borrelia miyamotoi* to Antimicrobial Agents. Antimicrob Agents Chemother. 2017;61(9). 10.1128/AAC.00535-17 28674060 PMC5571331

[48] Dever LL, Jorgensen JH, Barbour AG. In vitro antimicrobial susceptibility testing of *Borrelia burgdorferi*: a microdilution MIC method and time-kill studies. J Clin Microbiol. 1992;30(10):2692–7. 10.1128/JCM.30.10.2692-2697.1992 1400969 PMC270500

[49] Skare JT, Shang ES, Foley DM, Blanco DR, Champion CI, Mirzabekov T, et al. Virulent strain associated outer membrane proteins of *Borrelia burgdorferi*. J Clin Invest. 1995;96(5):2380–92. 10.1172/JCI118295 7593626 PMC185890

[50] Yang X, Promnares K, Qin J, He M, Shroder DY, Kariu T, et al. Characterization of multiprotein complexes of the *Borrelia burgdorferi* outer membrane vesicles. J Proteome Res. 2011;10(10):4556–66. 10.1021/pr200395b 21875077 PMC3189302

[51] Tigno-Aranjuez JT, Asara JM, Abbott DW. Inhibition of RIP2’s tyrosine kinase activity limits NOD2-driven cytokine responses. Genes Dev. 2010;24(23):2666–77. 10.1101/gad.1964410 21123652 PMC2994040

[52] Schwechheimer C, Kuehn MJ. Outer-membrane vesicles from Gram-negative bacteria: biogenesis and functions. Nat Rev Microbiol. 2015;13(10):605–19. 10.1038/nrmicro3525 26373371 PMC5308417

[53] Oosting M, ter Hofstede H, van de Veerdonk FL, Sturm P, Kullberg BJ, van der Meer JW, et al. Role of interleukin-23 (IL-23) receptor signaling for IL-17 responses in human Lyme disease. Infect Immun. 2011;79(11):4681–7. 10.1128/IAI.05242-11 21896776 PMC3257938

[54] Lochhead RB, Ordoñez D, Arvikar SL, Aversa JM, Oh LS, Heyworth B, et al. Interferon-gamma production in Lyme arthritis synovial tissue promotes differentiation of fibroblast-like synoviocytes into immune effector cells. Cell Microbiol. 2019;21(2):e12992. 10.1111/cmi.12992 30550623 PMC6336510

[55] Boribong BP, Lenzi MJ, Li L, Jones CN. Super-low dose lipopolysaccharide dysregulates neutrophil migratory decision-making. Front Immunol. 2019;10:359. 10.3389/fimmu.2019.00359 30915068 PMC6422936

[56] Schulze RJ, Zückert WR. *Borrelia burgdorferi* lipoproteins are secreted to the outer surface by default. Mol Microbiol. 2006;59(5):1473–84. 10.1111/j.1365-2958.2006.05039.x 16468989

[57] Zückert WR. Protein Secretion in Spirochetes. Microbiol Spectr. 2019;7(3). 10.1128/microbiolspec.PSIB-0026-2019 31198130 PMC6579041

[58] Ratet G, Santecchia I, Fanton d’Andon M, Vernel-Pauillac F, Wheeler R, Lenormand P, et al. LipL21 lipoprotein binding to peptidoglycan enables *Leptospira interrogans* to escape NOD1 and NOD2 recognition. PLoS Pathog. 2017;13(12):e1006725. 10.1371/journal.ppat.1006725 29211798 PMC5764436

[59] Chou S, Daugherty MD, Peterson SB, Biboy J, Yang Y, Jutras BL, et al. Transferred interbacterial antagonism genes augment eukaryotic innate immune function. Nature. 2015;518(7537):98–101. 10.1038/nature13965 25470067 PMC4713192

[60] Oliva Chavez AS, Shaw DK, Munderloh UG, Pedra JH. Tick humoral responses: Marching to the beat of a different drummer. Front Microbiol. 2017;8:223. 10.3389/fmicb.2017.00223 28261180 PMC5306392

[61] Kim TK, Tirloni L, Pinto AF, Moresco J, Yates JR 3rd, da Silva Vaz I Jr, et al. *Ixodes scapularis* tick saliva proteins sequentially secreted every 24 h during blood feeding. PLoS Negl Trop Dis. 2016;10(1):e0004323. 10.1371/journal.pntd.0004323 26751078 PMC4709002

[62] Dramsi S, Magnet S, Davison S, Arthur M. Covalent attachment of proteins to peptidoglycan. FEMS Microbiol Rev. 2008;32(2):307–20. 10.1111/j.1574-6976.2008.00102.x 18266854

[63] Cafiero T, Toledo A. *Borrelia burgdorferi* surface exposed GroEL is a multifunctional protein. Pathogens. 2021;10(2):226. 10.3390/pathogens10020226 33670728 PMC7922809

[64] Toledo A, Coleman JL, Kuhlow CJ, Crowley JT, Benach JL. The enolase of *Borrelia burgdorferi* is a plasminogen receptor released in outer membrane vesicles. Infect Immun. 2012;80(1):359–68. 10.1128/IAI.05836-11 22083700 PMC3255694

[65] Nogueira SV, Smith AA, Qin JH, Pal U. A surface enolase participates in *Borrelia burgdorferi*-plasminogen interaction and contributes to pathogen survival within feeding ticks. Infect Immun. 2012;80(1):82–90. 10.1128/IAI.05671-11 22025510 PMC3255677

[66] Floden AM, Watt JA, Brissette CA. *Borrelia burgdorferi* enolase is a surface-exposed plasminogen binding protein. PLoS One. 2011;6(11):e27502. 10.1371/journal.pone.0027502 22087329 PMC3210797

[67] Bendtsen JD, Kiemer L, Fausbøll A, Brunak S. Non-classical protein secretion in bacteria. BMC Microbiol. 2005;5:58. 10.1186/1471-2180-5-58 16212653 PMC1266369

[68] Notti RQ, Stebbins CE. The structure and function of type III secretion systems. Microbiol Spectr. 2016;4(1):10. 10.1128/microbiolspec.VMBF-0004-2015 26999392 PMC4804468

[69] Young GM, Schmiel DH, Miller VL. A new pathway for the secretion of virulence factors by bacteria: The flagellar export apparatus functions as a protein-secretion system. Proc Natl Acad Sci U S A. 1999;96(11):6456–646. 10.1073/pnas.96.11.6456 10339609 PMC26903

[70] Young BM, Young GM. YplA is exported by the Ysc, Ysa, and flagellar type III secretion systems of *Yersinia enterocolitica*. J Bacteriol. 2002;184(5):1324–34. 10.1128/jb.184.5.1324-1334.2002 11844761 PMC134849

[71] Konkel ME, Klena JD, Rivera-Amill V, Monteville MR, Biswas D, Raphael B, et al. Secretion of virulence proteins from *Campylobacter jejuni* is dependent on a functional flagellar export apparatus. J Bacteriol. 2004;186(11):3296–303. 10.1128/JB.186.11.3296-3303.2004 15150214 PMC415756

[72] Barrero-Tobon AM, Hendrixson DR. Flagellar biosynthesis exerts temporal regulation of secretion of specific *Campylobacter jejuni* colonization and virulence determinants. Mol Microbiol. 2014;93(5):957–74. 10.1111/mmi.12711 25041103 PMC4150830

[73] Abby SS, Rocha EP. The non-flagellar type III secretion system evolved from the bacterial flagellum and diversified into host-cell adapted systems. PLoS Genet. 2012;8(9):e1002983. 10.1371/journal.pgen.1002983 23028376 PMC3459982

[74] Feltcher ME, Sullivan JT, Braunstein M. Protein export systems of *Mycobacterium tuberculosis*: novel targets for drug development? Future Microbiol. 2010;5(10):1581–97. 10.2217/fmb.10.112 21073315 PMC3034451

[75] Kudva R, Denks K, Kuhn P, Vogt A, Müller M, Koch H-G. Protein translocation across the inner membrane of Gram-negative bacteria: the Sec and Tat dependent protein transport pathways. Research in microbiology. 2013;164(6):505–34. 10.1016/j.resmic.2013.03.016 23567322

[76] Posey JE, Gherardini FC. Lack of a role for iron in the Lyme disease pathogen. Science. 2000; 288(5471):1651–3. 10.1126/science.288.5471.1651 10834845

[77] Samuels DS, Radolf JD. Who is the BosR around here anyway? Mol Microbiol. 2009;74(6):1295–9. 10.1111/j.1365-2958.2009.06971.x 19943896 PMC3005592

[78] Samuels DS. Gene regulation in *Borrelia burgdorferi*. Annu Rev Microbiol. 2011;65:479–99. 10.1146/annurev.micro.112408.134040 21801026

[79] Oosting M, Buffen K, van der Meer JW, Netea MG, Joosten LA. Innate immunity networks during infection with *Borrelia burgdorferi*. Crit Rev Microbiol. 2016;42(2):233–44. 10.3109/1040841X.2014.929563 24963691

[80] Benach JL, Fleit HB, Habicht GS, Coleman JL, Bosler EM, Lane BP. Interactions of phagocytes with the Lyme disease spirochete: role of the Fc receptor. J Infect Dis. 1984;150(4):497–507. 10.1093/infdis/150.4.497 6386995

[81] Menten-Dedoyart C, Faccinetto C, Golovchenko M, Dupiereux I, Van Lerberghe PB, Dubois S, et al. Neutrophil extracellular traps entrap and kill *Borrelia burgdorferi* sensu stricto spirochetes and are not affected by *Ixodes ricinus* tick saliva. J Immunol. 2012;189(11):5393–401. 10.4049/jimmunol.1103771 23109724

[82] Nair S, Finkel SE. Dps protects cells against multiple stresses during stationary phase. J Bacteriol. 2004;186(13):4192–8. 10.1128/JB.186.13.4192-4198.2004 15205421 PMC421617

[83] Wang G, Hong Y, Olczak A, Maier SE, Maier RJ. Dual Roles of *Helicobacter pylori* NapA in inducing and combating oxidative stress. Infect Immun. 2006;74(12):6839–46. 10.1128/IAI.00991-06 17030577 PMC1698064

[84] Grant RA, Filman DJ, Finkel SE, Kolter R, Hogle JM. The crystal structure of Dps, a ferritin homolog that binds and protects DNA. Nat Struct Biol. 1998;5(4):294–303. 10.1038/nsb0498-294 9546221

[85] Karas VO, Westerlaken I, Meyer AS. The DNA-Binding Protein from Starved Cells (Dps) Utilizes Dual Functions To Defend Cells against Multiple Stresses. J Bacteriol. 2015;197(19):3206–15. 10.1128/JB.00475-15 26216848 PMC4560292

[86] Purser JE, Norris SJ. Correlation between plasmid content and infectivity in *Borrelia burgdorferi*. Proc Natl Acad Sci U S A. 2000;97(25):13865–70. 10.1073/pnas.97.25.13865 11106398 PMC17667

[87] Samuels DS. Electrotransformation of the spirochete *Borrelia burgdorferi*. Methods Mol Biol. 1995;47:253–9. 10.1385/0-89603-310-4:253 7550741 PMC5815860

[88] Caimano MJ, Eggers CH, Gonzalez CA, Radolf JD. Alternate sigma factor RpoS is required for the in vivo-specific repression of *Borrelia burgdorferi* plasmid lp54-borne ospA and lp6.6 genes. J Bacteriol. 2005;187(22):7845–52. 10.1128/JB.187.22.7845-7852.2005 16267308 PMC1280317

[89] Paintdakhi A, Parry B, Campos M, Irnov I, Elf J, Surovtsev I, et al. Oufti: an integrated software package for high-accuracy, high-throughput quantitative microscopy analysis. Mol Microbiol. 2016;99(4):767–77. 10.1111/mmi.13264 26538279 PMC4752901

[90] Mastronarde DN. Automated electron microscope tomography using robust prediction of specimen movements. J Struct Biol. 2005;152(1):36–51. 10.1016/j.jsb.2005.07.007 16182563

[91] Kremer JR, Mastronarde DN, McIntosh JR. Computer visualization of three-dimensional image data using IMOD. J Struct Biol. 1996;116(1):71–6. 10.1006/jsbi.1996.0013 8742726

[92] Zuckert WR. Laboratory maintenance of *Borrelia burgdorferi*. Curr Protoc Microbiol. 2007;Chapter 12:Unit 12C 1. 10.1002/9780471729259.mc12c01s4 18770608

[93] Tinevez JY, Perry N, Schindelin J, Hoopes GM, Reynolds GD, Laplantine E, et al. TrackMate: An open and extensible platform for single-particle tracking. Methods. 2017;115:80–90. 10.1016/j.ymeth.2016.09.016 27713081

