# Supplementary material for: The peptidoglycan-associated protein NapA plays an important role in the envelope integrity and in the pathogenesis of the lyme disease spirochete
Source: PLoS Pathog. 2021 May 13;17(5):e1009546. doi: 10.1371/journal.ppat.1009546 (PMC8118282; doi:10.1371/journal.ppat.1009546)
Supplement: S1 Table — LC-MS results from three biological replicates of PG-associated protein analysis following trypsin cleavage. Data reported represent the mean of all three experiments +/- the standard deviation (SD) for the following categories: MASCOT score; number of unique peptides identified per experiment (# peptides); number of peptide-spectrum matches per experiment (# PSM). Note: Only reliable hits that were observed in two or more experiments were reported. (DOCX) [file ppat.1009546.s001.docx]

**Accession Name MW Observed MASCOT** **# peptides # PSM**

(kDa) mean(+/-SD) mean(+/-SD) mean(+/-SD)

BB0690 NapA 21.3 3/3 244 (175) 3.7(2) 23(20)

BB0744 P83/P100 antigen 79.9 2/3 399 (656) 3.7(5.5) 20(33)

BB0476 Elongation Factor Tu 43.6 2/3 227(272) 5(5) 15(18)

BB0388 RpoC 155 2/3 118(142) 5(1.4) 12(1.4)
