# Supplementary material for: The peptidoglycan-associated protein NapA plays an important role in the envelope integrity and in the pathogenesis of the lyme disease spirochete
Source: PLoS Pathog. 2021 May 13;17(5):e1009546. doi: 10.1371/journal.ppat.1009546 (PMC8118282; doi:10.1371/journal.ppat.1009546)
Supplement: S2 Table — LC-MS results from two biological replicates of PG-associated protein analysis following trypsin cleavage. Data reported represent the mean of both experiments +/- the standard deviation (SD) for the following categories: MASCOT score; number of unique peptides identified per experiment (# peptides); number of peptide-spectrum matches per experiment (# PSM). Note that S2 Table differs from S1 Table in the sample preparation. Whereas data presented in S1 Table were from a single SDS solubilization step, S2 Table represents results from a second SDS solubilization step. (DOCX) [file ppat.1009546.s002.docx]

**Accession Name MW Observed MASCOT** **# peptides # PSM**

(kDa) mean(+/-SD) mean(+/-SD) mean(+/-SD)

BB0690 NapA 21.3 2/2 181 (34) 3.5(2) 12(3)

BB0744 P83/P100 antigen 79.9 2/2 54 (61) 1.5(0.7) 3(1.4)
