# Supplementary material for: The peptidoglycan-associated protein NapA plays an important role in the envelope integrity and in the pathogenesis of the lyme disease spirochete
Source: PLoS Pathog. 2021 May 13;17(5):e1009546. doi: 10.1371/journal.ppat.1009546 (PMC8118282; doi:10.1371/journal.ppat.1009546)
Supplement: S3 Table — All mutations that differ from the B31 type strain are shown with the exception of the hypervariable vlsE expression locus. (S) substitution; (A) addition; (D) deletion. (DOCX) [file ppat.1009546.s003.docx]

**Mutation Event Coordinate Location Result**

S G🡪T 10,883 Coding-OspB G199V

S C🡪A 28,269 Coding-BBN41 Q82K

S C🡪T 17,924 Intergenic —

A +C 3,140 Intergenic ­—

S G🡪A 56,157 Coding-BB0059 V42I

A +A 138,870 Intergenic —

A +T 366,152 Intergenic —

A +A 422,314 Intergenic —

D -G 515,969 Intergenic —

D -C 516,002 Intergenic —

D -G 516,098 Intergenic —

S T🡪A 528,031 Intergenic —

A +G 532,509 Intergenic —

A +T 540,032 Intergenic —

S T🡪G 747,897 Intergenic —

A +A 862,670 Intergenic —
