# Supplementary material for: The peptidoglycan-associated protein NapA plays an important role in the envelope integrity and in the pathogenesis of the lyme disease spirochete
Source: PLoS Pathog. 2021 May 13;17(5):e1009546. doi: 10.1371/journal.ppat.1009546 (PMC8118282; doi:10.1371/journal.ppat.1009546)
Supplement: S5 Table — (DOCX) [file ppat.1009546.s005.docx]

| **Migratory Phenotype** | **Definition** | **Units** |
| --- | --- | --- |
| Percentage of Cells Migrated | Number of Cells Migrated/Average of Cells in Central Loading Chamber × 100 | Percentage (%) |
| dHL-60 Cell Velocity | Distance Cell Traveled/Time Elapsed | µm/min |
| Non-Directional Migration | dHL-60 cells that enter the cell mazes | Number of Cells |
| Oscillatory Migration | Cells that change direction in the x or y plane ≥ 3 times | Number of Cells |
