# Supplementary figures and images for: The peptidoglycan-associated protein NapA plays an important role in the envelope integrity and in the pathogenesis of the lyme disease spirochete
Source: PLoS Pathog. 2021 May 13;17(5):e1009546. doi: 10.1371/journal.ppat.1009546 (PMC8118282; doi:10.1371/journal.ppat.1009546)

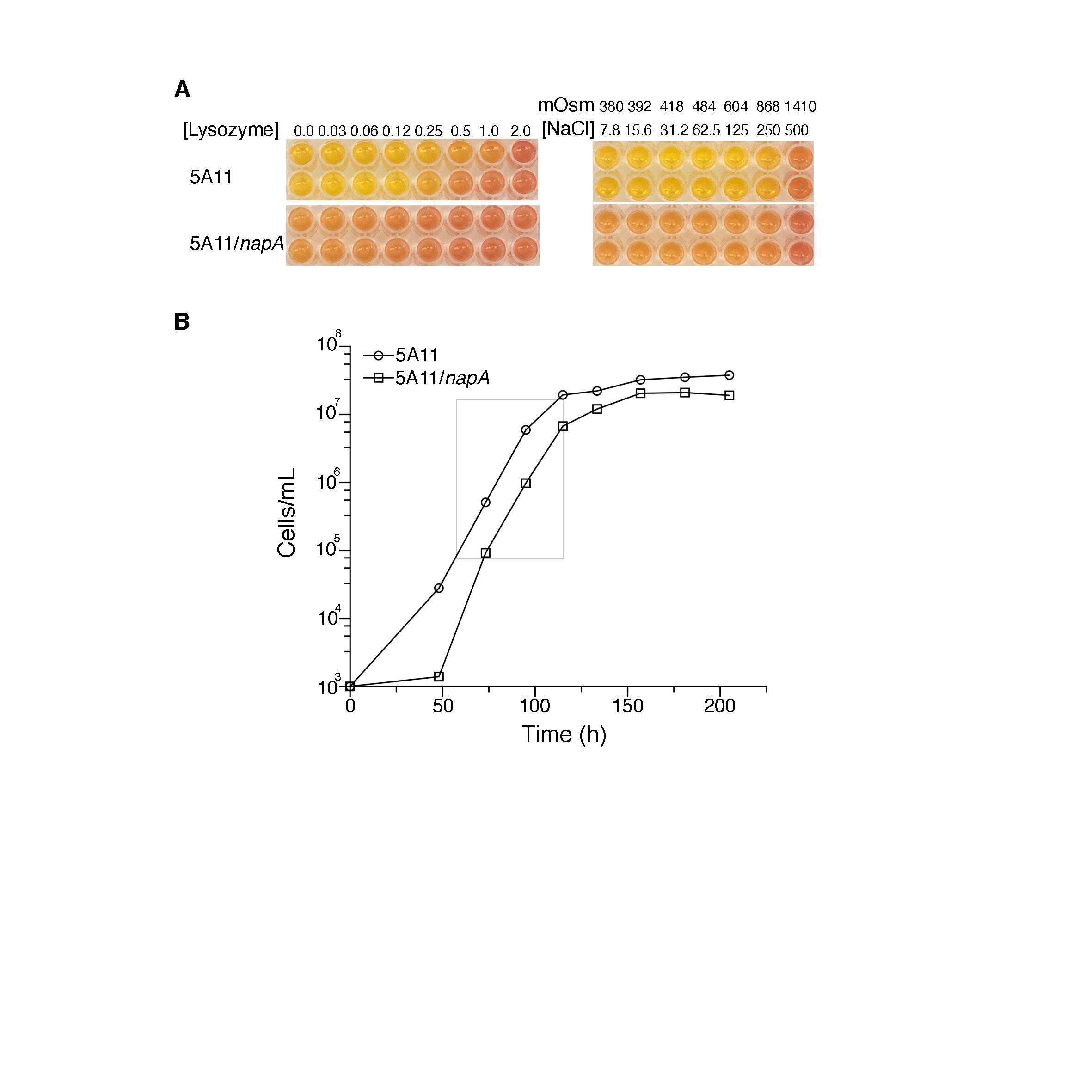

Supplement: S1 Fig — (A) Lysozyme and NaCl stress test. Both 5A11 and 5A11/napA strains were grown to 1 x 10^4^ cells/mL in BSK II at 37 oC media prior to adding increasing amounts of Lysozyme (0 to 2 mg/mL) (left) or NaCl (7.8 to 500 mM) (right). Final osmolality of culture media is also shown (380 to 1410 mOsm). Cells were allowed to grow for one week in a 96 well plate prior to growth analysis using spectrophotometry. (B) Growth curves. 5A11 and 5A11/napA were grown at a starting concentration of 1 x 10^3^ cells/mL in BSK II media. Cells were enumerated roughly every 24 hours for 10 days with the exception of the first count which occurred 48 hours after inoculation. Note that for data presented in Fig 3 the concentrations of Lysozyme and NaCl tested were in between wells 5–6 and 4–5, respectively. (TIF) [file ppat.1009546.s006.tif]

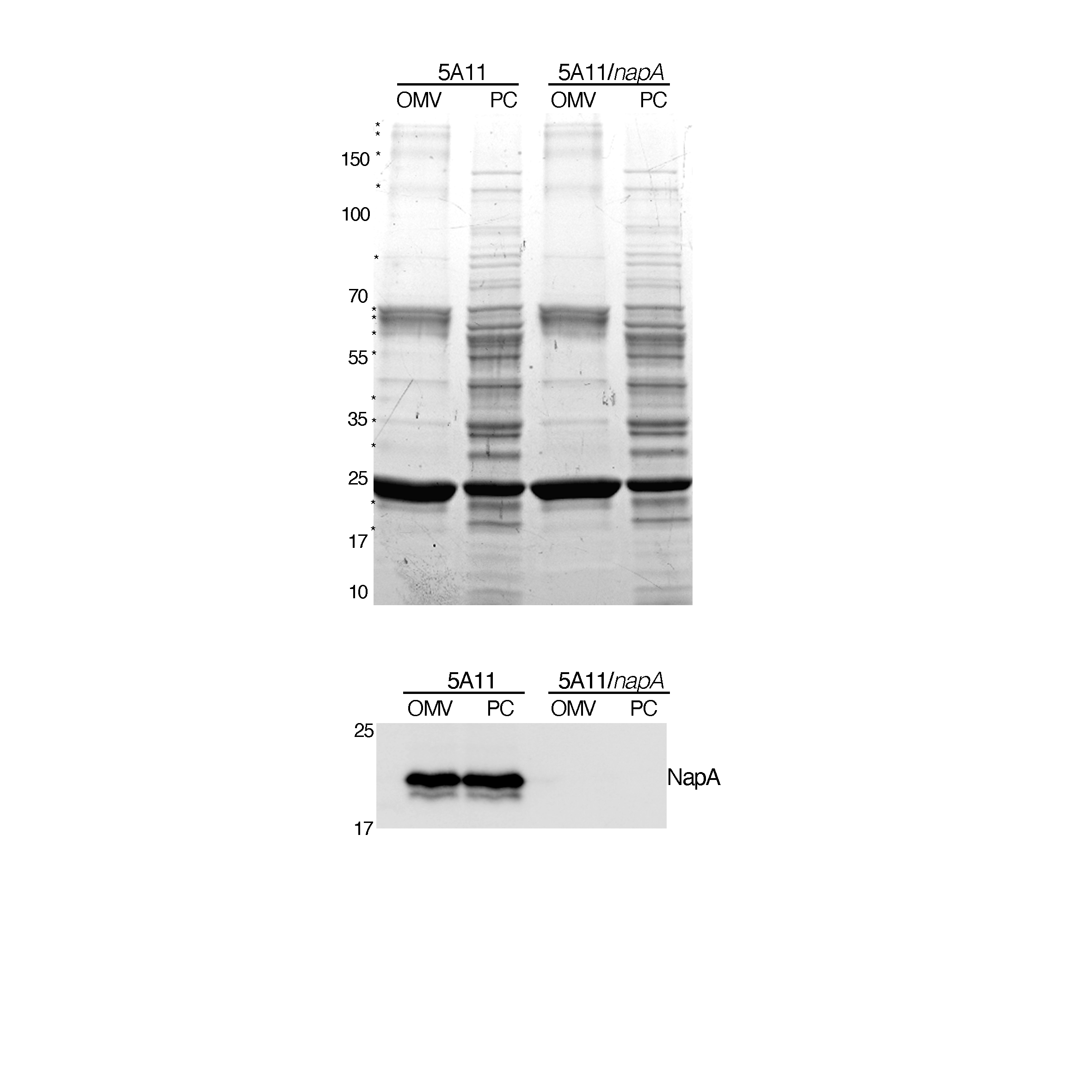

Supplement: S2 Fig — (A) SDS PAGE and immunoblot analysis of Outer Membrane Vesicle and Protoplasmic Cylinder preparations. Both 5A11 and 5A11/napA strains were cultured to late-log, cell were harvested, and fractionated into outer membrane vesicles (OMV) and protoplasmic cylinders (PC). Each preparation was separate by SDS PAGE and visualized by Sypro Ruby stain. Asterisk (*) indicate bands only present in OMVs. (B) NapA Immunoblot of samples prepared as described above. (TIF) [file ppat.1009546.s007.tif]

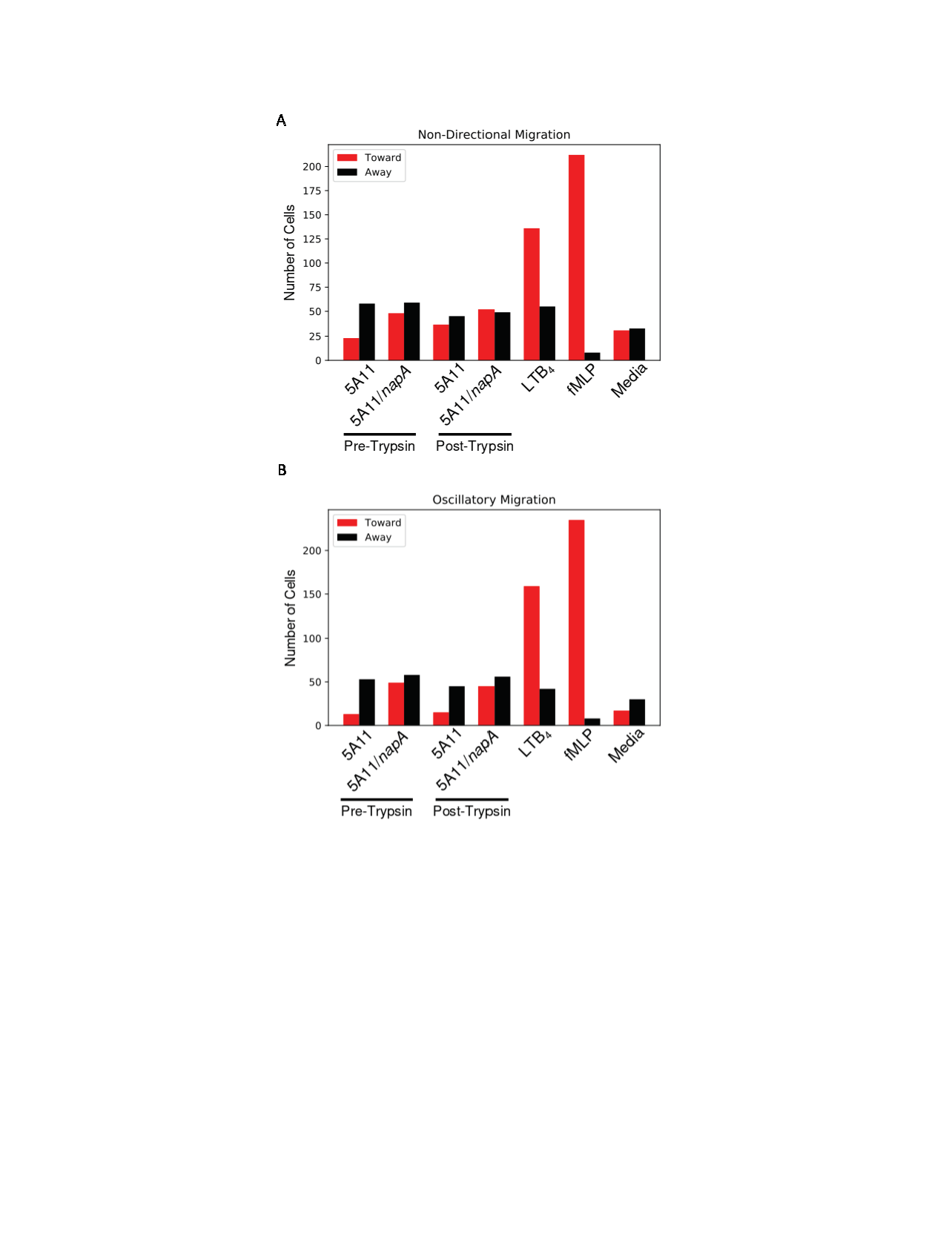

Supplement: S3 Fig — (A) Cells migrating toward NapA-associated PG shows lowest number of cells displaying of non-directional migration (n = 23). (B) Cells migrating toward NapA-associated PG shows lowest number of cells showing oscillatory migration (n = 13). (TIFF) [file ppat.1009546.s008.tiff]

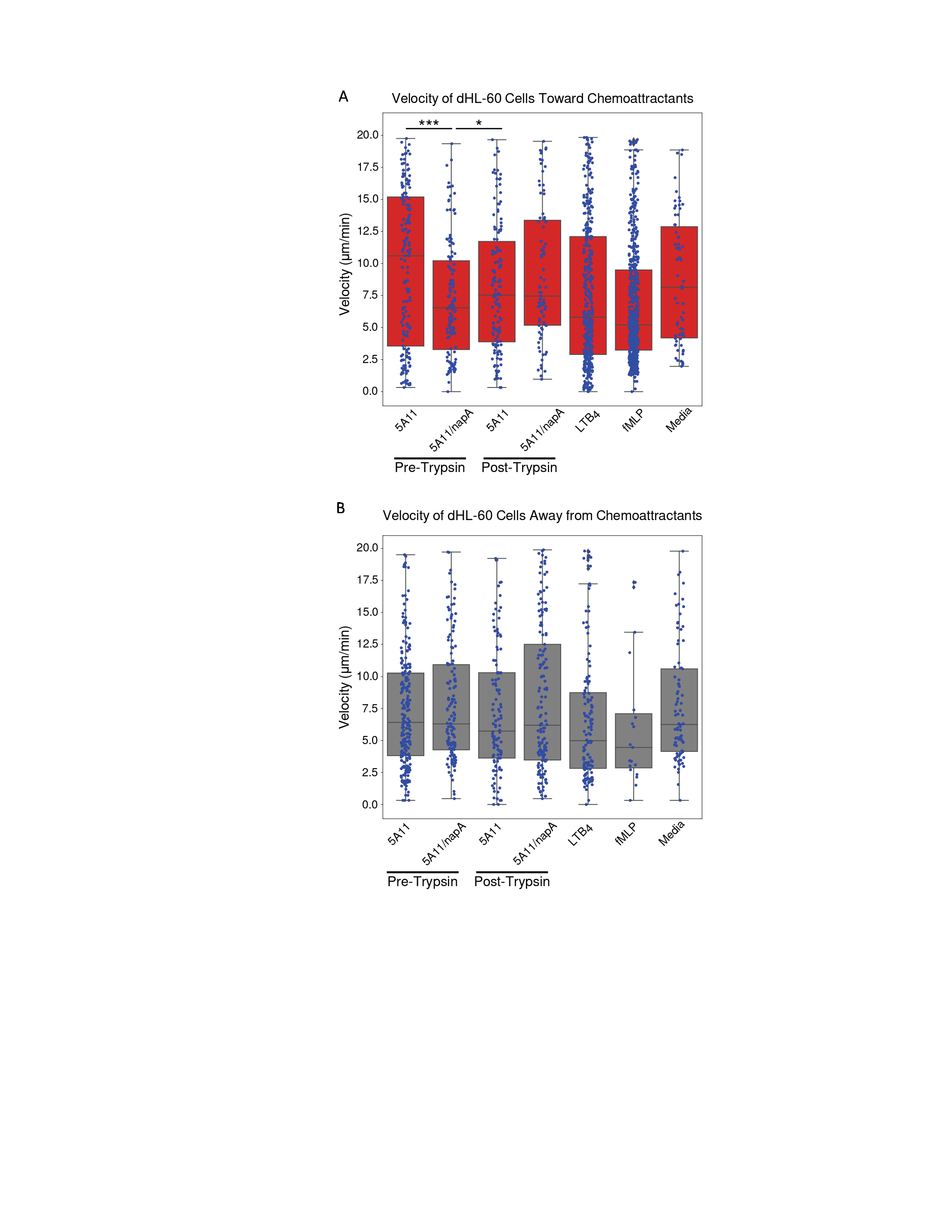

Supplement: S4 Fig — Single cell velocity values are plotted over a box plot showing range of values. (A) Cells migrating toward PG bait samples and chemoattractants show Nap-A associated PG has a similar velocity (10.94 ± 4.79 μm/min) to known chemoattractants LTB4 (7.04 ± 4.90 μm/min) and fMLP (6.93 ± 4.40 μm/min). (B) Cells migrating away from PG bait samples and chemoattractants show similar velocities. To evaluate differences between responses ANOVA were performed with Turkey’s correction for multiple comparisons (* = p < 0.05, *** = p < 0.001). (TIFF) [file ppat.1009546.s009.tiff]

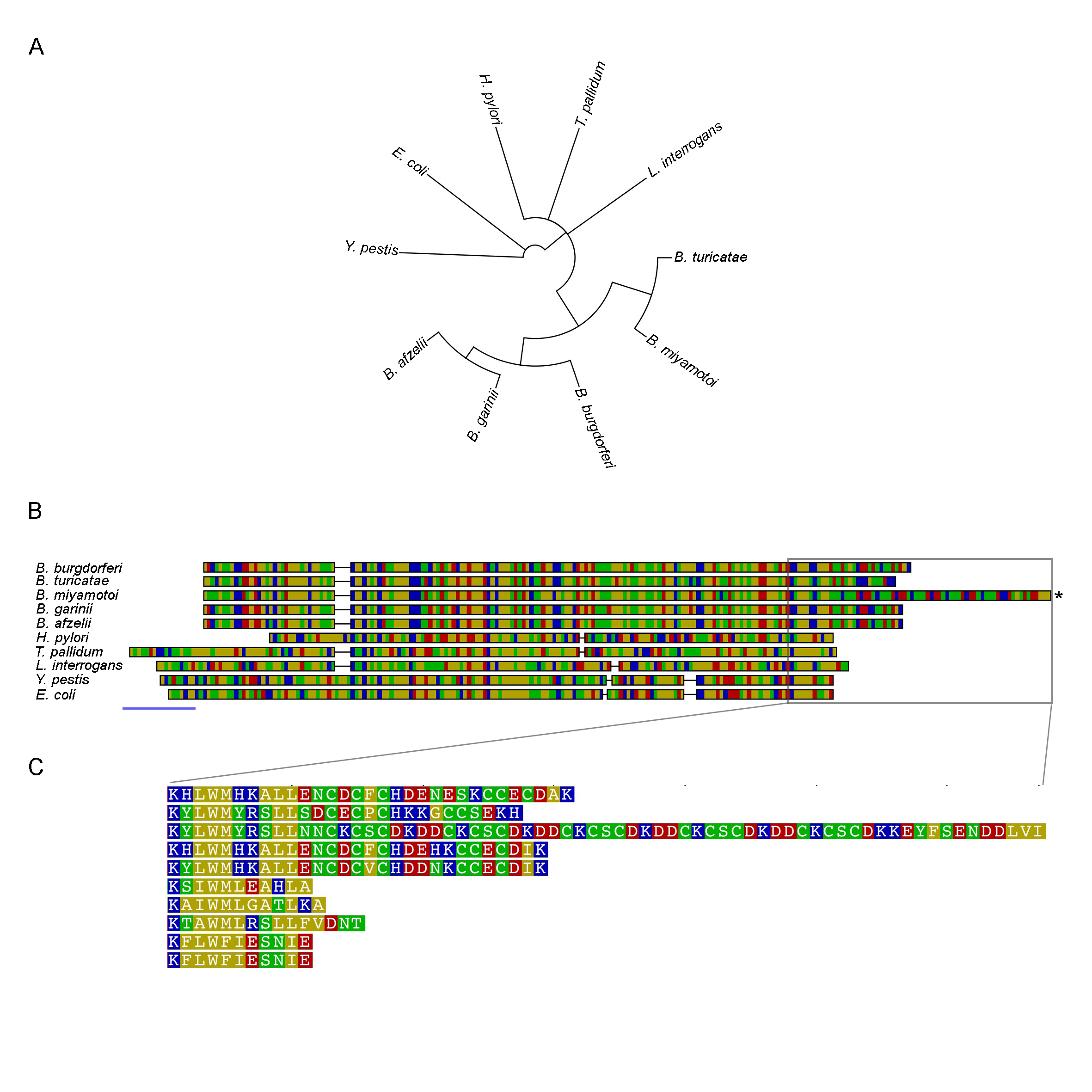

Supplement: S5 Fig — (A) Phylogenic analysis of Dps/NapA homologues in Borreliae, Helicobacter pylori, Treponema pallidum, Leptospira interrogans, Yersinia pestis, and Escherichia coli. (B) Amino acid alignment of Dps/NapA homologues from bacteria in A. The Lysine-rich DNA binding domain is underlined (blue) (C) Zoomed in amino acid sequence of the C-terminus of Dps/NapA homologues. (TIF) [file ppat.1009546.s010.tif]
