# Supplementary material for: The peptidoglycan-associated protein NapA plays an important role in the envelope integrity and in the pathogenesis of the lyme disease spirochete
Source: PLoS Pathog. 2021 May 13;17(5):e1009546. doi: 10.1371/journal.ppat.1009546 (PMC8118282; doi:10.1371/journal.ppat.1009546)
Supplement: S1 Text — (DOCX) [file ppat.1009546.s018.docx]

# MATLAB Scripts

The provided codes require previous actions before execution. However, the description of the script should indicate the needed steps. Note that these scripts are all post-image analyses done with the data from Oufti.

The provided codes require previous actions before execution. However, the description of the script should indicate the needed steps. Note that these scripts are all post-image analyses done with the data from Oufti.

**AB_GatherDataForViolinPlot.** This code takes the signal analysis generated from Oufti and gathers the averaged signal intensity for each cell for the entire population and places it in a list. This data was then plotted via GraphPad Prism.

%%

% This code takes the information and variables produced from Oufti and uses

% the function "ab_read". This code will gather the average signal data per each

% cell for the entire poplulation into a single array to be used for data analysis,

% under the variable "PopulationSIgnalAverage".

ab_read

signallength=0;

d=[];

for a=1:length(signals)

b=signals{1,a};

signallength(end+1)=length(b.signal2);

end

maxlen=max(signallength);

%%

for a=1:length(signals)

b=signals{1,a};

c=mean(b.signal2);

d(:,a)=c;

end

d=d(:);

d=sort(d,'descend');

d=d(~isnan(d));

PopulationSignalAverage{:,1}=d;

clear a b c d maxlen signals

**AB_PlotExtractDataNorm.** This code takes the information and variables produced from “AB_SpotRelativePlacement_SpotStrength” and creates bins of the data. It then plots the average signal intensity for that bin on top of the graph that was created from AB_Signal1relative_Signal1signal. Finally, it takes that binned information and plots a shaded standard deviation for those averages using the function shadedErrorBar.m.

%%

% This function was designed to take the data points from a graph and create

% a binned collection of the data and plot it against a standard deviation. For

% this paper it uses the data generated from code "AB_SpotRelativePlacement_SpotStrength".

%

% The two data sets should be saved under the variables "sortedXYnorm" and

% "sortedXYnorm2" (generated normally from "AB_SpotRelativePlacement_SpotStrength").

hold on

plot(sortedXYnorm(1,:),sortedXYnorm(2,:),".","Color",[.7,.7,.7])

xlabel("Relative cell (or sacculi) length")

ylabel("Signal intensity (au)")

StandDev=[];

KK=[];

binsize=0.05;

for a=[0:binsize:1]

if a == 0

[~,jj]=find(sortedXYnorm(1,:)>(0) & sortedXYnorm(1,:)<binsize);

kk=mean(sortedXYnorm(2,(jj(1):jj(end))));

KK=[KK,kk];

plot(a,kk,".r","LineWidth",10)

standDev=std(sortedXYnorm(2,(jj(1):jj(end))));

StandDev=[StandDev,standDev];

elseif a == binsize

[~,jj]=find(sortedXYnorm(1,:)>(binsize) & sortedXYnorm(1,:)<a+binsize);

kk=mean(sortedXYnorm(2,(jj(1):jj(end))));

KK=[KK,kk];

plot(a,kk,".r","LineWidth",10)

standDev=std(sortedXYnorm(2,(jj(1):jj(end))));

StandDev=[StandDev,standDev];

elseif a == 1

else

[~,jj]=find(sortedXYnorm(1,:)>(a) & sortedXYnorm(1,:)<a+binsize);

kk=mean(sortedXYnorm(2,(jj(1):jj(end))));

KK=[KK,kk];

plot(a,kk,".r","LineWidth",10)

standDev=std(sortedXYnorm(2,(jj(1):jj(end))));

StandDev=[StandDev,standDev];

end

end

shadedErrorBar([(binsize/2):binsize:1-(binsize/2)],KK,StandDev)

%%

hold on

plot(sortedXYnorm2(1,:),sortedXYnorm2(2,:),".","Color",[.91,.47,.47])

xlabel("Relative cell (or sacculi) length")

ylabel("Signal intensity (au)")

StandDev=[];

KK=[];

binsize=0.05;

for a=[0:binsize:1]

if a == 0

[~,jj]=find(sortedXYnorm2(1,:)>(0) & sortedXYnorm2(1,:)<binsize);

kk=mean(sortedXYnorm2(2,(jj(1):jj(end))));

KK=[KK,kk];

plot(a,kk,".r","LineWidth",10)

standDev=std(sortedXYnorm2(2,(jj(1):jj(end))));

StandDev=[StandDev,standDev];

elseif a == binsize

[~,jj]=find(sortedXYnorm2(1,:)>(binsize) & sortedXYnorm2(1,:)<a+binsize);

kk=mean(sortedXYnorm2(2,(jj(1):jj(end))));

KK=[KK,kk];

plot(a,kk,".r","LineWidth",10)

standDev=std(sortedXYnorm2(2,(jj(1):jj(end))));

StandDev=[StandDev,standDev];

elseif a == 1

else

[~,jj]=find(sortedXYnorm2(1,:)>(a) & sortedXYnorm2(1,:)<a+binsize);

kk=mean(sortedXYnorm2(2,(jj(1):jj(end))));

KK=[KK,kk];

plot(a,kk,".r","LineWidth",10)

standDev=std(sortedXYnorm2(2,(jj(1):jj(end))));

StandDev=[StandDev,standDev];

end

end

shadedErrorBar([(binsize/2):binsize:1-(binsize/2)],KK,StandDev)

hold off

**ab_read.** This code takes the data generated from Oufti and reformats the data into a single cell array named “signals” to be used.

d=0;

for a=1:length(cellList.meshData)

for b=1:length(cellList.meshData{1,a})

c=cellList.meshData{1,a}{1,b};

d=d+1;

signals{1,d}.index=[1,d];

signals{1,d}.cellLength=((length(c.mesh))-1);

if isfield(c,'signal0')

signals{1,d}.signal0=c.signal0;

end

if isfield(c,'signal1')

signals{1,d}.signal1=c.signal1;

end

if isfield(c,'signal2')

signals{1,d}.signal2=c.signal2;

end

end

end

clear a ans b c d

**AB_SpotRelativePlacement_SpotStrength.** This code takes the analysis produced by Oufti and creates a graph that represents the relative NapA position in the cell compared to the NapA signal intensity.

%%

% Signal1 Spots relative Placement in the cell vs Signal1 signal intensity

%

% you need to have done the spot detection on Oufti

%

% This function was created to take the spots from spot analysis in Oufti. The

% cellList and plot their normalized position compared to the actual signal intensity.

% This function was designed to evaluate signal position bias via general signal

% intensity.

%

% To have normalized signal binning and standard deviation against two plots,

% run the same code with the second data set. After finishing, run "AB_PlotExtractDataNorm".

for a=1:numel(cellList.meshData)

for b=1:numel(cellList.meshData{1,a})

c=cellList.meshData{1,a}{1,b};

if isempty(c.spots.l)

else

if isfield(c,"signal1")

length=numel(c.signal1);

NapA=c.signal1;

NapANorm=NapA/(max(NapA));

spots=c.spots.l;

spots(spots>length)=[];

spots(spots<0)=[];

spotsRound=round(spots);

spotsNorm=spots./length;

hold on

plot(spotsNorm,NapA(spotsRound),".","color",[.7,.7,.7])

else

end

end

end

end

hold off

xlabel("Relative NapA position")

ylabel("NapA signal (pixels)")

d=findobj(gca,"Type","line");

x=get(d(1:end),"XData");

y=get(d(1:end),"YData");

X=[x{1:end,1}];

Y=[y{1:end,1}];

Ynorm=(Y/(max(Y)));

XY=[X;Y];

XYnorm=[X;Y];

[sortedX,ii]=sort(X);

sortedY=Y(ii);

sortedYnorm=Ynorm(ii);

sortedXY=[sortedX;sortedY];

if exist("sortedXYnorm") == 1

sortedXYnorm2=[sortedX;sortedYnorm];

else

sortedXYnorm=[sortedX;sortedYnorm];

end

sortedXYnorm=[sortedX;sortedYnorm];

[N,edges]=histcounts(X);

**demograph.** This function will take the signal analysis from Oufti and create a graphical representation of the population based on size.

function demograph(varargin) %cellList,maxCellNum,maxCellLength,numPixelsMovingAverage,signal,frameNum,descriptor,conversionFactor

%-----------------------------------------------------------------------------------------------------

%-----------------------------------------------------------------------------------------------------

%function demograph(varargin)

%

%@author: Jason Hocking

%@date: October 27, 2011

%@modified: Ahmad Paintdakhi -- August 1, 2013

%@copyright 2011-2013 Yale University

%====================================================================================================

%**********output********:

%No output arguements required, the function oly plots information.

%**********Input********:

%cellList: cellList structure

%maxCellNum: maximum number of cells to be included for final demograph

%maxCellLength: maximum length of cell

%numPixelsMovingAverage: number of pixels to be used for the moving average. This

% routine finds the segment where max intensity of the signal

% is located.

%signal: an array for signal information. For example, to use only signal 1 the

% array should be [1,0], for signal 2 --> [0,1] and both signal 1 and

% signal 2 ---> [1,1].

%frameNum: frame # to be used for analysis or [] vector (use all frames in

% a dataset).

%

%descriptor: The descriptor value is a key for the type of demograph to be drawn.

% The different keys are 'randomN','randomNOriented', 'constriction_noNormalization',

% 'sort_by_constriction','constriction','normByPopulation', and 'normByPopulationOriented'.

%conversionFactor: Pixel to micron conversion factor.

%Purpose: script was designed to provide a colormap of relative segment intensities for every

% cell in an asynchorous cellList, sorted by cell length in ascending order.

%====================================================================================================

if length(varargin) < 8 || length(varargin) > 8

disp('A total of 8 arguments are accepted');

return;

end

if ~isstruct(varargin{1}) && ~iscell(varargin{1})

disp('cellList must be a struct or cell array')

return;

end

if length(varargin{5}) ~=2

disp('signal should be a vector of length 2, such as [1,0] or [0,1] or [1,1]')

return;

end

if ~ischar(varargin{7})

disp(['descriptor needs to be a string such as ' 'randomN'])

return;

end

if ~isscalar(varargin{8})

disp('Conversion factor needs to be a scalar value such as 0.064')

return;

end

cellList = varargin{1};

maxCellNum = varargin{2};

maxCellLength = varargin{3};

numPixelsMovingAverage = varargin{4};

signal = varargin{5};

frameNum = varargin{6};

descriptor = varargin{7};

conversionFactor = varargin{8};

warning('off','MATLAB:colon:nonIntegerIndex');

descriptorValues = {'randomN'

'randomNOriented'

'constriction_no_normalization'

'sort_by_constriction'

'constriction'};

%---------------------------------------------------------------------------------

%if cellList is in old format convert to new and if already in new format

%add extra fields and make fields as double class for mathematical calculations.

if signal(1) == 1 && sum(signal) == 1

signalInfo = 'signal1';

elseif signal(2) == 1 && sum(signal) == 1

signalInfo = 'signal2';

elseif sum(signal) == 2

signalInfo = 'signal1';

end

if ~isfield(cellList,'meshData')

cellList = oufti_makeNewCellListFromOld(cellList);

try

try

for ii = 1:length(cellList.meshData)

for jj = 1:length(cellList.meshData{ii})

cellList.meshData{ii}{jj} = getextradata(cellList.meshData{ii}{jj});

end

end

catch err

disp(err);

disp('Add extra fields in batch mode');

end

catch err %#ok

disp('check that cellList is not empty')

return;

end

else

cellList = oufti_makeCellListDouble(cellList);

try

try

for ii = 1:length(cellList.meshData)

for jj = 1:length(cellList.meshData{ii})

cellList.meshData{ii}{jj} = getextradata(cellList.meshData{ii}{jj});

end

end

catch err

disp(err);

disp('Add extra fields in batch mode');

end

catch err %#ok

disp('check that cellList is not empty')

return;

end

end

%---------------------------------------------------------------------------------

if isempty(frameNum)

frameList = 1:length(cellList.meshData);

else

frameList = frameNum;

end

switch descriptor

case 'randomNOriented'

try

replacement=false;

%%finds the maximum number of stepareas inside of a cell from the cellList

maxsizelarray=[];

n=0;

for frame = frameList

for cellNum = 1:length(cellList.meshData{frame})

if isempty(cellList.meshData{frame}{cellNum}) || ...

length(cellList.meshData{frame}{cellNum}.mesh)<4 ...

||~isfield(cellList.meshData{frame}{cellNum},signalInfo) ...

|| eval('isempty(cellList.meshData{frame}{cellNum}.(signalInfo))') ...

|| cellList.meshData{frame}{cellNum}.length>maxCellLength

continue

end

n=n+1;

end

end

if n<=maxCellNum

maxCellNum=n;

end

rand=randsample(n,maxCellNum,replacement);

n=0;

for frame = frameList

for cellNum = 1:length(cellList.meshData{frame})

if isempty(cellList.meshData{frame}{cellNum}) ...

|| length(cellList.meshData{frame}{cellNum}.mesh)<4 ...

|| ~isfield(cellList.meshData{frame}{cellNum},signalInfo) ...

|| eval('isempty(cellList.meshData{frame}{cellNum}.(signalInfo))') ...

|| cellList.meshData{frame}{cellNum}.length>maxCellLength

continue

end

n = n+1;

b=rand==n;

if sum(b)~=1

continue

end

maxsizelarray=[maxsizelarray length(cellList.meshData{frame}{cellNum}.lengthvector)];%#ok<AGROW>

end

end

if isempty(maxsizelarray)

warndlg(['No field ' signalInfo ' recorded for this cell: Use Reuse meshes toggle button to compute ' signalInfo]);

return;

end

%using the maxima from above, a matrix consiting of zeros is created to be

%filled in by mesh intensities

relintarray1=zeros(max(maxsizelarray),maxCellNum);

maxsizel=max(maxsizelarray);

if maxCellLength > maxsizel;

maxCellLength = maxsizel;

end

maxsizel2 = ceil(maxsizel); if mod(maxsizel2,2)==0, maxsizel2=maxsizel2+1; end

maxsizel2a = maxsizel2/2+0.5;

n=0;

passed=0;

cellLength=[];

%zeroarray is replaced with relative segment intensity data from the cell

for frame = frameList

for cellNum = 1:length(cellList.meshData{frame})

place=1;%#ok

if isempty(cellList.meshData{frame}{cellNum}) ...

|| length(cellList.meshData{frame}{cellNum}.mesh)<4 ...

||~isfield(cellList.meshData{frame}{cellNum},signalInfo) ...

|| eval('isempty(cellList.meshData{frame}{cellNum}.(signalInfo))') ...

|| cellList.meshData{frame}{cellNum}.length>maxCellLength

continue

end

n = n+1;

b=rand==n;

if sum(b)~=1

continue

end

passed=passed+1;

if signal(1) == 1 && sum(signal) == 1

%%calculates the fluorescent intensities in each segment normalized

%%by the area of that segment

if length(cellList.meshData{frame}{cellNum}.signal1) > length(cellList.meshData{frame}{cellNum}.steparea)

cellList.meshData{frame}{cellNum}.relsignal1 = (cellList.meshData{frame}{cellNum}.signal1(1:length(cellList.meshData{frame}{cellNum}.steparea))./cellList.meshData{frame}{cellNum}.steparea);

else

cellList.meshData{frame}{cellNum}.relsignal1 = (cellList.meshData{frame}{cellNum}.signal1./cellList.meshData{frame}{cellNum}.steparea(1:length(cellList.meshData{frame}{cellNum}.signal1)));

end

%%segments are then normalized to the brightest segment so that

%%this sigment is represented as 1.

cellList.meshData{frame}{cellNum}.relint1 = (cellList.meshData{frame}{cellNum}.relsignal1./max(cellList.meshData{frame}{cellNum}.relsignal1));

elseif signal(2) == 1 && sum(signal) == 1

%%calculates the fluorescent intensities in each segment normalized

%%by the area of that segment

if length(cellList.meshData{frame}{cellNum}.signal2) > length(cellList.meshData{frame}{cellNum}.steparea)

cellList.meshData{frame}{cellNum}.relsignal2 = (cellList.meshData{frame}{cellNum}.signal2(1:length(cellList.meshData{frame}{cellNum}.steparea))./cellList.meshData{frame}{cellNum}.steparea);

else

cellList.meshData{frame}{cellNum}.relsignal2 = (cellList.meshData{frame}{cellNum}.signal2./cellList.meshData{frame}{cellNum}.steparea(1:length(cellList.meshData{frame}{cellNum}.signal2)));

end

%%segments are then normalized to the brightest segment so that

%%this sigment is represented as 1.

cellList.meshData{frame}{cellNum}.relint2 = (cellList.meshData{frame}{cellNum}.relsignal2./max(cellList.meshData{frame}{cellNum}.relsignal2));

elseif sum(signal) == 2

%%calculates the fluorescent intensities in each segment normalized

%%by the area of that segment

if length(cellList.meshData{frame}{cellNum}.signal1) > length(cellList.meshData{frame}{cellNum}.steparea)

cellList.meshData{frame}{cellNum}.relsignal1 = (cellList.meshData{frame}{cellNum}.signal1(1:length(cellList.meshData{frame}{cellNum}.steparea))./cellList.meshData{frame}{cellNum}.steparea);

else

cellList.meshData{frame}{cellNum}.relsignal1 = (cellList.meshData{frame}{cellNum}.signal1./cellList.meshData{frame}{cellNum}.steparea(1:length(cellList.meshData{frame}{cellNum}.signal1)));

end

%%segments are then normalized to the brightest segment so that

%%this sigment is represented as 1.

cellList.meshData{frame}{cellNum}.relint1 = (cellList.meshData{frame}{cellNum}.relsignal1./max(cellList.meshData{frame}{cellNum}.relsignal1));

%%calculates the fluorescent intensities in each segment normalized

%%by the area of that segment

if length(cellList.meshData{frame}{cellNum}.signal2) > length(cellList.meshData{frame}{cellNum}.steparea)

cellList.meshData{frame}{cellNum}.relsignal2 = (cellList.meshData{frame}{cellNum}.signal2(1:length(cellList.meshData{frame}{cellNum}.steparea))./cellList.meshData{frame}{cellNum}.steparea);

else

cellList.meshData{frame}{cellNum}.relsignal2 = (cellList.meshData{frame}{cellNum}.signal2./cellList.meshData{frame}{cellNum}.steparea(1:length(cellList.meshData{frame}{cellNum}.signal2)));

end

%%segments are then normalized to the brightest segment so that

%%this sigment is represented as 1.

cellList.meshData{frame}{cellNum}.relint2 = (cellList.meshData{frame}{cellNum}.relsignal2./max(cellList.meshData{frame}{cellNum}.relsignal2));

else

disp('provide information in signal variable')

return;

end

%%% A MOVING AVERAGE IS CALCULATED FOR EACH OF THE SEGMENTS TO FIND THE SINGLE BRIGHTEST SEGMENT AREA

cellList.meshData{frame}{cellNum}.meshavg=[];

if signal(1) == 1 && sum(signal) == 1

for place = 1:(length(cellList.meshData{frame}{cellNum}.relint1)-(numPixelsMovingAverage-1));

cellList.meshData{frame}{cellNum}.meshavg=[cellList.meshData{frame}{cellNum}.meshavg mean(cellList.meshData{frame}{cellNum}.relint1(place:(place+(numPixelsMovingAverage-1))))];

place=place+1;%#ok

end

elseif signal(2) == 1 && sum(signal) == 1

for place = 1:(length(cellList.meshData{frame}{cellNum}.relint2)-(numPixelsMovingAverage-1));

cellList.meshData{frame}{cellNum}.meshavg=[cellList.meshData{frame}{cellNum}.meshavg mean(cellList.meshData{frame}{cellNum}.relint2(place:(place+(numPixelsMovingAverage-1))))];

place=place+1; %#ok

end

elseif sum(signal) == 2

for place = 1:(length(cellList.meshData{frame}{cellNum}.relint2)-(numPixelsMovingAverage-1));

cellList.meshData{frame}{cellNum}.meshavg=[cellList.meshData{frame}{cellNum}.meshavg mean(cellList.meshData{frame}{cellNum}.relint2(place:(place+(numPixelsMovingAverage-1))))];

place=place+1;%#ok

end

end

%%WITH THE BRIGHTEST SEGMENT CALCULATED ABOVE WE CAN ORIENT THE

%%CELL SO THAT THE BRIGHTEST SEGMENT IS ON THE RIGHTS (i.e. WITH FtsZ BEING POLAR ON RIGHT(NEW POLE)

%%AND LARGER STALK CELL BIAS LETTING THE FtsZ RING BE ON THE RIGHT

%%AS WELL)

[~,maxavg]=max(cellList.meshData{frame}{cellNum}.meshavg);

if maxavg<=length(cellList.meshData{frame}{cellNum}.meshavg)/2+1;

if signal(1) ==1 && sum(signal) == 1

cellList.meshData{frame}{cellNum}.relint1=flipud(cellList.meshData{frame}{cellNum}.relint1);

elseif signal(2) == 1 && sum(signal) == 1

cellList.meshData{frame}{cellNum}.relint2=flipud(cellList.meshData{frame}{cellNum}.relint2);

elseif sum(signal) == 2

cellList.meshData{frame}{cellNum}.relint1=flipud(cellList.meshData{frame}{cellNum}.relint1);

cellList.meshData{frame}{cellNum}.relint2=flipud(cellList.meshData{frame}{cellNum}.relint2);

end

end

k = floor(cellList.meshData{frame}{cellNum}.length/2);

temp = cellList.meshData{frame}{cellNum}.lengthvector-cellList.meshData{frame}{cellNum}.length/2;

if signal(1) ==1 && sum(signal) == 1

interpint1 = interp1(temp(1:length(cellList.meshData{frame}{cellNum}.relint1)),cellList.meshData{frame}{cellNum}.relint1,-k:k,'linear','extrap');

relintarray1(maxsizel2a-k:maxsizel2a+k,passed)=interpint1;

elseif signal(2) == 1 && sum(signal) == 1

interpint2 = interp1(temp(1:length(cellList.meshData{frame}{cellNum}.relint2)),cellList.meshData{frame}{cellNum}.relint2,-k:k,'linear','extrap');

relintarray2(maxsizel2a-k:maxsizel2a+k,passed)=interpint2;%#ok

elseif sum(signal) == 2

interpint1 = interp1(temp(1:length(cellList.meshData{frame}{cellNum}.relint1)),cellList.meshData{frame}{cellNum}.relint1,-k:k,'linear','extrap');

relintarray1(maxsizel2a-k:maxsizel2a+k,passed)=interpint1;

interpint2 = interp1(temp(1:length(cellList.meshData{frame}{cellNum}.relint2)),cellList.meshData{frame}{cellNum}.relint2,-k:k,'linear','extrap');

relintarray2(maxsizel2a-k:maxsizel2a+k,passed)=interpint2;%#ok

end

cellLength=[cellLength cellList.meshData{frame}{cellNum}.length];%#ok

end

end

% % cells length array is concatonated with the fluorescence matrix. This matrix is then sorted by length in ascending order

numlist = [1:1:maxCellNum];%#ok

lvint0=cat(2,numlist',cellLength');

if signal(1) ==1 && sum(signal) == 1

lvint1=cat(2,lvint0,relintarray1');

lnumsort1=sortrows(lvint1,[2]);%#ok

elseif signal(2) == 1 && sum(signal) == 1

lvint2=cat(2,lvint0,relintarray2');

lnumsort2=sortrows(lvint2,[2]);%#ok

elseif sum(signal) == 2

lvint1=cat(2,lvint0,relintarray1');

lvint2=cat(2,lvint0,relintarray2');

lnumsort1=sortrows(lvint1,[2]);%#ok

lnumsort2=sortrows(lvint2,[2]);%#ok

end

if signal(1) ==1 && sum(signal) == 1

%relative intensities are plotted accoring to a colormap

x=[-conversionFactor*maxCellLength./2 conversionFactor*maxCellLength./2];

x = repmat(x(1):x(2)*2/(size(lnumsort1,2)-3):x(2),size(lnumsort1,1),1);

y = repmat((1:size(lnumsort1,1)),size(lnumsort1,2)-2,1)';

dataToPlot = lnumsort1(1:end,3:end);

dataToPlot(dataToPlot==0) = NaN;

pcolor(x,y,flipud(dataToPlot)); colormap jet; colorbar;caxis([0 1]);shading flat;

xlabel('Distance From Midcell (\mum)','FontSize',18)

ylabel('Number of Cells','FontSize',18)

elseif signal(2) == 1 && sum(signal) == 1

%relative intensities are plotted accoring to a colormap

x=[-conversionFactor*maxCellLength./2 conversionFactor*maxCellLength./2];

x = repmat(x(1):x(2)*2/(size(lnumsort2,2)-3):x(2),size(lnumsort2,1),1);

y = repmat((1:size(lnumsort2,1)),size(lnumsort2,2)-2,1)';

dataToPlot = lnumsort2(1:end,3:end);

dataToPlot(dataToPlot==0) = NaN;

pcolor(x,y,flipud(dataToPlot)); colormap jet; colorbar;caxis([0 1]);shading flat;

xlabel('Distance From Midcell (\mum)','FontSize',18)

ylabel('Number of Cells','FontSize',18)

elseif sum(signal) == 2

%relative intensities are plotted accoring to a colormap

x=[-conversionFactor*maxCellLength./2 conversionFactor*maxCellLength./2];

x = repmat(x(1):x(2)*2/(size(lnumsort1,2)-3):x(2),size(lnumsort1,1),1);

y = repmat((1:size(lnumsort1,1)),size(lnumsort1,2)-2,1)';

dataToPlot = lnumsort1(1:end,3:end);

dataToPlot(dataToPlot==0) = NaN;

pcolor(x,y,flipud(dataToPlot)); colormap jet; colorbar;caxis([0 1]);shading flat;

xlabel('Distance From Midcell (\mum)','FontSize',18)

ylabel('Number of Cells','FontSize',18)

%relative intensities are plotted accoring to a colormap

figure;

x=[-conversionFactor*maxCellLength./2 conversionFactor*maxCellLength./2];

x = repmat(x(1):x(2)*2/(size(lnumsort2,2)-3):x(2),size(lnumsort2,1),1);

y = repmat((1:size(lnumsort2,1)),size(lnumsort2,2)-2,1)';

dataToPlot = lnumsort2(1:end,3:end);

dataToPlot(dataToPlot==0) = NaN;

pcolor(x,y,flipud(dataToPlot)); colormap jet; colorbar;caxis([0 1]);shading flat;

xlabel('Distance From Midcell (\mum)','FontSize',18)

ylabel('Number of Cells','FontSize',18)

end

catch err

if strcmpi(err.identifier,'MATLAB:catenate:dimensionMismatch')

warndlg('Choose a smaller number for max cell number parameter');

return;

end

end

case 'normByPopulationOriented'

replacement=false;

intensityVector1Min = [];

intensityVector1Max = [];

intensityVector2Min = [];

intensityVector2Max = [];

%%finds the maximum number of stepareas inside of a cell from the cellList

maxsizelarray=[];

n=0;

for frame = frameList

for cellNum = 1:length(cellList.meshData{frame})

if isempty(cellList.meshData{frame}{cellNum}) || ~isfield(cellList.meshData{frame}{cellNum},'mesh')||length(cellList.meshData{frame}{cellNum}.mesh)<4 ...

||~isfield(cellList.meshData{frame}{cellNum},signalInfo) ...

|| eval('isempty(cellList.meshData{frame}{cellNum}.(signalInfo))') || cellList.meshData{frame}{cellNum}.length>maxCellLength

continue

end

n=n+1;

end

end

if n<=maxCellNum

maxCellNum=n;

end

rand=randsample(n,maxCellNum,replacement);

n=0;

for frame = frameList

for cellNum = 1:length(cellList.meshData{frame})

if isempty(cellList.meshData{frame}{cellNum}) ...

|| length(cellList.meshData{frame}{cellNum}.mesh)<4 ...

|| ~isfield(cellList.meshData{frame}{cellNum},signalInfo) ...

|| eval('isempty(cellList.meshData{frame}{cellNum}.(signalInfo))') ...

|| cellList.meshData{frame}{cellNum}.length>maxCellLength

continue

end

n = n+1;

b=rand==n;

if sum(b)~=1

continue

end

maxsizelarray=[maxsizelarray length(cellList.meshData{frame}{cellNum}.lengthvector)];%#ok<AGROW>

%fix signal info

try

%cellList.meshData{frame}{cellNum}.signal1 = (cellList.meshData{frame}{cellNum}.signal1(1:length(cellList.meshData{frame}{cellNum}.steparea))./cellList.meshData{frame}{cellNum}.steparea);

cellList.meshData{frame}{cellNum}.signal1 = (cellList.meshData{frame}{cellNum}.signal1(1:length(cellList.meshData{frame}{cellNum}.steparea))./cellList.meshData{frame}{cellNum}.steparea);

cellList.meshData{frame}{cellNum}.signal1 = cellList.meshData{frame}{cellNum}.signal1./sum(cellList.meshData{frame}{cellNum}.signal1);

intensityVector1Min=[intensityVector1Min min(cellList.meshData{frame}{cellNum}.signal1)];%#ok<AGROW>

intensityVector1Max=[intensityVector1Max max(cellList.meshData{frame}{cellNum}.signal1)];%#ok<AGROW>

catch

end

try

intensityVector2Min=[intensityVector2Min min(cellList.meshData{frame}{cellNum}.signal2)];%#ok<AGROW>

intensityVector2Max=[intensityVector2Max max(cellList.meshData{frame}{cellNum}.signal2)];%#ok<AGROW>

catch

end

end

end

%using the maxima from above, a matrix consiting of zeros is created to be

%filled in by mesh intensities

relintarray1=zeros(max(maxsizelarray),maxCellNum);

maxsizel=max(maxsizelarray);

try

smallValueIntensityVector1Min = quantile(intensityVector1Min(intensityVector1Min~=0),0.3);

largeValueIntensityVector1Max = quantile(intensityVector1Max(intensityVector1Max~=0),0.9);

catch

end

try

smallValueIntensityVector2Min = quantile(intensityVector2Min(intensityVector2Min~=0),0.3);

largeValueIntensityVector2Max = quantile(intensityVector2Max(intensityVector2Max~=0),0.9);

catch

end

% if maxCellLength > maxsizel;

% maxsizel=maxCellLength;

% end

maxsizel2 = ceil(maxsizel); if mod(maxsizel2,2)==0, maxsizel2=maxsizel2+1; end

maxsizel2a = maxsizel2/2+0.5;

n=0;

passed=0;

cellLength=[];

%zeroarray is replaced with relative segment intensity data from the cell

for frame = frameList

for cellNum = 1:length(cellList.meshData{frame})

place=1;%#ok

if isempty(cellList.meshData{frame}{cellNum}) || length(cellList.meshData{frame}{cellNum}.mesh)<4 ...

||~isfield(cellList.meshData{frame}{cellNum},'signal1') ...

|| isempty(cellList.meshData{frame}{cellNum}.signal1) || cellList.meshData{frame}{cellNum}.length>maxCellLength

continue

end

n = n+1;

b=rand==n;

if sum(b)~=1

continue

end

passed=passed+1;

if signal(1) == 1 && sum(signal) == 1

%%calculates the fluorescent intensities in each segment normalized

%%by the area of that segment

cellList.meshData{frame}{cellNum}.relsignal1 = (cellList.meshData{frame}{cellNum}.signal1 - smallValueIntensityVector1Min)./(largeValueIntensityVector1Max - smallValueIntensityVector1Min);

cellList.meshData{frame}{cellNum}.relint1 = cellList.meshData{frame}{cellNum}.relsignal1;

%%segments are then normalized to the brightest segment so that

%%this sigment is represented as 1.

% % % cellList.meshData{frame}{cellNum}.relint1 = (cellList.meshData{frame}{cellNum}.relsignal1./max(cellList.meshData{frame}{cellNum}.relsignal1));

elseif signal(2) == 1 && sum(signal) == 1

%%calculates the fluorescent intensities in each segment normalized

%%by the area of that segment

cellList.meshData{frame}{cellNum}.relsignal2 = (cellList.meshData{frame}{cellNum}.signal2 - smallValueIntensityVector2Min)./(largeValueIntensityVector2Max - smallValueIntensityVector2Min);

cellList.meshData{frame}{cellNum}.relint2 = cellList.meshData{frame}{cellNum}.relsignal2;

%%segments are then normalized to the brightest segment so that

%%this sigment is represented as 1.

% % % cellList.meshData{frame}{cellNum}.relint2 = (cellList.meshData{frame}{cellNum}.relsignal2./max(cellList.meshData{frame}{cellNum}.relsignal2));

elseif sum(signal) == 2

%%calculates the fluorescent intensities in each segment normalized

%%by the area of that segment

cellList.meshData{frame}{cellNum}.relsignal1 = (cellList.meshData{frame}{cellNum}.signal1 - smallValueIntensityVector1Min)./(largeValueIntensityVector1Max - smallValueIntensityVector1Min);

cellList.meshData{frame}{cellNum}.relint1 = cellList.meshData{frame}{cellNum}.relsignal1;

%%segments are then normalized to the brightest segment so that

%%this sigment is represented as 1.

% % % cellList.meshData{frame}{cellNum}.relint1 = (cellList.meshData{frame}{cellNum}.relsignal1./max(cellList.meshData{frame}{cellNum}.relsignal1));

%%calculates the fluorescent intensities in each segment normalized

%%by the area of that segment

cellList.meshData{frame}{cellNum}.relsignal2 = (cellList.meshData{frame}{cellNum}.signal2 - smallValueIntensityVector2Min)./(largeValueIntensityVector2Max - smallValueIntensityVector2Min);

cellList.meshData{frame}{cellNum}.relint2 = cellList.meshData{frame}{cellNum}.relsignal2;

%%segments are then normalized to the brightest segment so that

%%this sigment is represented as 1.

% % % cellList.meshData{frame}{cellNum}.relint2 = (cellList.meshData{frame}{cellNum}.relsignal2./max(cellList.meshData{frame}{cellNum}.relsignal2));

else

disp('provide information in signal variable')

return;

end

%%% A MOVING AVERAGE IS CALCULATED FOR EACH OF THE SEGMENTS TO FIND THE SINGLE BRIGHTEST SEGMENT AREA

cellList.meshData{frame}{cellNum}.meshavg=[];

if signal(1) == 1 && sum(signal) == 1

for place = 1:(length(cellList.meshData{frame}{cellNum}.relint1)-(numPixelsMovingAverage-1));

cellList.meshData{frame}{cellNum}.meshavg=[cellList.meshData{frame}{cellNum}.meshavg mean(cellList.meshData{frame}{cellNum}.relint1(place:(place+(numPixelsMovingAverage-1))))];

place=place+1; %#ok

end

elseif signal(2) == 1 && sum(signal) == 1

for place = 1:(length(cellList.meshData{frame}{cellNum}.relint2)-(numPixelsMovingAverage-1));

cellList.meshData{frame}{cellNum}.meshavg=[cellList.meshData{frame}{cellNum}.meshavg mean(cellList.meshData{frame}{cellNum}.relint2(place:(place+(numPixelsMovingAverage-1))))];

place=place+1; %#ok

end

elseif sum(signal) == 2

for place = 1:(length(cellList.meshData{frame}{cellNum}.relint2)-(numPixelsMovingAverage-1));

cellList.meshData{frame}{cellNum}.meshavg=[cellList.meshData{frame}{cellNum}.meshavg mean(cellList.meshData{frame}{cellNum}.relint2(place:(place+(numPixelsMovingAverage-1))))];

place=place+1; %#ok

end

end

%%WITH THE BRIGHTEST SEGMENT CALCULATED ABOVE WE CAN ORIENT THE

%%CELL SO THAT THE BRIGHTEST SEGMENT IS ON THE RIGHTS (i.e. WITH FtsZ BEING POLAR ON RIGHT(NEW POLE)

%%AND LARGER STALK CELL BIAS LETTING THE FtsZ RING BE ON THE RIGHT

%%AS WELL)

[~,maxavg]=max(cellList.meshData{frame}{cellNum}.meshavg);

if maxavg<=length(cellList.meshData{frame}{cellNum}.meshavg)/2+1; %#ok

if signal(1) ==1 && sum(signal) == 1

cellList.meshData{frame}{cellNum}.relint1=flipud(cellList.meshData{frame}{cellNum}.relint1);

elseif signal(2) == 1 && sum(signal) == 1

cellList.meshData{frame}{cellNum}.relint2=flipud(cellList.meshData{frame}{cellNum}.relint2);

elseif sum(signal) == 2

cellList.meshData{frame}{cellNum}.relint1=flipud(cellList.meshData{frame}{cellNum}.relint1);

cellList.meshData{frame}{cellNum}.relint2=flipud(cellList.meshData{frame}{cellNum}.relint2);

end

end

k = floor(cellList.meshData{frame}{cellNum}.length/2);

temp = cellList.meshData{frame}{cellNum}.lengthvector-cellList.meshData{frame}{cellNum}.length/2;

if signal(1) ==1 && sum(signal) == 1

interpint1 = interp1(temp(1:length(cellList.meshData{frame}{cellNum}.relint1)),cellList.meshData{frame}{cellNum}.relint1,-k:k,'linear','extrap');

relintarray1(maxsizel2a-k:maxsizel2a+k,passed)=interpint1;

elseif signal(2) == 1 && sum(signal) == 1

interpint2 = interp1(temp(1:length(cellList.meshData{frame}{cellNum}.relint2)),cellList.meshData{frame}{cellNum}.relint2,-k:k,'linear','extrap');

relintarray2(maxsizel2a-k:maxsizel2a+k,passed)=interpint2; %#ok<AGROW>

elseif sum(signal) == 2

interpint1 = interp1(temp(1:length(cellList.meshData{frame}{cellNum}.relint1)),cellList.meshData{frame}{cellNum}.relint1,-k:k,'linear','extrap');

relintarray1(maxsizel2a-k:maxsizel2a+k,passed)=interpint1;

interpint2 = interp1(temp(1:length(cellList.meshData{frame}{cellNum}.relint2)),cellList.meshData{frame}{cellNum}.relint2,-k:k,'linear','extrap');

relintarray2(maxsizel2a-k:maxsizel2a+k,passed)=interpint2; %#ok<AGROW>

end

cellLength=[cellLength cellList.meshData{frame}{cellNum}.length]; %#ok<AGROW>

end

end

% % cells length array is concatonated with the fluorescence matrix. This matrix is then sorted by length in ascending order

numlist=[1:1:maxCellNum]; %#ok

lvint0=cat(2,numlist',cellLength');

if signal(1) ==1 && sum(signal) == 1

lvint1=cat(2,lvint0,relintarray1');

lnumsort1=sortrows(lvint1,[2]); %#ok

elseif signal(2) == 1 && sum(signal) == 1

lvint2=cat(2,lvint0,relintarray2');

lnumsort2=sortrows(lvint2,[2]); %#ok

elseif sum(signal) == 2

lvint1=cat(2,lvint0,relintarray1');

lvint2=cat(2,lvint0,relintarray2');

lnumsort1=sortrows(lvint1,[2]); %#ok

lnumsort2=sortrows(lvint2,[2]); %#ok

end

if signal(1) ==1 && sum(signal) == 1

%relative intensities are plotted accoring to a colormap

x=[-conversionFactor*maxCellLength./2 conversionFactor*maxCellLength./2];

x = repmat(x(1):x(2)*2/(size(lnumsort1,2)-3):x(2),size(lnumsort1,1),1);

y = repmat((1:size(lnumsort1,1)),size(lnumsort1,2)-2,1)';

dataToPlot = lnumsort1(1:end,3:end);

dataToPlot(dataToPlot==0) = NaN;

pcolor(x,y,flipud(dataToPlot)); colormap jet; colorbar;caxis([0 1]);shading flat;

xlabel('Distance From Midcell (\mum)','FontSize',18)

ylabel('Number of Cells','FontSize',18)

elseif signal(2) == 1 && sum(signal) == 1

%relative intensities are plotted accoring to a colormap

x=[-conversionFactor*maxCellLength./2 conversionFactor*maxCellLength./2];

x = repmat(x(1):x(2)*2/(size(lnumsort2,2)-3):x(2),size(lnumsort2,1),1);

y = repmat((1:size(lnumsort2,1)),size(lnumsort2,2)-2,1)';

dataToPlot = lnumsort2(1:end,3:end);

dataToPlot(dataToPlot==0) = NaN;

pcolor(x,y,flipud(dataToPlot)); colormap jet; colorbar;caxis([0 1]);shading flat;

xlabel('Distance From Midcell (\mum)','FontSize',18)

ylabel('Number of Cells','FontSize',18)

elseif sum(signal) == 2

%relative intensities are plotted accoring to a colormap

x=[-conversionFactor*maxCellLength./2 conversionFactor*maxCellLength./2];

x = repmat(x(1):x(2)*2/(size(lnumsort1,2)-3):x(2),size(lnumsort1,1),1);

y = repmat((1:size(lnumsort1,1)),size(lnumsort1,2)-2,1)';

dataToPlot = lnumsort1(1:end,3:end);

dataToPlot(dataToPlot==0) = NaN;

pcolor(x,y,flipud(dataToPlot)); colormap jet; colorbar;caxis([0 1]);shading flat;

xlabel('Distance From Midcell (\mum)','FontSize',18)

ylabel('Number of Cells','FontSize',18)

%relative intensities are plotted accoring to a colormap

figure;

x=[-conversionFactor*maxCellLength./2 conversionFactor*maxCellLength./2];

x = repmat(x(1):x(2)*2/(size(lnumsort2,2)-3):x(2),size(lnumsort2,1),1);

y = repmat((1:size(lnumsort2,1)),size(lnumsort2,2)-2,1)';

dataToPlot = lnumsort2(1:end,3:end);

dataToPlot(dataToPlot==0) = NaN;

pcolor(x,y,flipud(dataToPlot)); colormap jet; colorbar;caxis([0 1]);shading flat;

xlabel('Distance From Midcell (\mum)','FontSize',18)

ylabel('Number of Cells','FontSize',18)

end

case 'normByPopulation'

replacement=false;

%%finds the maximum number of stepareas inside of a cell from the cellList

maxsizelarray=[];

intensityVector1Min = [];

intensityVector1Max = [];

intensityVector2Min = [];

intensityVector2Max = [];

n=0;

for frame = frameList

for cellNum = 1:length(cellList.meshData{frame})

if isempty(cellList.meshData{frame}{cellNum}) || ~isfield(cellList.meshData{frame}{cellNum},'mesh')||length(cellList.meshData{frame}{cellNum}.mesh)<4 ...

||~isfield(cellList.meshData{frame}{cellNum},'signal1') ...

|| isempty(cellList.meshData{frame}{cellNum}.signal1) || cellList.meshData{frame}{cellNum}.length>maxCellLength

continue

end

n=n+1;

end

end

if n<=maxCellNum

maxCellNum=n;

end

rand=randsample(n,maxCellNum,replacement);

n=0;

for frame = frameList

for cellNum = 1:length(cellList.meshData{frame})

if isempty(cellList.meshData{frame}{cellNum}) ...

|| length(cellList.meshData{frame}{cellNum}.mesh)<4 ...

|| ~isfield(cellList.meshData{frame}{cellNum},'signal1') ...

|| isempty(cellList.meshData{frame}{cellNum}.signal1) ...

|| cellList.meshData{frame}{cellNum}.length>maxCellLength

continue

end

n = n+1;

b=rand==n;

if sum(b)~=1

continue

end

maxsizelarray=[maxsizelarray length(cellList.meshData{frame}{cellNum}.lengthvector)];%#ok<AGROW>

try

intensityVector1Min=[intensityVector1Min min(cellList.meshData{frame}{cellNum}.signal1)];%#ok<AGROW>

intensityVector1Max=[intensityVector1Max max(cellList.meshData{frame}{cellNum}.signal1)];%#ok<AGROW>

catch

end

try

intensityVector2Min=[intensityVector2Min min(cellList.meshData{frame}{cellNum}.signal2)];%#ok<AGROW>

intensityVector2Max=[intensityVector2Max max(cellList.meshData{frame}{cellNum}.signal2)];%#ok<AGROW>

catch

end

end

end

%using the maxima from above, a matrix consiting of zeros is created to be

%filled in by mesh intensities

relintarray1=zeros(max(maxsizelarray),maxCellNum);

maxsizel=max(maxsizelarray);

try

% % % smallValueIntensityVector1Min = quantile(intensityVector1Min(intensityVector1Min~=0),0.3);

smallValueIntensityVector1Min = 0;

% % % largeValueIntensityVector1Max = max(intensityVector1Max);

largeValueIntensityVector1Max = quantile(intensityVector1Max(intensityVector1Max~=0),0.9);

catch

end

try

smallValueIntensityVector2Min = quantile(intensityVector2Min(intensityVector2Min~=0),0.3);

largeValueIntensityVector2Max = quantile(intensityVector2Max(intensityVector2Max~=0),0.9);

catch

end

% if maxCellLength > maxsizel;

% maxsizel=maxCellLength;

% end

maxsizel2 = ceil(maxsizel); if mod(maxsizel2,2)==0, maxsizel2=maxsizel2+1; end

maxsizel2a = maxsizel2/2+0.5;

n=0;

passed=0;

cellLength=[];

%zeroarray is replaced with relative segment intensity data from the cell

for frame = frameList

for cellNum = 1:length(cellList.meshData{frame})

place=1;%#ok

if isempty(cellList.meshData{frame}{cellNum}) || length(cellList.meshData{frame}{cellNum}.mesh)<4 ...

||~isfield(cellList.meshData{frame}{cellNum},'signal1') ...

|| isempty(cellList.meshData{frame}{cellNum}.signal1) || cellList.meshData{frame}{cellNum}.length>maxCellLength

continue

end

n = n+1;

b=rand==n;

if sum(b)~=1

continue

end

passed=passed+1;

if signal(1) == 1 && sum(signal) == 1

%%calculates the fluorescent intensities in each segment normalized

%%by the area of that segment

cellList.meshData{frame}{cellNum}.relsignal1 = (cellList.meshData{frame}{cellNum}.signal1 - smallValueIntensityVector1Min)./(largeValueIntensityVector1Max - smallValueIntensityVector1Min);

cellList.meshData{frame}{cellNum}.relint1 = cellList.meshData{frame}{cellNum}.relsignal1;

%%segments are then normalized to the brightest segment so that

%%this sigment is represented as 1.

% % % cellList.meshData{frame}{cellNum}.relint1 = (cellList.meshData{frame}{cellNum}.relsignal1./max(cellList.meshData{frame}{cellNum}.relsignal1));

elseif signal(2) == 1 && sum(signal) == 1

%%calculates the fluorescent intensities in each segment normalized

%%by the area of that segment

cellList.meshData{frame}{cellNum}.relsignal2 = (cellList.meshData{frame}{cellNum}.signal2 - smallValueIntensityVector2Min)./(largeValueIntensityVector2Max - smallValueIntensityVector2Min);

cellList.meshData{frame}{cellNum}.relint2 = cellList.meshData{frame}{cellNum}.relsignal2;

%%segments are then normalized to the brightest segment so that

%%this sigment is represented as 1.

% % % cellList.meshData{frame}{cellNum}.relint2 = (cellList.meshData{frame}{cellNum}.relsignal2./max(cellList.meshData{frame}{cellNum}.relsignal2));

elseif sum(signal) == 2

%%calculates the fluorescent intensities in each segment normalized

%%by the area of that segment

cellList.meshData{frame}{cellNum}.relsignal1 = (cellList.meshData{frame}{cellNum}.signal1 - smallValueIntensityVector1Min)./(largeValueIntensityVector1Max - smallValueIntensityVector1Min);

cellList.meshData{frame}{cellNum}.relint1 = cellList.meshData{frame}{cellNum}.relsignal1;

%%segments are then normalized to the brightest segment so that

%%this sigment is represented as 1.

% % % cellList.meshData{frame}{cellNum}.relint1 = (cellList.meshData{frame}{cellNum}.relsignal1./max(cellList.meshData{frame}{cellNum}.relsignal1));

%%calculates the fluorescent intensities in each segment normalized

%%by the area of that segment

cellList.meshData{frame}{cellNum}.relsignal2 = (cellList.meshData{frame}{cellNum}.signal2 - smallValueIntensityVector2Min)./(largeValueIntensityVector2Max - smallValueIntensityVector2Min);

cellList.meshData{frame}{cellNum}.relint2 = cellList.meshData{frame}{cellNum}.relsignal2;

%%segments are then normalized to the brightest segment so that

%%this sigment is represented as 1.

% % % cellList.meshData{frame}{cellNum}.relint2 = (cellList.meshData{frame}{cellNum}.relsignal2./max(cellList.meshData{frame}{cellNum}.relsignal2));

else

disp('provide information in signal variable')

return;

end

%%% A MOVING AVERAGE IS CALCULATED FOR EACH OF THE SEGMENTS TO FIND THE SINGLE BRIGHTEST SEGMENT AREA

cellList.meshData{frame}{cellNum}.meshavg=[];

if signal(1) == 1 && sum(signal) == 1

for place = 1:(length(cellList.meshData{frame}{cellNum}.relint1)-(numPixelsMovingAverage-1));

cellList.meshData{frame}{cellNum}.meshavg=[cellList.meshData{frame}{cellNum}.meshavg mean(cellList.meshData{frame}{cellNum}.relint1(place:(place+(numPixelsMovingAverage-1))))];

place=place+1; %#ok

end

elseif signal(2) == 1 && sum(signal) == 1

for place = 1:(length(cellList.meshData{frame}{cellNum}.relint2)-(numPixelsMovingAverage-1));

cellList.meshData{frame}{cellNum}.meshavg=[cellList.meshData{frame}{cellNum}.meshavg mean(cellList.meshData{frame}{cellNum}.relint2(place:(place+(numPixelsMovingAverage-1))))];

place=place+1; %#ok

end

elseif sum(signal1) == 2

for place = 1:(length(cellList.meshData{frame}{cellNum}.relint2)-(numPixelsMovingAverage-1));

cellList.meshData{frame}{cellNum}.meshavg=[cellList.meshData{frame}{cellNum}.meshavg mean(cellList.meshData{frame}{cellNum}.relint2(place:(place+(numPixelsMovingAverage-1))))];

place=place+1; %#ok

end

end

k = floor(cellList.meshData{frame}{cellNum}.length/2);

temp = cellList.meshData{frame}{cellNum}.lengthvector-cellList.meshData{frame}{cellNum}.length/2;

if signal(1) ==1 && sum(signal) == 1

interpint1 = interp1(temp(1:length(cellList.meshData{frame}{cellNum}.relint1)),cellList.meshData{frame}{cellNum}.relint1,-k:k,'linear','extrap');

relintarray1(maxsizel2a-k:maxsizel2a+k,passed)=interpint1;

elseif signal(2) == 1 && sum(signal) == 1

interpint2 = interp1(temp(1:length(cellList.meshData{frame}{cellNum}.relint2)),cellList.meshData{frame}{cellNum}.relint2,-k:k,'linear','extrap');

relintarray2(maxsizel2a-k:maxsizel2a+k,passed)=interpint2; %#ok<AGROW>

elseif sum(signal) == 2

interpint1 = interp1(temp(1:length(cellList.meshData{frame}{cellNum}.relint1)),cellList.meshData{frame}{cellNum}.relint1,-k:k,'linear','extrap');

relintarray1(maxsizel2a-k:maxsizel2a+k,passed)=interpint1;

interpint2 = interp1(temp(1:length(cellList.meshData{frame}{cellNum}.relint2)),cellList.meshData{frame}{cellNum}.relint2,-k:k,'linear','extrap');

relintarray2(maxsizel2a-k:maxsizel2a+k,passed)=interpint2; %#ok<AGROW>

end

cellLength=[cellLength cellList.meshData{frame}{cellNum}.length]; %#ok<AGROW>

end

end

% % cells length array is concatonated with the fluorescence matrix. This matrix is then sorted by length in ascending order

numlist=[1:1:maxCellNum]; %#ok

lvint0=cat(2,numlist',cellLength');

if signal(1) ==1 && sum(signal) == 1

lvint1=cat(2,lvint0,relintarray1');

lnumsort1=sortrows(lvint1,[2]); %#ok

elseif signal(2) == 1 && sum(signal) == 1

lvint2=cat(2,lvint0,relintarray2');

lnumsort2=sortrows(lvint2,[2]); %#ok

elseif sum(signal) == 2

lvint1=cat(2,lvint0,relintarray1');

lvint2=cat(2,lvint0,relintarray2');

lnumsort1=sortrows(lvint1,[2]); %#ok

lnumsort2=sortrows(lvint2,[2]); %#ok

end

if signal(1) ==1 && sum(signal) == 1

%relative intensities are plotted accoring to a colormap

x=[-conversionFactor*maxCellLength./2 conversionFactor*maxCellLength./2];

x = repmat(x(1):x(2)*2/(size(lnumsort1,2)-3):x(2),size(lnumsort1,1),1);

y = repmat((1:size(lnumsort1,1)),size(lnumsort1,2)-2,1)';

dataToPlot = lnumsort1(1:end,3:end);

dataToPlot(dataToPlot==0) = NaN;

pcolor(x,y,flipud(dataToPlot)); colormap jet; colorbar;caxis([0 1]);shading flat;

xlabel('Distance From Midcell (\mum)','FontSize',18)

ylabel('Number of Cells','FontSize',18)

elseif signal(2) == 1 && sum(signal) == 1

%relative intensities are plotted accoring to a colormap

x=[-conversionFactor*maxCellLength./2 conversionFactor*maxCellLength./2];

x = repmat(x(1):x(2)*2/(size(lnumsort2,2)-3):x(2),size(lnumsort2,1),1);

y = repmat((1:size(lnumsort2,1)),size(lnumsort2,2)-2,1)';

dataToPlot = lnumsort2(1:end,3:end);

dataToPlot(dataToPlot==0) = NaN;

pcolor(x,y,flipud(dataToPlot)); colormap jet; colorbar;caxis([0 1]);shading flat;

xlabel('Distance From Midcell (\mum)','FontSize',18)

ylabel('Number of Cells','FontSize',18)

elseif sum(signal) == 2

%relative intensities are plotted accoring to a colormap

x=[-conversionFactor*maxCellLength./2 conversionFactor*maxCellLength./2];

x = repmat(x(1):x(2)*2/(size(lnumsort1,2)-3):x(2),size(lnumsort1,1),1);

y = repmat((1:size(lnumsort1,1)),size(lnumsort1,2)-2,1)';

dataToPlot = lnumsort1(1:end,3:end);

dataToPlot(dataToPlot==0) = NaN;

pcolor(x,y,flipud(dataToPlot)); colormap jet; colorbar;caxis([0 1]);shading flat;

xlabel('Distance From Midcell (\mum)','FontSize',18)

ylabel('Number of Cells','FontSize',18)

%relative intensities are plotted accoring to a colormap

figure;

x=[-conversionFactor*maxCellLength./2 conversionFactor*maxCellLength./2];

x = repmat(x(1):x(2)*2/(size(lnumsort2,2)-3):x(2),size(lnumsort2,1),1);

y = repmat((1:size(lnumsort2,1)),size(lnumsort2,2)-2,1)';

dataToPlot = lnumsort2(1:end,3:end);

dataToPlot(dataToPlot==0) = NaN;

pcolor(x,y,flipud(dataToPlot)); colormap jet; colorbar;caxis([0 1]);shading flat;

xlabel('Distance From Midcell (\mum)','FontSize',18)

ylabel('Number of Cells','FontSize',18)

end

case 'randomN'

try

replacement=false;

%%finds the maximum number of stepareas inside of a cell from the cellList

maxsizelarray=[];

n=0;

for frame = frameList

for cellNum = 1:length(cellList.meshData{frame})

if isempty(cellList.meshData{frame}{cellNum}) || ~isfield(cellList.meshData{frame}{cellNum},'mesh')||length(cellList.meshData{frame}{cellNum}.mesh)<4 ...

||~isfield(cellList.meshData{frame}{cellNum},signalInfo) ...

|| eval('isempty(cellList.meshData{frame}{cellNum}.(signalInfo))') || cellList.meshData{frame}{cellNum}.length>maxCellLength

continue

end

n=n+1;

end

end

if n<=maxCellNum

maxCellNum=n;

end

rand=randsample(n,maxCellNum,replacement);

n=0;

for frame = frameList

for cellNum = 1:length(cellList.meshData{frame})

if isempty(cellList.meshData{frame}{cellNum}) ...

|| length(cellList.meshData{frame}{cellNum}.mesh)<4 ...

|| ~isfield(cellList.meshData{frame}{cellNum},signalInfo) ...

|| eval('isempty(cellList.meshData{frame}{cellNum}.(signalInfo))') ...

|| cellList.meshData{frame}{cellNum}.length>maxCellLength

continue

end

n = n+1;

b=rand==n;

if sum(b)~=1

continue

end

maxsizelarray=[maxsizelarray length(cellList.meshData{frame}{cellNum}.lengthvector)];%#ok<AGROW>

end

end

if isempty(maxsizelarray)

warndlg(['No field ' signalInfo ' recorded for this cell: Use Reuse meshes toggle button to compute ' signalInfo]);

return;

end

%using the maxima from above, a matrix consiting of zeros is created to be

%filled in by mesh intensities

relintarray1=zeros(max(maxsizelarray),maxCellNum);

maxsizel=max(maxsizelarray);

if maxCellLength > maxsizel;

maxCellLength = maxsizel;

end

maxsizel2 = ceil(maxsizel); if mod(maxsizel2,2)==0, maxsizel2=maxsizel2+1; end

maxsizel2a = maxsizel2/2+0.5;

n=0;

passed=0;

cellLength=[];

%zeroarray is replaced with relative segment intensity data from the cell

for frame = frameList

for cellNum = 1:length(cellList.meshData{frame})

place=1;%#ok

if isempty(cellList.meshData{frame}{cellNum}) || length(cellList.meshData{frame}{cellNum}.mesh)<4 ...

||~isfield(cellList.meshData{frame}{cellNum},signalInfo) ...

|| eval('isempty(cellList.meshData{frame}{cellNum}.(signalInfo))') || cellList.meshData{frame}{cellNum}.length>maxCellLength

continue

end

n = n+1;

b=rand==n;

if sum(b)~=1

continue

end

passed=passed+1;

if signal(1) == 1 && sum(signal) == 1

%%calculates the fluorescent intensities in each segment normalized

%%by the area of that segment

if length(cellList.meshData{frame}{cellNum}.signal1) > length(cellList.meshData{frame}{cellNum}.steparea)

cellList.meshData{frame}{cellNum}.relsignal1 = (cellList.meshData{frame}{cellNum}.signal1(1:length(cellList.meshData{frame}{cellNum}.steparea))./cellList.meshData{frame}{cellNum}.steparea);

else

cellList.meshData{frame}{cellNum}.relsignal1 = (cellList.meshData{frame}{cellNum}.signal1./cellList.meshData{frame}{cellNum}.steparea(1:length(cellList.meshData{frame}{cellNum}.signal1)));

end

%%segments are then normalized to the brightest segment so that

%%this sigment is represented as 1.

cellList.meshData{frame}{cellNum}.relint1 = (cellList.meshData{frame}{cellNum}.relsignal1./max(cellList.meshData{frame}{cellNum}.relsignal1));

elseif signal(2) == 1 && sum(signal) == 1

%%calculates the fluorescent intensities in each segment normalized

%%by the area of that segment

if length(cellList.meshData{frame}{cellNum}.signal2) > length(cellList.meshData{frame}{cellNum}.steparea)

cellList.meshData{frame}{cellNum}.relsignal2 = (cellList.meshData{frame}{cellNum}.signal2(1:length(cellList.meshData{frame}{cellNum}.steparea))./cellList.meshData{frame}{cellNum}.steparea);

else

cellList.meshData{frame}{cellNum}.relsignal2 = (cellList.meshData{frame}{cellNum}.signal2./cellList.meshData{frame}{cellNum}.steparea(1:length(cellList.meshData{frame}{cellNum}.signal2)));

end

%%segments are then normalized to the brightest segment so that

%%this sigment is represented as 1.

cellList.meshData{frame}{cellNum}.relint2 = (cellList.meshData{frame}{cellNum}.relsignal2./max(cellList.meshData{frame}{cellNum}.relsignal2));

elseif sum(signal) == 2

%%calculates the fluorescent intensities in each segment normalized

%%by the area of that segment

if length(cellList.meshData{frame}{cellNum}.signal1) > length(cellList.meshData{frame}{cellNum}.steparea)

cellList.meshData{frame}{cellNum}.relsignal1 = (cellList.meshData{frame}{cellNum}.signal1(1:length(cellList.meshData{frame}{cellNum}.steparea))./cellList.meshData{frame}{cellNum}.steparea);

else

cellList.meshData{frame}{cellNum}.relsignal1 = (cellList.meshData{frame}{cellNum}.signal1./cellList.meshData{frame}{cellNum}.steparea(1:length(cellList.meshData{frame}{cellNum}.signal1)));

end

%%segments are then normalized to the brightest segment so that

%%this sigment is represented as 1.

cellList.meshData{frame}{cellNum}.relint1 = (cellList.meshData{frame}{cellNum}.relsignal1./max(cellList.meshData{frame}{cellNum}.relsignal1));

%%calculates the fluorescent intensities in each segment normalized

%%by the area of that segment

if length(cellList.meshData{frame}{cellNum}.signal2) > length(cellList.meshData{frame}{cellNum}.steparea)

cellList.meshData{frame}{cellNum}.relsignal2 = (cellList.meshData{frame}{cellNum}.signal2(1:length(cellList.meshData{frame}{cellNum}.steparea))./cellList.meshData{frame}{cellNum}.steparea);

else

cellList.meshData{frame}{cellNum}.relsignal2 = (cellList.meshData{frame}{cellNum}.signal2./cellList.meshData{frame}{cellNum}.steparea(1:length(cellList.meshData{frame}{cellNum}.signal2)));

end

%%segments are then normalized to the brightest segment so that

%%this sigment is represented as 1.

cellList.meshData{frame}{cellNum}.relint2 = (cellList.meshData{frame}{cellNum}.relsignal2./max(cellList.meshData{frame}{cellNum}.relsignal2));

else

disp('provide information in signal variable')

return;

end

%%% A MOVING AVERAGE IS CALCULATED FOR EACH OF THE SEGMENTS TO FIND THE SINGLE BRIGHTEST SEGMENT AREA

cellList.meshData{frame}{cellNum}.meshavg=[];

if signal(1) == 1 && sum(signal) == 1

for place = 1:(length(cellList.meshData{frame}{cellNum}.relint1)-(numPixelsMovingAverage-1));

cellList.meshData{frame}{cellNum}.meshavg=[cellList.meshData{frame}{cellNum}.meshavg mean(cellList.meshData{frame}{cellNum}.relint1(place:(place+(numPixelsMovingAverage-1))))];

place=place+1; %#ok

end

elseif signal(2) == 1 && sum(signal) == 1

for place = 1:(length(cellList.meshData{frame}{cellNum}.relint2)-(numPixelsMovingAverage-1));

cellList.meshData{frame}{cellNum}.meshavg=[cellList.meshData{frame}{cellNum}.meshavg mean(cellList.meshData{frame}{cellNum}.relint2(place:(place+(numPixelsMovingAverage-1))))];

place=place+1; %#ok

end

end

%%WITH THE BRIGHTEST SEGMENT CALCULATED ABOVE WE CAN ORIENT THE

%%CELL SO THAT THE BRIGHTEST SEGMENT IS ON THE RIGHTS (i.e. WITH FtsZ BEING POLAR ON RIGHT(NEW POLE)

%%AND LARGER STALK CELL BIAS LETTING THE FtsZ RING BE ON THE RIGHT

%%AS WELL)

% % % [~,maxavg]=max(cellList.meshData{frame}{cellNum}.meshavg);

% % % if maxavg<=length(cellList.meshData{frame}{cellNum}.meshavg)/2+1; %#ok

% % % if signal(1) ==1 && sum(signal) == 1

% % % cellList.meshData{frame}{cellNum}.relint1=flipud(cellList.meshData{frame}{cellNum}.relint1);

% % % elseif signal(2) == 1 && sum(signal) == 1

% % % cellList.meshData{frame}{cellNum}.relint2=flipud(cellList.meshData{frame}{cellNum}.relint2);

% % % elseif sum(signal) == 2

% % % cellList.meshData{frame}{cellNum}.relint1=flipud(cellList.meshData{frame}{cellNum}.relint1);

% % % cellList.meshData{frame}{cellNum}.relint2=flipud(cellList.meshData{frame}{cellNum}.relint2);

% % % end

% % %

% % % end

k = floor(cellList.meshData{frame}{cellNum}.length/2);

temp = cellList.meshData{frame}{cellNum}.lengthvector-cellList.meshData{frame}{cellNum}.length/2;

if signal(1) ==1 && sum(signal) == 1

interpint1 = interp1(temp(1:length(cellList.meshData{frame}{cellNum}.relint1)),cellList.meshData{frame}{cellNum}.relint1,-k:k,'linear','extrap');

relintarray1(maxsizel2a-k:maxsizel2a+k,passed)=interpint1;

elseif signal(2) == 1 && sum(signal) == 1

interpint2 = interp1(temp(1:length(cellList.meshData{frame}{cellNum}.relint2)),cellList.meshData{frame}{cellNum}.relint2,-k:k,'linear','extrap');

relintarray2(maxsizel2a-k:maxsizel2a+k,passed)=interpint2; %#ok<AGROW>

elseif sum(signal) == 2

interpint1 = interp1(temp(1:length(cellList.meshData{frame}{cellNum}.relint1)),cellList.meshData{frame}{cellNum}.relint1,-k:k,'linear','extrap');

relintarray1(maxsizel2a-k:maxsizel2a+k,passed)=interpint1;

interpint2 = interp1(temp(1:length(cellList.meshData{frame}{cellNum}.relint2)),cellList.meshData{frame}{cellNum}.relint2,-k:k,'linear','extrap');

relintarray2(maxsizel2a-k:maxsizel2a+k,passed)=interpint2; %#ok<AGROW>

end

cellLength=[cellLength cellList.meshData{frame}{cellNum}.length]; %#ok<AGROW>

end

end

% % cells length array is concatonated with the fluorescence matrix. This matrix is then sorted by length in ascending order

numlist=[1:1:maxCellNum]; %#ok

lvint0=cat(2,numlist',cellLength');

if signal(1) ==1 && sum(signal) == 1

lvint1=cat(2,lvint0,relintarray1');

lnumsort1=sortrows(lvint1,[2]); %#ok

elseif signal(2) == 1 && sum(signal) == 1

lvint2=cat(2,lvint0,relintarray2');

lnumsort2=sortrows(lvint2,[2]); %#ok

elseif sum(signal) == 2

lvint1=cat(2,lvint0,relintarray1');

lvint2=cat(2,lvint0,relintarray2');

lnumsort1=sortrows(lvint1,[2]); %#ok

lnumsort2=sortrows(lvint2,[2]); %#ok

end

if signal(1) ==1 && sum(signal) == 1

%relative intensities are plotted accoring to a colormap

x=[-conversionFactor*maxCellLength./2 conversionFactor*maxCellLength./2];

x = repmat(x(1):x(2)*2/(size(lnumsort1,2)-3):x(2),size(lnumsort1,1),1);

y = repmat((1:size(lnumsort1,1)),size(lnumsort1,2)-2,1)';

dataToPlot = lnumsort1(1:end,3:end);

dataToPlot(dataToPlot==0) = NaN;

pcolor(x,y,flipud(dataToPlot)); colormap jet; colorbar;caxis([0 1]);shading flat;

xlabel('Distance From Midcell (\mum)','FontSize',18)

ylabel('Number of Cells','FontSize',18)

elseif signal(2) == 1 && sum(signal) == 1

%relative intensities are plotted accoring to a colormap

x=[-conversionFactor*maxCellLength./2 conversionFactor*maxCellLength./2];

x = repmat(x(1):x(2)*2/(size(lnumsort2,2)-3):x(2),size(lnumsort2,1),1);

y = repmat((1:size(lnumsort2,1)),size(lnumsort2,2)-2,1)';

dataToPlot = lnumsort2(1:end,3:end);

dataToPlot(dataToPlot==0) = NaN;

pcolor(x,y,flipud(dataToPlot)); colormap jet; colorbar;caxis([0 1]);shading flat;

xlabel('Distance From Midcell (\mum)','FontSize',18)

ylabel('Number of Cells','FontSize',18)

elseif sum(signal) == 2

%relative intensities are plotted accoring to a colormap

x=[-conversionFactor*maxCellLength./2 conversionFactor*maxCellLength./2];

x = repmat(x(1):x(2)*2/(size(lnumsort1,2)-3):x(2),size(lnumsort1,1),1);

y = repmat((1:size(lnumsort1,1)),size(lnumsort1,2)-2,1)';

dataToPlot = lnumsort1(1:end,3:end);

dataToPlot(dataToPlot==0) = NaN;

pcolor(x,y,flipud(dataToPlot)); colormap jet; colorbar;caxis([0 1]);shading flat;

xlabel('Distance From Midcell (\mum)','FontSize',18)

ylabel('Number of Cells','FontSize',18)

%relative intensities are plotted accoring to a colormap

figure;

x=[-conversionFactor*maxCellLength./2 conversionFactor*maxCellLength./2];

x = repmat(x(1):x(2)*2/(size(lnumsort2,2)-3):x(2),size(lnumsort2,1),1);

y = repmat((1:size(lnumsort2,1)),size(lnumsort2,2)-2,1)';

dataToPlot = lnumsort2(1:end,3:end);

dataToPlot(dataToPlot==0) = NaN;

pcolor(x,y,flipud(dataToPlot)); colormap jet; colorbar;caxis([0 1]);shading flat;

xlabel('Distance From Midcell (\mum)','FontSize',18)

ylabel('Number of Cells','FontSize',18)

end

catch err

if strcmpi(err.identifier,'MATLAB:catenate:dimensionMismatch')

warndlg('Choose a smaller number for max cell number parameter');

return;

end

end

case 'constriction'

try

DC = [];

replacement=false; %#ok

%%finds the maximum number of stepareas inside of a cell from the cellList.meshData

sizel=[];

sizelarray=[];

for frame = frameList

for cellNum = 1:length(cellList.meshData{frame})

if ~isfield(cellList.meshData{frame}{cellNum},signalInfo) || eval('isempty(cellList.meshData{frame}{cellNum}.(signalInfo))') || cellList.meshData{frame}{cellNum}.length>maxCellLength

continue

end

sizelarray= [sizelarray length(cellList.meshData{frame}{cellNum}.lengthvector)];%#ok<AGROW>

end

end

if isempty(sizelarray)

warndlg(['No field ' signalInfo ' recorded for this cell: Use Reuse meshes toggle button to compute ' signalInfo]);

return;

end

maxsizel=max(sizelarray);

if maxCellLength > maxsizel;

maxCellLength = maxsizel;

end

%using the maxima from above, a (max X cell number)matrix consiting of zeros is created

% rand=randsample(length(sizelarray),maxCellNum,replacement);

maxCellLength2 = ceil(maxCellLength); if mod(maxCellLength2,2)==0, maxCellLength2=maxCellLength2+1; end

maxCellLength2a = maxCellLength2/2+0.5;

relintarray1=zeros(maxCellLength2,length(sizelarray));

lengthvectorarray=zeros(maxCellLength,length(sizelarray));

n=0;

cellLength=[];

cellArea=[];

%zeroarray is replaced with relative segment intensity data from the cell

for frame = frameList

for cellNum = 1:length(cellList.meshData{frame})

place=1; %#ok

if ~isfield(cellList.meshData{frame}{cellNum},signalInfo) || eval('isempty(cellList.meshData{frame}{cellNum}.(signalInfo))') || cellList.meshData{frame}{cellNum}.length>maxCellLength

continue

end

n = n+1;

if signal(1) == 1 && sum(signal) == 1

%%calculates the fluorescent intensities in each segment normalized

%%by the area of that segment

if length(cellList.meshData{frame}{cellNum}.signal1) > length(cellList.meshData{frame}{cellNum}.steparea)

cellList.meshData{frame}{cellNum}.relsignal1 = (cellList.meshData{frame}{cellNum}.signal1(1:length(cellList.meshData{frame}{cellNum}.steparea))./cellList.meshData{frame}{cellNum}.steparea);

else

cellList.meshData{frame}{cellNum}.relsignal1 = (cellList.meshData{frame}{cellNum}.signal1./cellList.meshData{frame}{cellNum}.steparea(1:length(cellList.meshData{frame}{cellNum}.signal1)));

end

%%segments are then normalized to the brightest segment so that

%%this sigment is represented as 1.

cellList.meshData{frame}{cellNum}.relint1 = (cellList.meshData{frame}{cellNum}.relsignal1./max(cellList.meshData{frame}{cellNum}.relsignal1));

elseif signal(2) == 1 && sum(signal) == 1

%%calculates the fluorescent intensities in each segment normalized

%%by the area of that segment

if length(cellList.meshData{frame}{cellNum}.signal2) > length(cellList.meshData{frame}{cellNum}.steparea)

cellList.meshData{frame}{cellNum}.relsignal2 = (cellList.meshData{frame}{cellNum}.signal2(1:length(cellList.meshData{frame}{cellNum}.steparea))./cellList.meshData{frame}{cellNum}.steparea);

else

cellList.meshData{frame}{cellNum}.relsignal2 = (cellList.meshData{frame}{cellNum}.signal2./cellList.meshData{frame}{cellNum}.steparea(1:length(cellList.meshData{frame}{cellNum}.signal2)));

end

%%segments are then normalized to the brightest segment so that

%%this sigment is represented as 1.

cellList.meshData{frame}{cellNum}.relint2 = (cellList.meshData{frame}{cellNum}.relsignal2./max(cellList.meshData{frame}{cellNum}.relsignal2));

elseif sum(signal) == 2

%%calculates the fluorescent intensities in each segment normalized

%%by the area of that segment

if length(cellList.meshData{frame}{cellNum}.signal1) > length(cellList.meshData{frame}{cellNum}.steparea)

cellList.meshData{frame}{cellNum}.relsignal1 = (cellList.meshData{frame}{cellNum}.signal1(1:length(cellList.meshData{frame}{cellNum}.steparea))./cellList.meshData{frame}{cellNum}.steparea);

else

cellList.meshData{frame}{cellNum}.relsignal1 = (cellList.meshData{frame}{cellNum}.signal1./cellList.meshData{frame}{cellNum}.steparea(1:length(cellList.meshData{frame}{cellNum}.signal1)));

end

%%segments are then normalized to the brightest segment so that

%%this sigment is represented as 1.

cellList.meshData{frame}{cellNum}.relint1 = (cellList.meshData{frame}{cellNum}.relsignal1./max(cellList.meshData{frame}{cellNum}.relsignal1));

%%calculates the fluorescent intensities in each segment normalized

%%by the area of that segment

if length(cellList.meshData{frame}{cellNum}.signal2) > length(cellList.meshData{frame}{cellNum}.steparea)

cellList.meshData{frame}{cellNum}.relsignal2 = (cellList.meshData{frame}{cellNum}.signal2(1:length(cellList.meshData{frame}{cellNum}.steparea))./cellList.meshData{frame}{cellNum}.steparea);

else

cellList.meshData{frame}{cellNum}.relsignal2 = (cellList.meshData{frame}{cellNum}.signal2./cellList.meshData{frame}{cellNum}.steparea(1:length(cellList.meshData{frame}{cellNum}.signal2)));

end

%%segments are then normalized to the brightest segment so that

%%this sigment is represented as 1.

cellList.meshData{frame}{cellNum}.relint2 = (cellList.meshData{frame}{cellNum}.relsignal2./max(cellList.meshData{frame}{cellNum}.relsignal2));

else

disp('provide information in signal variable')

return;

end

cellList.meshData{frame}{cellNum}.meshavg=[];

if signal(1) == 1 && sum(signal) == 1

for place = 1:(length(cellList.meshData{frame}{cellNum}.relint1)-(numPixelsMovingAverage-1));

cellList.meshData{frame}{cellNum}.meshavg=[cellList.meshData{frame}{cellNum}.meshavg mean(cellList.meshData{frame}{cellNum}.relint1(place:(place+(numPixelsMovingAverage-1))))];

place=place+1; %#ok

end

elseif signal(2) == 1 && sum(signal) == 1

for place = 1:(length(cellList.meshData{frame}{cellNum}.relint2)-(numPixelsMovingAverage-1));

cellList.meshData{frame}{cellNum}.meshavg=[cellList.meshData{frame}{cellNum}.meshavg mean(cellList.meshData{frame}{cellNum}.relint2(place:(place+(numPixelsMovingAverage-1))))];

place=place+1; %#ok

end

end

% [qwert,maxavg]=max(cellList.meshData{f}{c}.meshavg);

% if maxavg<=length(cellList.meshData{f}{c}.meshavg)/2+1;

% % cellList.meshData{f}{c}.relint2=flipud(cellList.meshData{f}{c}.relint2);

% cellList.meshData{f}{c}.relint1=flipud(cellList.meshData{f}{c}.relint1);

% end

lngvector = cellList.meshData{frame}{cellNum}.lengthvector;

lng = cellList.meshData{frame}{cellNum}.length;

k = floor(lng/2);

if signal(1) == 1 && sum(signal) == 1

relint1=cellList.meshData{frame}{cellNum}.relint1;

interpint1 = interp1(lngvector-lng/2,relint1,-k:k,'linear','extrap');

relintarray1(maxCellLength2a-k:maxCellLength2a+k,n)=interpint1;

ind1 = length(relint1);

elseif signal(2) == 1 && sum(signal) == 1

relint2=cellList.meshData{frame}{cellNum}.relint2;

interpint2 = interp1(lngvector-lng/2,relint2,-k:k,'linear','extrap');

relintarray2(maxCellLength2a-k:maxCellLength2a+k,n)=interpint2; %#ok<AGROW>

ind1 = length(relint2);

elseif sum(signal) == 2

relint1=cellList.meshData{frame}{cellNum}.relint1;

interpint1 = interp1(lngvector-lng/2,relint1,-k:k,'linear','extrap');

relintarray1(maxCellLength2a-k:maxCellLength2a+k,n)=interpint1;

relint2=cellList.meshData{frame}{cellNum}.relint2;

interpint2 = interp1(lngvector-lng/2,relint2,-k:k,'linear','extrap');

relintarray2(maxCellLength2a-k:maxCellLength2a+k,n)=interpint2;%#ok<AGROW>

end

ind2 = round(maxCellLength/2-ind1/2);

lengthvectorarray(ind2+1:ind2+ind1,n)=cellList.meshData{frame}{cellNum}.lengthvector;

cellL=cellList.meshData{frame}{cellNum}.length;

cellLength=[cellLength cellL];%#ok<AGROW>

cellArea=[cellArea cellList.meshData{frame}{cellNum}.area];%#ok<AGROW>

prf = cellList.meshData{frame}{cellNum}.signal0;

if isempty(prf),break; end

for i=1:2

prf = 0.5*prf + 0.25*(prf([1 1:end-1])+prf([2:end end]));

end

minima = [false reshape((prf(2:end-1)<prf(1:end-2))&(prf(2:end-1)<prf(3:end)),1,[]) false];

if isempty(minima) || sum(prf)==0

minsize=0;

ctpos = []; %#ok

else

im = find(minima);

minsize = 0; %#ok

ctpos = 0; %#ok

dh = [];

dhi = [];

hgt = [];

for k=1:length(im)

i=im(k);

half1 = prf(1:i-1);

half2 = prf(i+1:end);

dh1 = max(half1)-prf(i);

dh2 = max(half2)-prf(i);

dh(k) = min(dh1,dh2); %#ok

dhi(k) = mean([dh1 dh2]); %#ok

hgt(k) = prf(i)+dhi(k); %#ok

end

[~,i] = max(dh);

minsizeabs = dhi(i);

minsize = minsizeabs/hgt(i);

ctpos = im(i); %#ok

if isempty(minsize), minsize=0; end

end

DC = [DC minsize]; %#ok<AGROW>

end

end

% % % cells are sorted by length in ascending order

numlist=[1:1:n]; %#ok

reverseNumlist=[n:-1:1]; %#ok

plotsizel=2+maxCellLength;

if signal(1) ==1 && sum(signal) == 1

lvint1=cat(2,cellLength',DC');

lvint1=cat(2,lvint1,relintarray1');

lnumsort1=sortrows(lvint1,[1]); %#ok

elseif signal(2) == 1 && sum(signal) == 1

lvint2=cat(2,cellLength',DC');

lvint2=cat(2,lvint2,relintarray2');

lnumsort2=sortrows(lvint2,[1]); %#ok

elseif sum(signal) == 2

lvint1=cat(2,cellLength',DC');

lvint1=cat(2,lvint1,relintarray1');

lnumsort1=sortrows(lvint1,[1]); %#ok

lvint2=cat(2,cellLength',DC');

lvint2=cat(2,lvint2,relintarray2');

lnumsort2=sortrows(lvint2,[1]); %#ok

end

% stepwidth=cat(2,cellLength',numlist');

% stepwidth=cat(2,stepwidth,relwidtharray');

% lwidthsort=sortrows(stepwidth,[1]);

% plotsizel=2+maxCellLength;

% % % lengthvectorarray=cat(2,cellLength',lengthvectorarray');

% % % lengthvectorarray=sortrows(lengthvectorarray,[1]);

% % % length_int=cat(2,lengthvectorarray,lnumsort1);

% % % sizelengthint=length(length_int);

% % % %relative intensities are plotted accoring to a colormap

% % % rand=randsample(length(lnumsort1),maxCellNum,replacement);

if signal(1) ==1 && sum(signal) == 1

x=[-conversionFactor*maxCellLength./2 conversionFactor*maxCellLength./2];

x = repmat(x(1):x(2)*2/(size(lnumsort1,2)-3):x(2),size(lnumsort1,1),1);

y = repmat((1:size(lnumsort1,1)),size(lnumsort1,2)-2,1)';

dataToPlot = lnumsort1(1:end,3:end);

dataToPlot(dataToPlot==0) = NaN;

pcolor(x,y,flipud(dataToPlot)); colormap jet; colorbar;caxis([0 1]);shading flat;

xlabel('Distance From Midcell (\mum)','FontSize',18)

ylabel('Number of Cells','FontSize',18)

figure,scatter(lnumsort1(1:end,2),reverseNumlist)

xlabel('Constriction Degree','FontSize',18)

ylabel('Number of Cells','FontSize',18)

elseif signal(2) == 1 && sum(signal) == 1

x=[-conversionFactor*maxCellLength./2 conversionFactor*maxCellLength./2];

x = repmat(x(1):x(2)*2/(size(lnumsort2,2)-3):x(2),size(lnumsort2,1),1);

y = repmat((1:size(lnumsort2,1)),size(lnumsort2,2)-2,1)';

dataToPlot = lnumsort2(1:end,3:end);

dataToPlot(dataToPlot==0) = NaN;

pcolor(x,y,flipud(dataToPlot)); colormap jet; colorbar;caxis([0 1]);shading flat;

xlabel('Distance From Midcell (\mum)','FontSize',18)

ylabel('Number of Cells','FontSize',18)

figure,scatter(lnumsort2(1:end,2),reverseNumlist)

xlabel('Constriction Degree','FontSize',18)

ylabel('Number of Cells','FontSize',18)

elseif sum(signal) == 2

%signal 1

x=[-conversionFactor*maxCellLength./2 conversionFactor*maxCellLength./2];

x = repmat(x(1):x(2)*2/(size(lnumsort1,2)-3):x(2),size(lnumsort1,1),1);

y = repmat((1:size(lnumsort1,1)),size(lnumsort1,2)-2,1)';

dataToPlot = lnumsort1(1:end,3:end);

dataToPlot(dataToPlot==0) = NaN;

pcolor(x,y,flipud(dataToPlot)); colormap jet; colorbar;caxis([0 1]);shading flat;

xlabel('Distance From Midcell (\mum)','FontSize',18)

ylabel('Number of Cells','FontSize',18)

figure,scatter(lnumsort1(1:end,2),reverseNumlist)

xlabel('Constriction Degree','FontSize',18)

ylabel('Number of Cells','FontSize',18)

%signal 2

figure;

x=[-conversionFactor*maxCellLength./2 conversionFactor*maxCellLength./2];

x = repmat(x(1):x(2)*2/(size(lnumsort2,2)-3):x(2),size(lnumsort2,1),1);

y = repmat((1:size(lnumsort2,1)),size(lnumsort2,2)-2,1)';

dataToPlot = lnumsort2(1:end,3:end);

dataToPlot(dataToPlot==0) = NaN;

pcolor(x,y,flipud(dataToPlot)); colormap jet; colorbar;caxis([0 1]);shading flat;

xlabel('Distance From Midcell (\mum)','FontSize',18)

ylabel('Number of Cells','FontSize',18)

figure,scatter(lnumsort2(1:end,2),reverseNumlist)

xlabel('Constriction Degree','FontSize',18)

ylabel('Number of Cells','FontSize',18)

end

catch err

if strcmpi(err.identifier,'MATLAB:catenate:dimensionMismatch')

warndlg('Choose a smaller number for max cell number parameter');

return;

else

warndlg('Make sure signal0 (phase profile) information is available in cellList');

end

end

case 'sort_by_constriction'

%%finds the maximum number of stepareas inside of a cell from the cellList

maxsizelarray=[];

try

sizel=[];

sizelarray=[];

for frame = frameList

for cellNum = 1:length(cellList.meshData{frame})

if ~isfield(cellList.meshData{frame}{cellNum},signalInfo) || eval('isempty(cellList.meshData{frame}{cellNum}.(signalInfo))') || cellList.meshData{frame}{cellNum}.length>maxCellLength

continue

end

maxsizelarray=[maxsizelarray length(cellList.meshData{frame}{cellNum}.lengthvector)]; %#ok<AGROW>

sizelarray= [sizelarray sizel]; %#ok<AGROW>

end

end

if isempty(maxsizelarray)

warndlg(['No field ' signalInfo ' recorded for this cell: Use Reuse meshes toggle button to compute ' signalInfo]);

return;

end

%using the maxima from above, a matrix consiting of zeros is created to be

%filled in by mesh intensities

maxsizel=max(maxsizelarray);

if maxCellLength > maxsizel;

maxCellLength = maxsizel;

end

maxsizel2 = ceil(maxsizel); if mod(maxsizel2,2)==0, maxsizel2=maxsizel2+1; end

maxsizel2a = maxsizel2/2+0.5;

relintarray1 = [ ];

relintarray2 = [ ];

lengthvectorarray=zeros(maxsizel,length(sizelarray)); %#ok

n=0;

cellLength=[];

DC=[];

%zeroarray is replaced with relative segment intensity data from the cell

for frame = frameList

for cellNum = 1:length(cellList.meshData{frame})

place=1; %#ok

if ~isfield(cellList.meshData{frame}{cellNum},signalInfo) || eval('isempty(cellList.meshData{frame}{cellNum}.(signalInfo))') || cellList.meshData{frame}{cellNum}.length>maxCellLength

continue

end

n = n+1;

%%calculates the fluorescent intensities in each segment normalized

%%by the area of that segment

if signal(1) == 1 && sum(signal) == 1

if length(cellList.meshData{frame}{cellNum}.signal1) > length(cellList.meshData{frame}{cellNum}.steparea)

cellList.meshData{frame}{cellNum}.relsignal1 = (cellList.meshData{frame}{cellNum}.signal1(1:length(cellList.meshData{frame}{cellNum}.steparea))./cellList.meshData{frame}{cellNum}.steparea);

else

cellList.meshData{frame}{cellNum}.relsignal1 = (cellList.meshData{frame}{cellNum}.signal1./cellList.meshData{frame}{cellNum}.steparea(1:length(cellList.meshData{frame}{cellNum}.signal1)));

end

%%segments are then normalized to the brightest segment so that

%%this sigment is represented as 1.

cellList.meshData{frame}{cellNum}.relint1 = (cellList.meshData{frame}{cellNum}.relsignal1./max(cellList.meshData{frame}{cellNum}.relsignal1));

elseif signal(2) == 1 && sum(signal) == 1

%%calculates the fluorescent intensities in each segment normalized

%%by the area of that segment

if length(cellList.meshData{frame}{cellNum}.signal2) > length(cellList.meshData{frame}{cellNum}.steparea)

cellList.meshData{frame}{cellNum}.relsignal2 = (cellList.meshData{frame}{cellNum}.signal2(1:length(cellList.meshData{frame}{cellNum}.steparea))./cellList.meshData{frame}{cellNum}.steparea);

else

cellList.meshData{frame}{cellNum}.relsignal2 = (cellList.meshData{frame}{cellNum}.signal2./cellList.meshData{frame}{cellNum}.steparea(1:length(cellList.meshData{frame}{cellNum}.signal2)));

end

%%segments are then normalized to the brightest segment so that

%%this sigment is represented as 1.

cellList.meshData{frame}{cellNum}.relint2 = (cellList.meshData{frame}{cellNum}.relsignal2./max(cellList.meshData{frame}{cellNum}.relsignal2));

elseif sum(signal) == 2

%%calculates the fluorescent intensities in each segment normalized

%%by the area of that segment

if length(cellList.meshData{frame}{cellNum}.signal1) > length(cellList.meshData{frame}{cellNum}.steparea)

cellList.meshData{frame}{cellNum}.relsignal1 = (cellList.meshData{frame}{cellNum}.signal1(1:length(cellList.meshData{frame}{cellNum}.steparea))./cellList.meshData{frame}{cellNum}.steparea);

else

cellList.meshData{frame}{cellNum}.relsignal1 = (cellList.meshData{frame}{cellNum}.signal1./cellList.meshData{frame}{cellNum}.steparea(1:length(cellList.meshData{frame}{cellNum}.signal1)));

end

%%segments are then normalized to the brightest segment so that

%%this sigment is represented as 1.

cellList.meshData{frame}{cellNum}.relint1 = (cellList.meshData{frame}{cellNum}.relsignal1./max(cellList.meshData{frame}{cellNum}.relsignal1));

%%calculates the fluorescent intensities in each segment normalized

%%by the area of that segment

if length(cellList.meshData{frame}{cellNum}.signal2) > length(cellList.meshData{frame}{cellNum}.steparea)

cellList.meshData{frame}{cellNum}.relsignal2 = (cellList.meshData{frame}{cellNum}.signal2(1:length(cellList.meshData{frame}{cellNum}.steparea))./cellList.meshData{frame}{cellNum}.steparea);

else

cellList.meshData{frame}{cellNum}.relsignal2 = (cellList.meshData{frame}{cellNum}.signal2./cellList.meshData{frame}{cellNum}.steparea(1:length(cellList.meshData{frame}{cellNum}.signal2)));

end

%%segments are then normalized to the brightest segment so that

%%this sigment is represented as 1.

cellList.meshData{frame}{cellNum}.relint2 = (cellList.meshData{frame}{cellNum}.relsignal2./max(cellList.meshData{frame}{cellNum}.relsignal2));

else

disp('provide information in signal variable')

return;

end

%%% A MOVING AVERAGE IS CALCULATED FOR EACH OF THE SEGMENTS TO FIND THE SINGLE BRIGHTEST SEGMENT AREA

cellList.meshData{frame}{cellNum}.meshavg=[];

if signal(1) == 1 && sum(signal) == 1

for place = 1:(length(cellList.meshData{frame}{cellNum}.relint1)-(numPixelsMovingAverage-1));

cellList.meshData{frame}{cellNum}.meshavg=[cellList.meshData{frame}{cellNum}.meshavg mean(cellList.meshData{frame}{cellNum}.relint1(place:(place+(numPixelsMovingAverage-1))))];

place=place+1; %#ok

end

elseif signal(2) == 1 && sum(signal) == 1

for place = 1:(length(cellList.meshData{frame}{cellNum}.relint2)-(numPixelsMovingAverage-1));

cellList.meshData{frame}{cellNum}.meshavg=[cellList.meshData{frame}{cellNum}.meshavg mean(cellList.meshData{frame}{cellNum}.relint2(place:(place+(numPixelsMovingAverage-1))))];

place=place+1;%#ok

end

end

%%WITH THE BRIGHTEST SEGMENT CALCULATED ABOVE WE CAN ORIENT THE

%%CELL SO THAT THE BRIGHTEST SEGMENT IS ON THE RIGHTS (i.e. WITH FtsZ BEING POLAR ON RIGHT(NEW POLE)

%%AND LARGER STALK CELL BIAS LETTING THE FtsZ RING BE ON THE RIGHT

%%AS WELL)

[~,maxavg]=max(cellList.meshData{frame}{cellNum}.meshavg);

if maxavg<=length(cellList.meshData{frame}{cellNum}.meshavg)/2+1;

if signal(1) ==1 && sum(signal) == 1

cellList.meshData{frame}{cellNum}.relint1=flipud(cellList.meshData{frame}{cellNum}.relint1);

elseif signal(2) == 1 && sum(signal) == 1

cellList.meshData{frame}{cellNum}.relint2=flipud(cellList.meshData{frame}{cellNum}.relint2);

elseif sum(signal) == 2

cellList.meshData{frame}{cellNum}.relint1=flipud(cellList.meshData{frame}{cellNum}.relint1);

cellList.meshData{frame}{cellNum}.relint2=flipud(cellList.meshData{frame}{cellNum}.relint2);

end

end

k = floor(cellList.meshData{frame}{cellNum}.length/2);

v=(1/(2*maxsizel)):(1/maxsizel):1;%#ok

if signal(1) == 1 && sum(signal) == 1

interpint1 = interp1(cellList.meshData{frame}{cellNum}.lengthvector-cellList.meshData{frame}{cellNum}.length/2,cellList.meshData{frame}{cellNum}.relint1,-k:k,'linear','extrap');

relintarray1(maxsizel2a-k:maxsizel2a+k,n)=interpint1;%#ok<AGROW>

elseif signal(2) == 1 && sum(signal) == 1

interpint2 = interp1(cellList.meshData{frame}{cellNum}.lengthvector-cellList.meshData{frame}{cellNum}.length/2,cellList.meshData{frame}{cellNum}.relint2,-k:k,'linear','extrap');

relintarray2(maxsizel2a-k:maxsizel2a+k,n)=interpint2; %#ok<AGROW>

elseif sum(signal) == 2

interpint1 = interp1(cellList.meshData{frame}{cellNum}.lengthvector-cellList.meshData{frame}{cellNum}.length/2,cellList.meshData{frame}{cellNum}.relint1,-k:k,'linear','extrap');

relintarray1(maxsizel2a-k:maxsizel2a+k,n)=interpint1;%#ok<AGROW>

interpint2 = interp1(cellList.meshData{frame}{cellNum}.lengthvector-cellList.meshData{frame}{cellNum}.length/2,cellList.meshData{frame}{cellNum}.relint2,-k:k,'linear','extrap');

relintarray2(maxsizel2a-k:maxsizel2a+k,n)=interpint2; %#ok<AGROW>

end

cellLength=[cellLength cellList.meshData{frame}{cellNum}.length]; %#ok<AGROW>

prf = cellList.meshData{frame}{cellNum}.signal0;

if isempty(prf), break; end

for i=1:2

prf = 0.5*prf + 0.25*(prf([1 1:end-1])+prf([2:end end]));

end

minima = [false reshape((prf(2:end-1)<prf(1:end-2))&(prf(2:end-1)<prf(3:end)),1,[]) false];

if isempty(minima) || sum(prf)==0

minsize=0;

ctpos = [];%#ok

else

im = find(minima);

minsize = 0;%#ok

ctpos = 0;%#ok

dh = [];

dhi = [];

hgt = [];

for k=1:length(im)

i=im(k);

half1 = prf(1:i-1);

half2 = prf(i+1:end);

dh1 = max(half1)-prf(i);

dh2 = max(half2)-prf(i);

dh(k) = min(dh1,dh2);%#ok

dhi(k) = mean([dh1 dh2]);%#ok

hgt(k) = prf(i)+dhi(k);%#ok

end

[~,i] = max(dh);

minsizeabs = dhi(i);

minsize = minsizeabs/hgt(i);

ctpos = im(i);%#ok

if isempty(minsize), minsize=0; end

end

DC = [DC minsize]; %#ok<AGROW>

end

end

% % cells length array is concatonated with the fluorescence matrix. This matrix is then sorted by length in ascending order

numlist=[1:1:n]; %#ok

reverseNumlist=[n:-1:1];%#ok

if signal(1) ==1 && sum(signal) == 1

lvint1=cat(2,cellLength',DC');

lvint1=cat(2,lvint1,relintarray1');

lnumsort1=sortrows(lvint1,[2]); %#ok

elseif signal(2) == 1 && sum(signal) == 1

lvint2=cat(2,cellLength',DC');

lvint2=cat(2,lvint2,relintarray2');

lnumsort2=sortrows(lvint2,[2]); %#ok

elseif sum(signal) == 2

lvint1=cat(2,cellLength',DC');

lvint1=cat(2,lvint1,relintarray1');

lnumsort1=sortrows(lvint1,[2]); %#ok

lvint2=cat(2,cellLength',DC');

lvint2=cat(2,lvint2,relintarray2');

lnumsort2=sortrows(lvint2,[2]); %#ok

end

%relative intensities are plotted accoring to a colormap

if signal(1) ==1 && sum(signal) == 1

x=[-.5*conversionFactor .5*conversionFactor];

x = repmat(x(1):x(2)*2/(size(lnumsort1,2)-3):x(2),size(lnumsort1,1),1);

y = repmat((1:size(lnumsort1,1)),size(lnumsort1,2)-2,1)';

dataToPlot = lnumsort1(1:end,3:end);

dataToPlot(dataToPlot==0) = NaN;

pcolor(x,y,flipud(dataToPlot)); colormap jet; colorbar;caxis([0 1]);shading flat;

xlabel('Distance From Midcell (\mum)','FontSize',18)

ylabel('Number of Cells','FontSize',18)

figure;scatter(lnumsort1(1:end,2),reverseNumlist)

xlabel('Constriction Degree','FontSize',18)

ylabel('Number of Cells','FontSize',18)

elseif signal(2) == 1 && sum(signal) == 1

x=[-.5*conversionFactor .5*conversionFactor];

x = repmat(x(1):x(2)*2/(size(lnumsort2,2)-3):x(2),size(lnumsort2,1),1);

y = repmat((1:size(lnumsort2,1)),size(lnumsort2,2)-2,1)';

dataToPlot = lnumsort2(1:end,3:end);

dataToPlot(dataToPlot==0) = NaN;

pcolor(x,y,flipud(dataToPlot)); colormap jet; colorbar;caxis([0 1]);shading flat;

xlabel('Distance From Midcell (\mum)','FontSize',18)

ylabel('Number of Cells','FontSize',18)

figure;scatter(lnumsort2(1:end,2),reverseNumlist)

xlabel('Constriction Degree','FontSize',18)

ylabel('Number of Cells','FontSize',18)

elseif sum(signal) == 2

x=[-.5*conversionFactor .5*conversionFactor];

x = repmat(x(1):x(2)*2/(size(lnumsort1,2)-3):x(2),size(lnumsort1,1),1);

y = repmat((1:size(lnumsort1,1)),size(lnumsort1,2)-2,1)';

dataToPlot = lnumsort1(1:end,3:end);

dataToPlot(dataToPlot==0) = NaN;

pcolor(x,y,flipud(dataToPlot)); colormap jet; colorbar;caxis([0 1]);shading flat;

xlabel('Distance From Midcell (\mum)','FontSize',18)

ylabel('Number of Cells','FontSize',18)

figure,scatter(lnumsort1(1:end,2),reverseNumlist)

xlabel('Constriction Degree','FontSize',18)

ylabel('Number of Cells','FontSize',18)

figure;

x=[-.5*conversionFactor .5*conversionFactor];

x = repmat(x(1):x(2)*2/(size(lnumsort2,2)-3):x(2),size(lnumsort2,1),1);

y = repmat((1:size(lnumsort2,1)),size(lnumsort2,2)-2,1)';

dataToPlot = lnumsort2(1:end,3:end);

dataToPlot(dataToPlot==0) = NaN;

pcolor(x,y,flipud(dataToPlot)); colormap jet; colorbar;caxis([0 1]);shading flat;

xlabel('Distance From Midcell (\mum)','FontSize',18)

ylabel('Number of Cells','FontSize',18)

figure,scatter(lnumsort2(1:end,2),reverseNumlist)

xlabel('Constriction Degree','FontSize',18)

ylabel('Number of Cells','FontSize',18)

end

catch err

if strcmpi(err.identifier,'MATLAB:catenate:dimensionMismatch')

warndlg('Choose a smaller number for max cell number parameter');

return;

else

warndlg('Make sure signal0 (phase profile) information is available in cellList');

end

end

case 'constriction_no_normalization'

%%finds the maximum number of stepareas inside of a cell from the cellList

maxsizelarray=[];

try

sizel=[];

sizelarray=[];

for frame = frameList

for cellNum = 1:length(cellList.meshData{frame})

if ~isfield(cellList.meshData{frame}{cellNum},signalInfo) || eval('isempty(cellList.meshData{frame}{cellNum}.(signalInfo))') || cellList.meshData{frame}{cellNum}.length>maxCellLength

continue

end

maxsizelarray=[maxsizelarray length(cellList.meshData{frame}{cellNum}.lengthvector)]; %#ok<AGROW>

sizelarray= [sizelarray sizel]; %#ok<AGROW>

end

end

%using the maxima from above, a matrix consiting of zeros is created to be

%filled in by mesh intensities

if isempty(maxsizelarray)

warndlg(['No field ' signalInfo ' recorded for this cell: Use Reuse meshes toggle button to compute ' signalInfo]);

return;

end

maxsizel=max(maxsizelarray);

if maxCellLength > maxsizel;

maxCellLength = maxsizel;

end

plotsizel=length(maxsizelarray);

maxsizel2 = ceil(maxsizel); if mod(maxsizel2,2)==0, maxsizel2=maxsizel2+1; end

maxsizel2a = maxsizel2/2+0.5;

relintarray1=zeros(maxsizel2,length(sizelarray));

lengthvectorarray=zeros(maxsizel,length(sizelarray));%#ok

n=0;

cellLength=[];

lengthvectorarray=[];%#ok

DC=[];

%zeroarray is replaced with relative segment intensity data from the cell

for frame = frameList

for cellNum = 1:length(cellList.meshData{frame})

place=1; %#ok

if ~isfield(cellList.meshData{frame}{cellNum},signalInfo) || eval('isempty(cellList.meshData{frame}{cellNum}.(signalInfo))') || cellList.meshData{frame}{cellNum}.length>maxCellLength

continue

end

n = n+1;

%%calculates the fluorescent intensities in each segment normalized

%%by the area of that segment

if signal(1) == 1 && sum(signal) == 1

if length(cellList.meshData{frame}{cellNum}.signal1) > length(cellList.meshData{frame}{cellNum}.steparea)

cellList.meshData{frame}{cellNum}.relsignal1 = (cellList.meshData{frame}{cellNum}.signal1(1:length(cellList.meshData{frame}{cellNum}.steparea))./cellList.meshData{frame}{cellNum}.steparea);

else

cellList.meshData{frame}{cellNum}.relsignal1 = (cellList.meshData{frame}{cellNum}.signal1./cellList.meshData{frame}{cellNum}.steparea(1:length(cellList.meshData{frame}{cellNum}.signal1)));

end

%%segments are then normalized to the brightest segment so that

%%this sigment is represented as 1.

cellList.meshData{frame}{cellNum}.relint1 = (cellList.meshData{frame}{cellNum}.relsignal1./max(cellList.meshData{frame}{cellNum}.relsignal1));

elseif signal(2) == 1 && sum(signal) == 1

%%calculates the fluorescent intensities in each segment normalized

%%by the area of that segment

if length(cellList.meshData{frame}{cellNum}.signal2) > length(cellList.meshData{frame}{cellNum}.steparea)

cellList.meshData{frame}{cellNum}.relsignal2 = (cellList.meshData{frame}{cellNum}.signal2(1:length(cellList.meshData{frame}{cellNum}.steparea))./cellList.meshData{frame}{cellNum}.steparea);

else

cellList.meshData{frame}{cellNum}.relsignal2 = (cellList.meshData{frame}{cellNum}.signal2./cellList.meshData{frame}{cellNum}.steparea(1:length(cellList.meshData{frame}{cellNum}.signal2)));

end

%%segments are then normalized to the brightest segment so that

%%this sigment is represented as 1.

cellList.meshData{frame}{cellNum}.relint2 = (cellList.meshData{frame}{cellNum}.relsignal2./max(cellList.meshData{frame}{cellNum}.relsignal2));

elseif sum(signal) == 2

%%calculates the fluorescent intensities in each segment normalized

%%by the area of that segment

if length(cellList.meshData{frame}{cellNum}.signal1) > length(cellList.meshData{frame}{cellNum}.steparea)

cellList.meshData{frame}{cellNum}.relsignal1 = (cellList.meshData{frame}{cellNum}.signal1(1:length(cellList.meshData{frame}{cellNum}.steparea))./cellList.meshData{frame}{cellNum}.steparea);

else

cellList.meshData{frame}{cellNum}.relsignal1 = (cellList.meshData{frame}{cellNum}.signal1./cellList.meshData{frame}{cellNum}.steparea(1:length(cellList.meshData{frame}{cellNum}.signal1)));

end

%%segments are then normalized to the brightest segment so that

%%this sigment is represented as 1.

cellList.meshData{frame}{cellNum}.relint1 = (cellList.meshData{frame}{cellNum}.relsignal1./max(cellList.meshData{frame}{cellNum}.relsignal1));

%%calculates the fluorescent intensities in each segment normalized

%%by the area of that segment

if length(cellList.meshData{frame}{cellNum}.signal2) > length(cellList.meshData{frame}{cellNum}.steparea)

cellList.meshData{frame}{cellNum}.relsignal2 = (cellList.meshData{frame}{cellNum}.signal2(1:length(cellList.meshData{frame}{cellNum}.steparea))./cellList.meshData{frame}{cellNum}.steparea);

else

cellList.meshData{frame}{cellNum}.relsignal2 = (cellList.meshData{frame}{cellNum}.signal2./cellList.meshData{frame}{cellNum}.steparea(1:length(cellList.meshData{frame}{cellNum}.signal2)));

end

%%segments are then normalized to the brightest segment so that

%%this sigment is represented as 1.

cellList.meshData{frame}{cellNum}.relint2 = (cellList.meshData{frame}{cellNum}.relsignal2./max(cellList.meshData{frame}{cellNum}.relsignal2));

else

disp('provide information in signal variable')

return;

end

%%% A MOVING AVERAGE IS CALCULATED FOR EACH OF THE SEGMENTS TO FIND THE SINGLE BRIGHTEST SEGMENT AREA

cellList.meshData{frame}{cellNum}.meshavg=[];

if signal(1) == 1 && sum(signal) == 1

for place = 1:(length(cellList.meshData{frame}{cellNum}.relint1)-(numPixelsMovingAverage-1));

cellList.meshData{frame}{cellNum}.meshavg=[cellList.meshData{frame}{cellNum}.meshavg mean(cellList.meshData{frame}{cellNum}.relint1(place:(place+(numPixelsMovingAverage-1))))];

place=place+1;%#ok

end

elseif signal(2) == 1 && sum(signal) == 1

for place = 1:(length(cellList.meshData{frame}{cellNum}.relint2)-(numPixelsMovingAverage-1));

cellList.meshData{frame}{cellNum}.meshavg=[cellList.meshData{frame}{cellNum}.meshavg mean(cellList.meshData{frame}{cellNum}.relint2(place:(place+(numPixelsMovingAverage-1))))];

place=place+1;%#ok

end

end

%%WITH THE BRIGHTEST SEGMENT CALCULATED ABOVE WE CAN ORIENT THE

%%CELL SO THAT THE BRIGHTEST SEGMENT IS ON THE RIGHTS (i.e. WITH FtsZ BEING POLAR ON RIGHT(NEW POLE)

%%AND LARGER STALK CELL BIAS LETTING THE FtsZ RING BE ON THE RIGHT

%%AS WELL)

k = floor(cellList.meshData{frame}{cellNum}.length/2);

v=1/(2*maxsizel):1/maxsizel:1;%#ok

if signal(1) == 1 && sum(signal) == 1

interpint1 = interp1(cellList.meshData{frame}{cellNum}.lengthvector-cellList.meshData{frame}{cellNum}.length/2,cellList.meshData{frame}{cellNum}.relint1,-k:k,'linear','extrap');

relintarray1(maxsizel2a-k:maxsizel2a+k,n)=interpint1;

elseif signal(2) == 1 && sum(signal) == 1

interpint2 = interp1(cellList.meshData{frame}{cellNum}.lengthvector-cellList.meshData{frame}{cellNum}.length/2,cellList.meshData{frame}{cellNum}.relint2,-k:k,'linear','extrap');

relintarray2(maxsizel2a-k:maxsizel2a+k,n)=interpint2; %#ok<AGROW>

elseif sum(signal) == 2

interpint1 = interp1(cellList.meshData{frame}{cellNum}.lengthvector-cellList.meshData{frame}{cellNum}.length/2,cellList.meshData{frame}{cellNum}.relint1,-k:k,'linear','extrap');

relintarray1(maxsizel2a-k:maxsizel2a+k,n)=interpint1;

interpint2 = interp1(cellList.meshData{frame}{cellNum}.lengthvector-cellList.meshData{frame}{cellNum}.length/2,cellList.meshData{frame}{cellNum}.relint2,-k:k,'linear','extrap');

relintarray2(maxsizel2a-k:maxsizel2a+k,n)=interpint2; %#ok<AGROW>

end

cellLength=[cellLength cellList.meshData{frame}{cellNum}.length]; %#ok<AGROW>

prf = cellList.meshData{frame}{cellNum}.signal0;

if isempty(prf),break; end

for i=1:2

prf = 0.5*prf + 0.25*(prf([1 1:end-1])+prf([2:end end]));

end

minima = [false reshape((prf(2:end-1)<prf(1:end-2))&(prf(2:end-1)<prf(3:end)),1,[]) false];

if isempty(minima) || sum(prf)==0

minsize=0;

ctpos = [];%#ok

else

im = find(minima);

minsize = 0;%#ok

ctpos = 0;%#ok

dh = [];

dhi = [];

hgt = [];

for k=1:length(im)

i=im(k);

half1 = prf(1:i-1);

half2 = prf(i+1:end);

dh1 = max(half1)-prf(i);

dh2 = max(half2)-prf(i);

dh(k) = min(dh1,dh2);%#ok

dhi(k) = mean([dh1 dh2]);%#ok

hgt(k) = prf(i)+dhi(k);%#ok

end

[~,i] = max(dh);

minsizeabs = dhi(i);

minsize = minsizeabs/hgt(i);

ctpos = im(i);%#ok

if isempty(minsize), minsize=0; end

end

DC = [DC minsize];%#ok<AGROW>

end

end

% % cells length array is concatonated with the fluorescence matrix. This matrix is then sorted by length in ascending order

numlist=[1:1:n];%#ok

reverseNumlist=[n:-1:1];%#ok

if signal(1) == 1 && sum(signal) == 1

lvint1=cat(2,cellLength',DC');

lvint1=cat(2,lvint1,relintarray1');

lnumsort1=sortrows(lvint1,[2]);%#ok

elseif signal(2) == 1 && sum(signal) == 1

lvint2=cat(2,cellLength',DC');

lvint2=cat(2,lvint2,relintarray2');

lnumsort2=sortrows(lvint2,[2]);%#ok

elseif sum(signal) == 2

lvint1=cat(2,cellLength',DC');

lvint1=cat(2,lvint1,relintarray1');

lnumsort1=sortrows(lvint1,[2]);%#ok

lvint2=cat(2,cellLength',DC');

lvint2=cat(2,lvint2,relintarray2');

lnumsort2=sortrows(lvint2,[2]);%#ok

end

if signal(1) == 1 && sum(signal) == 1

%relative intensities are plotted accoring to a colormap

x=[-conversionFactor*max(cellLength)./2 conversionFactor*max(cellLength)./2];

x = repmat(x(1):x(2)*2/(size(lnumsort1,2)-3):x(2),size(lnumsort1,1),1);

y = repmat((1:size(lnumsort1,1)),size(lnumsort1,2)-2,1)';

dataToPlot = lnumsort1(1:end,3:end);

dataToPlot(dataToPlot==0) = NaN;

pcolor(x,y,flipud(dataToPlot)); colormap jet; colorbar;caxis([0 1]);shading flat;

xlabel('Distance From Midcell (\mum)','FontSize',18)

ylabel('Number of Cells','FontSize',18)

% x2=[0 max(DC)];

figure,scatter(lnumsort1(1:end,2),reverseNumlist)

xlabel('Constriction Degree','FontSize',18)

ylabel('Number of Cells','FontSize',18)

elseif signal(2) == 1 && sum(signal) == 1

%relative intensities are plotted accoring to a colormap

x=[-conversionFactor*max(cellLength)./2 conversionFactor*max(cellLength)./2];

x = repmat(x(1):x(2)*2/(size(lnumsort2,2)-3):x(2),size(lnumsort2,1),1);

y = repmat((1:size(lnumsort2,1)),size(lnumsort2,2)-2,1)';

dataToPlot = lnumsort2(1:end,3:end);

dataToPlot(dataToPlot==0) = NaN;

pcolor(x,y,flipud(dataToPlot)); colormap jet; colorbar;caxis([0 1]);shading flat;

xlabel('Distance From Midcell (\mum)','FontSize',18)

ylabel('Number of Cells','FontSize',18)

% x2=[0 max(DC)];

figure,scatter(lnumsort2(1:end,2),reverseNumlist)

xlabel('Constriction Degree','FontSize',18)

ylabel('Number of Cells','FontSize',18)

elseif sum(signal) == 2

%relative intensities are plotted accoring to a colormap

x=[-conversionFactor*max(cellLength)./2 conversionFactor*max(cellLength)./2];

x = repmat(x(1):x(2)*2/(size(lnumsort1,2)-3):x(2),size(lnumsort1,1),1);

y = repmat((1:size(lnumsort1,1)),size(lnumsort1,2)-2,1)';

dataToPlot = lnumsort1(1:end,3:end);

dataToPlot(dataToPlot==0) = NaN;

pcolor(x,y,flipud(dataToPlot)); colormap jet; colorbar;caxis([0 1]);shading flat;

xlabel('Distance From Midcell (\mum)','FontSize',18)

ylabel('Number of Cells','FontSize',18)

% x2=[0 max(DC)];

figure,scatter(lnumsort1(1:end,2),reverseNumlist)

xlabel('Constriction Degree','FontSize',18)

ylabel('Number of Cells','FontSize',18)

%relative intensities are plotted accoring to a colormap

figure;

x=[-conversionFactor*max(cellLength)./2 conversionFactor*max(cellLength)./2];

x = repmat(x(1):x(2)*2/(size(lnumsort2,2)-3):x(2),size(lnumsort2,1),1);

y = repmat((1:size(lnumsort2,1)),size(lnumsort2,2)-2,1)';

dataToPlot = lnumsort2(1:end,3:end);

dataToPlot(dataToPlot==0) = NaN;

pcolor(x,y,flipud(dataToPlot)); colormap jet; colorbar;caxis([0 1]);shading flat;

xlabel('Distance From Midcell (\mum)','FontSize',18)

ylabel('Number of Cells','FontSize',18)

% x2=[0 max(DC)];

figure,scatter(lnumsort2(1:end,2),reverseNumlist)

xlabel('Constriction Degree','FontSize',18)

ylabel('Number of Cells','FontSize',18)

end

catch err

if strcmpi(err.identifier,'MATLAB:catenate:dimensionMismatch')

warndlg('Choose a smaller number for max cell number parameter');

return;

else

warndlg('Make sure signal0(phase profile) information is available in cellList');

end

end

otherwise

disp('descriptor variable must contain one of the following values')

descriptorValues %#ok

end

end

**shadedErrorBar.** This function creates shaded error bars based on previous analysis. This function is utilized in “AB_PlotExtractDataNorm”.

function varargout=shadedErrorBar(x,y,errBar,varargin)

% generate continuous error bar area around a line plot

%

% function H=shadedErrorBar(x,y,errBar, ...)

%

% Purpose

% Makes a 2-d line plot with a pretty shaded error bar made

% using patch. Error bar color is chosen automatically.

%

%

% Inputs (required)

% x - vector of x values [optional, can be left empty]

% y - vector of y values or a matrix of n observations by m cases

% where m has length(x);

% errBar - if a vector we draw symmetric errorbars. If it has a size

% of [2,length(x)] then we draw asymmetric error bars with

% row 1 being the upper bar and row 2 being the lower bar

% (with respect to y -- see demo). ** alternatively **

% errBar can be a cellArray of two function handles. The

% first defines statistic the line should be and the second

% defines the error bar.

%

% Inputs (optional, param/value pairs)

% 'lineProps' - ['-k' by default] defines the properties of

% the data line. e.g.:

% 'or-', or {'-or','markerfacecolor',[1,0.2,0.2]}

% 'transparent' - [true by default] if true, the shaded error

% bar is made transparent. However, for a transparent

% vector image you will need to save as PDF, not EPS,

% and set the figure renderer to "painters". An EPS

% will only be transparent if you set the renderer

% to OpenGL, however this makes a raster image.

% 'patchSaturation'- [0.2 by default] The saturation of the patch color.

%

%

%

% Outputs

% H - a structure of handles to the generated plot objects.

%

%

% Examples:

% y=randn(30,80);

% x=1:size(y,2);

%

% 1)

% shadedErrorBar(x,mean(y,1),std(y),'lineprops','g');

%

% 2)

% shadedErrorBar(x,y,{@median,@std},'lineprops',{'r-o','markerfacecolor','r'});

%

% 3)

% shadedErrorBar([],y,{@median,@(x) std(x)*1.96},'lineprops',{'r-o','markerfacecolor','k'});

%

% 4)

% Overlay two transparent lines:

% clf

% y=randn(30,80)*10;

% x=(1:size(y,2))-40;

% shadedErrorBar(x,y,{@mean,@std},'lineprops','-r','transparent',1);

% hold on

% y=ones(30,1)*x; y=y+0.06*y.^2+randn(size(y))*10;

% shadedErrorBar(x,y,{@mean,@std},'lineprops','-b','transparent',1);

% hold off

%

%

% Rob Campbell - November 2009

%%%%%%%%%%%%%%%%%%%%%%%%%%%%%%%%%%%%%%%%%%%%%%%%%%%%%%%%%%%%%

% Parse input arguments

narginchk(3,inf)

params = inputParser;

params.CaseSensitive = false;

params.addParameter('lineProps', '-k', @(x) ischar(x) | iscell(x));

if (sum( size(ver('MATLAB'))) > 0 )

params.addParameter('transparent', true, @(x) islogical(x) || x==0 || x==1);

elseif (sum( size(ver('Octave'))) > 0 )

params.addParameter('transparent', false, @(x) islogical(x) || x==0 || x==1);

end

params.addParameter('patchSaturation', 0.2, @(x) isnumeric(x) && x>=0 && x<=1);

params.parse(varargin{:});

%Extract values from the inputParser

lineProps = params.Results.lineProps;

transparent = params.Results.transparent;

patchSaturation = params.Results.patchSaturation;

if ~iscell(lineProps), lineProps={lineProps}; end

%Process y using function handles if needed to make the error bar dynamically

if iscell(errBar)

fun1=errBar{1};

fun2=errBar{2};

errBar=fun2(y);

y=fun1(y);

else

y=y(:).';

end

if isempty(x)

x=1:length(y);

elseif sum( size(ver('MATLAB'))) > 0

x=x(:).';

end

%Make upper and lower error bars if only one was specified

if length(errBar)==length(errBar(:))

errBar=repmat(errBar(:)',2,1);

else

s=size(errBar);

f=find(s==2);

if isempty(f), error('errBar has the wrong size'), end

if f==2, errBar=errBar'; end

end

% Check for correct x, errbar formats

x_size = size(x);

if (length(x) ~= length(errBar) && sum( size(ver('MATLAB'))) > 0 )

error('length(x) must equal length(errBar)')

elseif( ( length(x) ~= length(errBar) && checkOctave_datestr(x) == false ) ...

&& sum( size(ver('Octave'))) > 0 )

error('length(x) must equal length(errBar) or x must have valid datestr')

end

%Log the hold status so we don't change

initialHoldStatus=ishold;

if ~initialHoldStatus, hold on, end

H = makePlot(x,y,errBar,lineProps,transparent,patchSaturation);

if ~initialHoldStatus, hold off, end

if nargout==1

varargout{1}=H;

end

function H = makePlot(x,y,errBar,lineProps,transparent,patchSaturation)

%%%%%%%%%%%%%%%%%%%%%%%%%%%%%%%%%%%%%%%%%%%%%%%%%%%%%%%%%%%%%

% Determine host application

if (sum( size(ver('MATLAB'))) > 0 )

hostName = 'MATLAB';

elseif (sum(size(ver('Octave'))) > 0)

hostName = 'Octave';

end % if

% Plot to get the parameters of the line

if hostName == 'MATLAB'

H.mainLine=plot(x,y,lineProps{:});

elseif hostName == 'Octave'

boolxDatestr = checkOctave_datestr(x);

if boolxDatestr

x = datenum(x);

x = x(:).';

H.mainLine=plot(x,y,lineProps{:});

datetick(gca);

else

H.mainLine=plot(x,y,lineProps{:});

end

end

% Tag the line so we can easily access it

H.mainLine.Tag = 'shadedErrorBar_mainLine';

% Work out the color of the shaded region and associated lines.

% Here we have the option of choosing alpha or a de-saturated

% solid colour for the patch surface.

mainLineColor=get(H.mainLine,'color');

edgeColor=mainLineColor+(1-mainLineColor)*0.55;

if transparent

faceAlpha=patchSaturation;

patchColor=mainLineColor;

else

faceAlpha=1;

patchColor=mainLineColor+(1-mainLineColor)*(1-patchSaturation);

end

%Calculate the error bars

uE=y+errBar(1,:);

lE=y-errBar(2,:);

%Make the patch (the shaded error bar)

yP=[lE,fliplr(uE)];

xP=[x,fliplr(x)];

%remove nans otherwise patch won't work

xP(isnan(yP))=[];

yP(isnan(yP))=[];

if isdatetime(x) && strcmp(hostName,'MATLAB')

H.patch=patch(datenum(xP),yP,1);

else

H.patch=patch(xP,yP,1);

end

set(H.patch,'facecolor',patchColor, ...

'edgecolor','none', ...

'facealpha',faceAlpha, ...

'HandleVisibility', 'off', ...

'Tag', 'shadedErrorBar_patch')

%Make pretty edges around the patch.

H.edge(1)=plot(x,lE,'-');

H.edge(2)=plot(x,uE,'-');

set([H.edge], 'color',edgeColor, ...

'HandleVisibility','off', ...

'Tag', 'shadedErrorBar_edge')

% Ensure the main line of the plot is above the other plot elements

if hostName == 'MATLAB'

if strcmp(get(gca,'YAxisLocation'),'left') %Because re-ordering plot elements with yy plot is a disaster

uistack(H.mainLine,'top')

end

elseif hostName == 'Octave'

% create the struct from scratch by temp.

H = struct('mainLine', H.mainLine, ...

'patch', H.patch, ...

'edge', H.edge);

end

function boolDate = checkOctave_datestr(x)

%% Simple try/catch for casting datenums, requireing valid datestr

boolDate = true;

try

datenum(x)

catch

boolDate = false;

end
